# On Sackin’s original proposal: the variance of the leaves’ depths as a phylogenetic balance index

**DOI:** 10.1186/s12859-020-3405-1

**Published:** 2020-04-23

**Authors:** Tomás M. Coronado, Arnau Mir, Francesc Rosselló, Lucía Rotger

**Affiliations:** 1 Department of Mathematics and Computer Science, University of the Balearic Islands, Palma, E-07122 Spain; 2 Balearic Islands Health Research Institute (IdISBa), Palma, E-07010 Spain; 3 Dept. of Mathematics and Computing, University of La Rioja, Logroño, E-26004 Spain

**Keywords:** Phylogenetic tree, Balance index, Sackin index, Total cophenetic index, Uniform model, Yule model, Maximally balanced tree

## Abstract

**Background:**

The *Sackin indexS* of a rooted phylogenetic tree, defined as the sum of its leaves’ depths, is one of the most popular balance indices in phylogenetics, and Sackin’s paper (Syst Zool 21:225–6, 1972) is usually cited as the source for this index. However, what Sackin actually proposed in his paper as a measure of the imbalance of a rooted tree was not the sum of its leaves’ depths, but their “variation”. This proposal was later implemented as the variance of the leaves’ depths by Kirkpatrick and Slatkin in (Evolution 47:1171–81, 1993), where they also posed the problem of finding a closed formula for its expected value under the Yule model. Nowadays, Sackin’s original proposal seems to have passed into oblivion in the phylogenetics literature, replaced by the index bearing his name, which, in fact, was introduced a decade later by Sokal.

**Results:**

In this paper we study the properties of the variance of the leaves’ depths, *V*, as a balance index. Firstly, we prove that the rooted trees with *n* leaves and maximum *V* value are exactly the combs with *n* leaves. But although *V* achieves its minimum value on every space $\mathcal {BT}_{n}$ of bifurcating rooted phylogenetic trees with *n*≤183 leaves at the so-called “maximally balanced trees” with *n* leaves, this property fails for almost every *n*≥184. We provide then an algorithm that finds the trees in $\mathcal {BT}_{n}$ with minimum *V* value in time *O*(*n* log(*n*)). Secondly, we obtain closed formulas for the expected *V* value of a bifurcating rooted tree with any number *n* of leaves under the Yule and the uniform models and, as a by-product of the computations leading to these formulas, we also obtain closed formulas for the variance under the uniform model of the Sackin index and the total cophenetic index (Mir et al., Math Biosci 241:125–36, 2013) of a bifurcating rooted tree, as well as of their covariance, thus filling this gap in the literature.

**Conclusion:**

The phylogenetics community has been wise in preferring the sum *S*(*T*) of the leaves’ depths of a phylogenetic tree *T* over their variance *V*(*T*) as a balance index, because the latter does not seem to capture correctly the notion of balance of large bifurcating rooted trees. But it is still a valid and useful shape index.

## Background

In the last decades there has been an increasing interest in the definition and study of indices quantifying properties of trees. Besides the research on this topic carried out within the framework of *Quantitative Graph Theory* [12], the main motivation has come from the field of phylogenetics, through the application of such indices in the description and comparison of phylogenetic trees. In effect, there is the commonly accepted belief that the characteristics of the branching pattern of a phylogenetic tree reflect the properties and tendencies of the evolutionary processes that have produced it [19, 34]. As a consequence, the shape of a phylogenetic tree is thought to offer clues to the features of the evolutionary processes underlying it and in particular to provide a ground to test hypothesis on these features [14, Chap. 33]. This has motivated the introduction of many *tree shape indices* on phylogenetic trees, related only to their topology and not taking into account branch lengths or the actual taxa on their leaves. These indices have then been used to test evolutionary hypothesis and models [3, 13, 21, 24, 27, 33, 34, 40, 44] as well as in other applications [2, 8, 18, 30, 42].

Although considerations about the shape of phylogenetic trees go back at least to the late 1960s [37], since the early 1980s this research has focused mainly on their *balance* [41], intuitively understood as the tendency of the descendant taxa of any internal node to split into clades of similar size. In principle, the imbalance of a phylogenetic tree reflects the propensity of evolutionary events to occur along specific lineages, although in some cases it may also be due simply to a bias in the method used to build it [35, 41].

The degree of balance of a phylogenetic tree is usually measured using *balance indices*. Several such indices have been proposed [9, 11, 16, 27, 29, 31, 32, 39, 41] (see also the section “Measures of overall asymmetry” in [14, Chap. 33]) and Shao and Sokal [41, p. 273] explicitly advised to “choose more than one index” to quantify the balance of a tree. One of the most popular and widely used is the so-called *Sackin indexS*, defined as the sum of the *depths* (i.e., their distance from the root) of the leaves of the tree. Although the paper [39] by Sackin is usually cited as the source for this index, to our knowledge it was used for the first time by Sokal in [42] and it was not called “Sackin’s index” until the paper [41] by Shao and Sokal on tree balance.

**Fig. 1:**
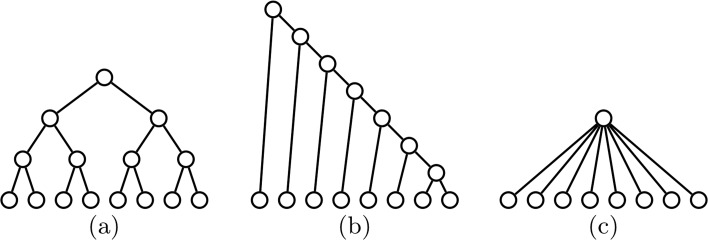
**a** A fully symmetric tree with 8 leaves. **b** A comb with 8 leaves. **c** A rooted star with 8 leaves

However, against what the index bearing his name would indicate, Sackin did not propose to use the sum of the leaves’ depths as a measure of the balance of a rooted tree (or, rather, of its imbalance). What the author of [39] actually did was to point out that more balanced trees tended to have lower (maximum) depth and smaller variation of the leaves’ depths. To make his point, he compared these properties on a fully symmetric tree with 8 leaves and a comb with the same number of leaves (see (a) and (b) in Fig. 1). The fully symmetric tree has the smallest possible depth for a bifurcating tree with 8 leaves, which is 3, while the comb has the largest possible such depth, 7. As to the variation of the leaves’ depths, all leaves of the fully symmetric tree have the same depth, while all leaves in the comb have different depth, except for the pair forming the deepest cherry; in fact, as we shall prove in Theorem 1, the comb with *n* leaves turns out to have the largest variance of the leaves’ depths among all rooted trees with *n* leaves.

It is clear that the depth *δ*(*T*) of a tree *T* is a very coarse shape index, with a small range of values for any number of leaves, so it is easy to understand that it did not crystallize as a balance index. But Sackin’s second proposal, the degree of variation of the leaves’ depths, seems a very reasonable idea. It was later implemented by Kirkpatrick and Slatkin in [27] as the variance of the leaves’ depths, which we shall denote henceforth by *V*, and these authors showed empirically that its power is similar and sometimes higher than that of *S* in some statistical tests with alternative hypothesis representing “this tree is not random”. Yet, although *V* was used as a shape index in a few early studies [25–27] and it was even collected in the section “Measures of overall asymmetry” in Felsenstein’s book [14], it seems to have passed into oblivion, and Shao and Sokal’s proposal of using the sum of the leaves’ depths, and attributing it to Sackin, has been preferred by the phylogenetics community.

One of the problems that we consider in this paper is to determine to which extent *V* measures the degree of imbalance of a rooted tree. To do that, we study which trees achieve the maximum and the minimum values of *V* for any given number of leaves *n*: they would play the role of the least and the most balanced rooted trees with *n* leaves according to *V*. With respect to the maximum value of *V*, it is achieved at the combs, which are the trees classified as most imbalanced by other balance indices like the Sackin index [15], the Colless index [32], the total cophenetic index [31], the number of cherries (the comb is the only bifurcating tree with a single cherry), or the rooted quartet index [11]. With respect to its minimum value, in the case of multifurcating trees it is achieved, among other trees, at the rooted star trees (cf. Fig. 1c), which have all leaves of depth 1 and hence null variance, in agreement again with all other balance indices for multifurcating trees like the Sackin index, the total cophenetic index, the rooted quartet index, or the Colless-like indices introduced in [32]. So far, so good, except for the fact that, actually, any multifurcating tree with all its leaves of the same depth has minimum *V* value, 0, independently on its shape, which implies that *V* cannot be used in the context of taxonomic trees, where all leaves have the same depth: one less than the number of taxonomic ranks in the tree. By the way, *V* shares this drawback with the Sackin index, because the value of the latter on a taxonomic tree only depends on its number of leaves and of taxonomic ranks, and not on its shape.

The main problem lies in the bifurcating rooted trees with minimum *V* value, which would correspond to the bifurcating rooted trees classified as most balanced by *V*. On the positive side, since the fully symmetric bifurcating trees where the number of leaves is a power of 2 have all their leaves of the same depth, their *V* value is the minimum, 0, in agreement with their full balance. And for each number *n*≤183 of leaves, the minimum *V* value among all rooted bifurcating trees with *n* leaves is reached at the *maximally balanced trees*, those bifurcating trees where the descendant leaves of every internal node split among its pair of children into two subsets of cardinalities differing at most by 1. This is in agreement with other balance indices, like the Sackin index [15], the Colless index [10], the total cophenetic index [31], and the rooted quartets index [11], that classify as most balanced these maximally balanced trees (tied with other trees in the case of the Sackin and Colless indices, and only them in the case of the other two indices). These maximally balanced trees were called “the most balanced trees” by Shao and Sokal [41].

**Fig. 2:**
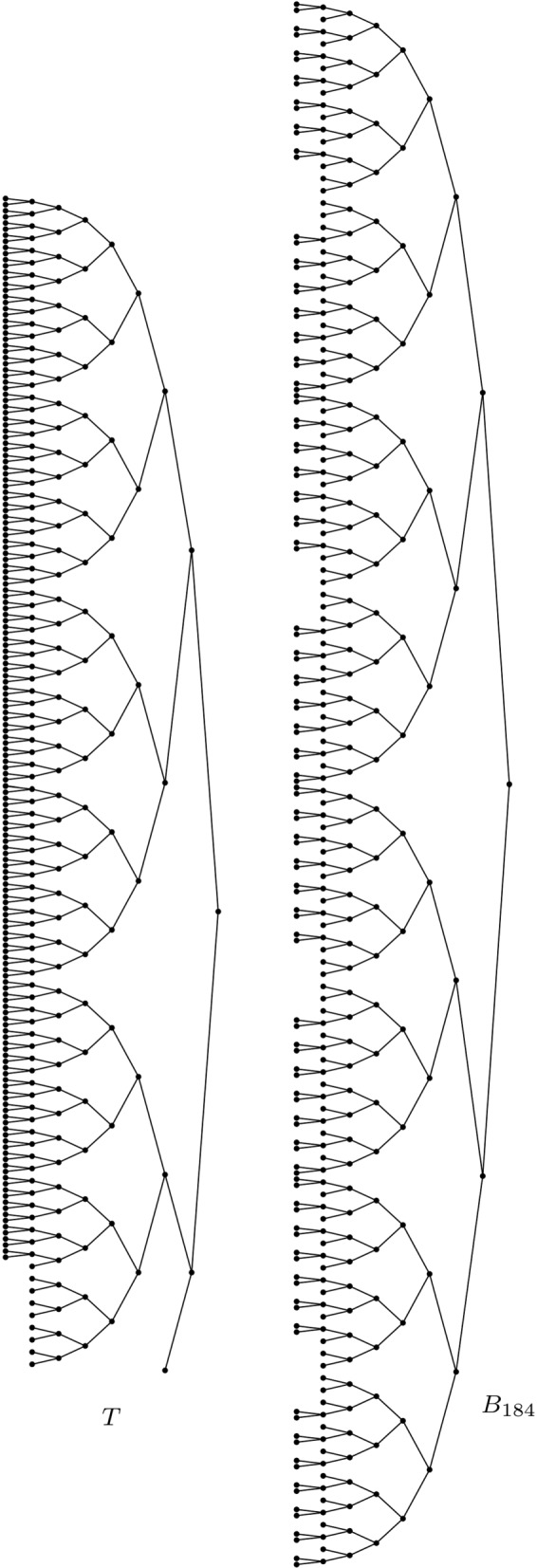
The leaves’ depths of the left-hand side tree $T\in \mathcal {BT}^{*}_{184}$ have smaller variance than those of the right-hand side maximally balanced tree *B*_184_

The trouble with *V* starts with *n*=184. The bifurcating tree with 184 leaves and minimum *V* value is not maximally balanced (see Fig. 2). And it turns out that, as *n* grows to *∞*, the fraction of numbers *n* of leaves for which the minimum *V* value is achieved at the maximally balanced tree tends to 0 (see Theorem 3). So, for large numbers of leaves, the bifurcating trees that are most balanced according to *V* are almost never the maximally balanced ones. In our opinion, this result is quite surprising and counterintuitive. Indeed, we prove in Proposition 5 that a multiset of leaves’ depths is realized by a maximally balanced tree if, and only if, its entries are either constant (which corresponds to the fully symmetric trees) or they take two different values differing by 1 unit, and our intuition told us (and, probably, Sackin) that these multisets containing only depths equal to *δ* (the depth of the tree) and *δ*−1 were those presenting the lowest variation, that is, the smallest variance. Our work shows that it is almost never the case.

This drawback makes *V* unsuitable as a measure of the imbalance of bifurcating trees with large number of leaves. But *V* can still be used with this purpose on small bifurcating trees and, in general, as a shape index, for instance to test evolutionary hypothesis. As we have already mentioned, Kirkpatrick and Slatkin analyzed its power in some specific tests of this type in [27], where they showed it to be comparable and sometimes higher than that of the Sackin and Colless indices. In a subsequent study by Agapow and Purvis [1] that extended Kirkpatrick and Slatkin’s tests to other more biologically motivated models, *V* was also classified, together with the Sackin and Colless indices, in the leading group of balance indices with respect to their power in detecting some types of nonrandom diversification. In a later paper by Blum and François [3], *V* was not included in the set of tested indices and the Sackin index was shown to be very powerful in rejecting the Yule model against biased speciation models that generate either very imbalanced or very balanced trees, but not so powerful with models generating less evidently balanced or imbalanced trees. Let us also mention that *V* has also been considered in two experiments testing the resolution of several balance indices, i.e., their capacity to discriminate between similar and different tree shapes, for different specific measures of shape similarity [23, 28]. In both cases it was classified in the top-three set of balance indices, again together with the Sackin and the Colless indices.

In the application of a balance index to test evolutionary models, it is convenient to have closed formulas for its expected value under different probabilistic models of phylogenetic trees [3]. This was already pointed out by Kirkpatrick and Slatkin in [27], where they complained that “its expectation [of *V* under the Yule model] is not known analytically” and they had to estimate it by simulations. So, in this paper we also provide closed formulas for the expected value of *V* under the Yule, or Equal-Rate Markov, model [22, 46] and the uniform, or Proportional to Distinguishable Arrangements, model [7, 38] for rooted bifurcating phylogenetic trees (see Theorems 4 and 5, respectively). Additionally, as a by-product of the tools developed to prove these theorems, we also obtain closed formulas for the variance under the uniform model of the Sackin index and the total cophenetic index as well as of their covariance (see Theorem 6). It is worth recalling that, for the variance of the Sackin index under the uniform model, only a recursive formula [36] and its asymptotic behaviour [4] were known so far.

Since the proofs of most results in this paper are quite long and technical, in order not to lose the thread of the paper we have moved almost all of them, as well as all the auxiliary lemmas used in them, to the Additional file 1. Besides, all the data sets and scripts related to this paper are available at the GitHub repository https://github.com/biocom-uib/var_depths.

### Notations

#### Trees

In this paper, by a *treeT* we always mean a rooted tree without out-degree 1 nodes, understood as a directed graph with its arcs pointing away from the root. We shall denote by *L*(*T*) the set of *leaves* (i.e., of out-degree 0 nodes) of *T*; the nodes in *T* that are not leaves are called *internal*. A tree is *bifurcating* when all its internal nodes have out-degree 2; when we want to emphasize that a tree need not be bifurcating, we shall call it *multifurcating*. We shall always consider two isomorphic trees as equal, and we shall denote by $\mathcal {T}^{*}_{n}$ and $\mathcal {BT}^{*}_{n}$ the spaces of (isomorphism classes of) multifurcating and bifurcating trees with *n* leaves, respectively.

Let *T* be a tree. If (*u*,*v*) is an arc in *T*, we say that the node *v* is a *child* of the node *u* and also that *u* is the *parent* of *v*. When two nodes have the same parent, we say that they are *sibling*. When there exists a path from *u* to *v* in *T*, we say that *v* is a *descendant* of *u* and also that *u* is an *ancestor* of *v*. The *lowest common ancestor* of a pair of nodes *u*,*v* in *T* is the unique common ancestor of them that is a descendant of every common ancestor of them. The *subtree* of *Trooted* at a node *v* is the subgraph of *T* induced by the descendants of *v*.

The *depth* *δ*_*T*_(*v*) of a node *v* in *T* is the length (in number of arcs) of the unique path from the root of *T* to *v*. We shall denote by *δ*(*T*) the *depth* of *T*, that is, the largest depth of any leaf in it. Furthermore, we shall denote by *Δ*(*T*) the multiset of depths of the leaves of *T*, where each depth appears with multiplicity the number of leaves with this depth, and we shall say that two trees $T,T'\in \mathcal {T}^{*}_{n}$ are *depth-equivalent* when *Δ*(*T*)=*Δ*(*T*^′^).

A *comb* is a bifurcating tree such that all its internal nodes have a leaf child: cf. Fig. 1b. We shall denote the comb with *n* leaves by *K*_*n*_. A *rooted star* is a tree of depth 1: cf. Fig. 1c. We shall denote the rooted star with *n* leaves by *RS*_*n*_.

**Fig. 3:**
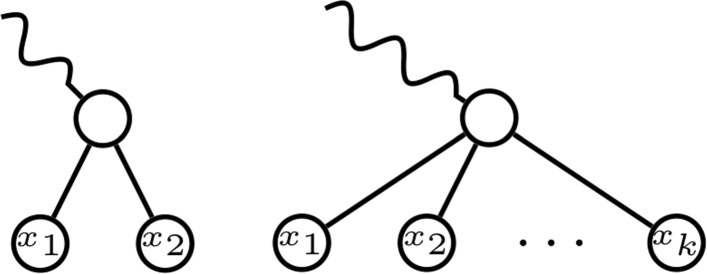
A cherry (left) and a *k*-fan (right)

A *k-fan* of a tree is a rooted subtree of it that is a rooted star with *k* leaves; see Fig. 3. A *cherry* is a 2-fan. To simplify the language, we shall often identify a *k*-fan (or a cherry) with its leaves.

#### Phylogenetic trees

A *phylogenetic tree* on a set *X* is a (rooted) tree with its leaves bijectively labeled in *X*. We shall usually identify the leaves of a phylogenetic tree with their labels. A phylogenetic tree is *bifurcating* when its underlying tree is bifurcating. We shall denote by $\mathcal {T}(X)$ and $\mathcal {BT}(X)$ the spaces of (isomorphism classes of) multifurcating and bifurcating phylogenetic trees on *X*, respectively. If |*X*|=*n*, there exists a forgetful mapping $\pi :\mathcal {T}(X)\to \mathcal {T}_{n}^{*}$ that sends every phylogenetic tree *T* on *X* to its underlying tree: we shall call *π*(*T*) the *shape* of *T*. Notice that *π* maps $\mathcal {BT}(X)$ onto $\mathcal {BT}_{n}^{*}$. When the specific set of labels *X* is irrelevant and only its cardinality |*X*|=*n* matters, we shall identify *X* with the set [ *n*]={1,…,*n*} and then we shall write $\mathcal {T}_{n}$ and $\mathcal {BT}_{n}$ instead of $\mathcal {T}(X)$ and $\mathcal {BT}(X)$, respectively; in this case, we shall speak about *phylogenetic trees with n leaves*. Every bijection *X*⇔[ *n*] induces bijections $\mathcal {T}(X)\leftrightarrow \mathcal {T}_{n}$ and $\mathcal {BT}(X)\leftrightarrow \mathcal {BT}_{n}$ that preserve the shapes of the trees.

#### Balance indices

Let $T\in \mathcal {T}^{*}_{n}$. Its *Sackin index* *S*(*T*) is the sum of the depths of its leaves [39, 41]:

$$S(T)=\sum_{x\in L(T)} \delta_{T}(x). $$

We shall denote the mean depth of the leaves of *T* by $\widehat {S}(T)$:

$$\widehat{S}(T)=\frac{1}{n}\sum_{x\in L(T)} \delta_{T}(x). $$

Given two different leaves *x*,*y*∈*L*(*T*), their *cophenetic value* *φ*_*T*_(*x*,*y*) is the depth of their lowest common ancestor [43]. The *total cophenetic index* of *T*, *Φ*(*T*), is then the sum of the cophenetic values of its pairs of different leaves [31]:

$$ \Phi(T)=\sum_{x,y\in L(T)\atop x\neq y} \varphi_{T}(x,y). $$

#### Probabilistic models of bifurcating phylogenetic trees

A *probabilistic model of bifurcating phylogenetic trees* *P*_*n*_,*n*≥1, is a family of probability mappings $P_{n}:\mathcal {BT}_{n}\to [\!0,1]$, each one sending each phylogenetic tree in $\mathcal {BT}_{n}$ to its probability under this model. Later in this paper we shall be concerned with two popular probabilistic models of bifurcating phylogenetic trees arising from stochastic models of phylogenetic trees’ growth: the Yule model [22, 46] and the uniform model [7, 38].

The *Yule*, or *Equal-Rate Markov*, model produces bifurcating phylogenetic trees on [ *n*] through the following stochastic process: starting with a single node, at every step a leaf is chosen randomly and uniformly and it is replaced by a cherry; when the desired number *n* of leaves is reached, the labels are assigned randomly and uniformly to these leaves. This stochastic model defines a probabilistic model of phylogenetic trees by assigning to each $T\in \mathcal {BT}_{n}$ the probability *P*_*Y*,*n*_(*T*) of being obtained through this process. This probability is (see, for instance, [45, Prop. 3.2])

1 $$ P_{Y,n}(T)=\frac{2^{n-1}}{n!}\prod_{v\in V_{int}(T)}\frac{1}{\kappa_{T}(v)-1},   $$

where *V*_*int*_(*T*) denotes the set of internal nodes of *T* and, for every internal node *v*, *κ*_*T*_(*v*) denotes its number of descendant leaves.

With respect to the *uniform*, or *Proportional to Distinguishable Arrangements*, model, it produces bifurcating phylogenetic trees on [ *n*] through the following stochastic process: starting with a node labeled 1, at the *k*-th step a new arc ending in the leaf labeled *k*+1 is added either to a new root (whose other child will be, then, the original root) or to some arc, with all possible locations of this new pendant arc equiprobable. It turns out that all phylogenetic trees *T* in $\mathcal {BT}_{n}$ are obtained through this process with the same probability. Then, since for every $n\geq 1, |\mathcal {BT}_{n}|=(2n-3)!!=(2n-3)(2n-5)\cdots 3 \cdot 1$ (with the convention (−1)!!=1) [14, Ch. 3], this probability is

2 $$ P_{U,n}(T)=\frac{1}{(2n-3)!!}.   $$

Given a mapping $I:\bigcup _{n\geq 1} \mathcal {BT}_{n}\to \mathbb {R}$, we shall denote by *I*_*n*_ the random variable that takes a phylogenetic tree $T\in \mathcal {BT}_{n}$ and gives *I*(*T*), and we shall denote by *E*_*Y*_(*I*_*n*_) and *E*_*U*_(*I*_*n*_) the expected value of *I*_*n*_ under the Yule and the uniform models, respectively; i.e.

$$\begin{array}{@{}rcl@{}} E_{Y}(I_{n})&=&\sum_{T\in \mathcal{BT}_{n}} I(T)\cdot P_{Y,n}(T),\quad\\ E_{U}(I_{n})&=&\sum_{T\in \mathcal{BT}_{n}} I(T)\cdot P_{U,n}(T).\end{array} $$

## Results

This paper’s main focus is the *variance* of the depths of the leaves of a tree $T\in \mathcal {T}^{*}_{n}$, which we denote henceforth by *V*(*T*):

$$V(T)=\frac{1}{n}\sum_{x\in L(T)}\left(\delta_{T}(x)-\widehat{S}(T)\right)^{2}. $$

Setting

$$S^{(2)}(T)=\sum_{x\in L(T)} \delta_{T}(x)^{2}, $$

we have that

3 $$ V(T)=\frac{1}{n} S^{(2)}(T)-\widehat{S}(T)^{2}=\frac{1}{n} S^{(2)}(T)-\frac{1}{n^{2}} S(T)^{2}.   $$

If $T\in \mathcal {T}_{n}$ is a phylogenetic tree, *V*(*T*) is defined as *V*(*π*(*T*)).

### **Example 1**

Let *K*_*n*_ be the comb with *n* leaves. The depths of its leaves are

$$\Delta(K_{n})=\{1,2,3,\ldots,n-2,n-1,n-1\} $$

and therefore

$$\begin{array}{@{}rcl@{}} S(K_{n})=\frac{(n-1)(n+2)}{2},\quad V(K_{n})=\frac{(n-1)(n-2)\left(n^{2}+3n-6\right)}{12n^{2}}. \end{array} $$

### Extremal values of the variance of the leaves’ depths

We have the following theorem for the maximum of *V*, which roughly says that the combs are the most unbalanced multifurcating trees according to *V*.

#### **Theorem 1**

The maximum value of *V* on $\mathcal {T}^{*}_{n}$ is reached exactly at the comb *K*_*n*_.

So, the maximum value of *V* on $\mathcal {T}^{*}_{n}$ is

$$V(K_{n})=\frac{(n-1)(n-2)\left(n^{2}+3n-6\right)}{12n^{2}}. $$

Since *K*_*n*_ is bifurcating, this is also the maximum value of *V* on $\mathcal {BT}^{*}_{n}$.

Theorem 1 is proved by induction on *n*, using a series of lemmas that describe the behaviour of *V* when we remove a deepest leaf from a rooted tree. We provide these lemmas, with their proofs, and the proof of this theorem in Section SN-5 of the Additional file 1.

Let us consider now the minimum *V* value problem. The trees in $\mathcal {T}^{*}_{n}$ with minimum *V* value are very easy to characterize. Indeed, *V* being a variance, its minimum possible value is 0, and it is achieved exactly at the rooted trees with all their leaves at the same depth. Such trees are often called *taxonomic trees*, by analogy with the usual taxonomies with a fixed set of taxonomic ranks, and they include the fully symmetric bifurcating trees. In particular, the rooted star *RS*_*n*_ with *n* leaves, all of them of depth 1, has *V*(*RS*_*n*_)=0. So, the multifurcating case being completely solved, we shall restrict ourselves henceforth to bifurcating trees.

With respect to the bifurcating trees, as we have already mentioned in the “Background” section, we had several reasons to expect that the minimum value of *V* on $\mathcal {BT}^{*}_{n}$ would be achieved at the maximally balanced tree *B*_*n*_ (see Definition 1 in the “Methods” section). These trees were already known to yield the minimum values —among the bifurcating trees with their number of leaves— of the Sackin index [15], the Colless index [10], and the total cophenetic index [31], and the maximum value of the rooted quartets index [11]; for the first two balance indices, this minimum value may also be reached at other trees, while for the last two indices the corresponding extremal value is achieved only at the maximally balanced trees. Recall also that when *n* is a power of 2, *B*_*n*_ is fully symmetric.

As a matter of fact, since the variance of the leaves’ depths is invariant under depth-equivalence, we expected this minimum to be achieved at the trees that are depth-equivalent to maximally balanced trees. We provide in Proposition 5 in the “Methods” section several alternative characterizations of these trees. This proposition offers two more reasons for the educated guess that they should have the minimum variance of the leaves’ depths among the bifurcating trees with their number of leaves. Indeed, on the one hand, it turns out that the trees depth-equivalent to *B*_*n*_ are exactly the trees in $\mathcal {BT}^{*}_{n}$ containing leaves of at most two different depths differing at most by 1 unit, thus being intuitively good candidates for the trees with the least variation in their leaves’ depths. On the other hand, these trees are exactly the bifurcating trees with minimum Sackin index, which we call *of type* *F*_*n*_ (see Definition 2 in the “Methods” section).

It turns out that this educated guess holds for *n* up to 183, but not beyond that. (The minimum value of *V* on every $\mathcal {BT}^{*}_{n}$ with *n*≤2^20^ and the types of trees where they are achieved are available at the GitHub repository https://github.com/biocom-uib/var_depths.) When *n*=184 there is at least one bifurcating tree with smaller *V* value than *B*_184_. Indeed, consider the tree *T* depicted in Fig. 2. It has 174 leaves of depth 8, 9 leaves of depth 7 and one leaf of depth 2 and hence *V*(*T*)≈0.2379, while *V*(*B*_184_)≈0.2382.

So, we establish now two results on the trees $T\in \mathcal {BT}^{*}_{n}$ that achieve the minimum *V* value. On the one hand, the next theorem provides a set of necessary conditions for the trees that yield the minimum *V* value in $\mathcal {BT}^{*}_{n}$, for *n*≥5. The proof, split into a series of lemmas, is given in Section SN-6 of the Additional file 1.

#### **Theorem 2**

If $T\in \mathcal {BT}^{*}_{n}$ has the minimum value of *V*, then it is of some type $T_{n;l_{1},\ldots,l_{j}}$ (see Definition 3 in the “Methods” section) with 5≤*l*_1_<⋯<*l*_*j*_≤*δ*(*T*)−2.

On the other hand, the next theorem states that the maximally balanced trees almost never achieve the minimum *V* value on $\mathcal {BT}^{*}_{n}$. We provide its proof in Section SN-7 of the Additional file 1.

#### **Theorem 3**

As *m* grows to *∞*, the fraction of values *n*∈[ 2,2^*m*^] such that *V*(*B*_*n*_) is minimum on $\mathcal {BT}^{*}_{n}$ tends to 0.

Theorem 2 implies the correctness of Algorithm 1 for the computation of the minimum value of *V* on $\mathcal {BT}^{*}_{n}$ (the equations (10) and (11) used in it are given after Lemma 2 in the “Methods” section). The implementation of this algorithm in R and in Python is also available at the GitHub repository https://github.com/biocom-uib/var_depths.


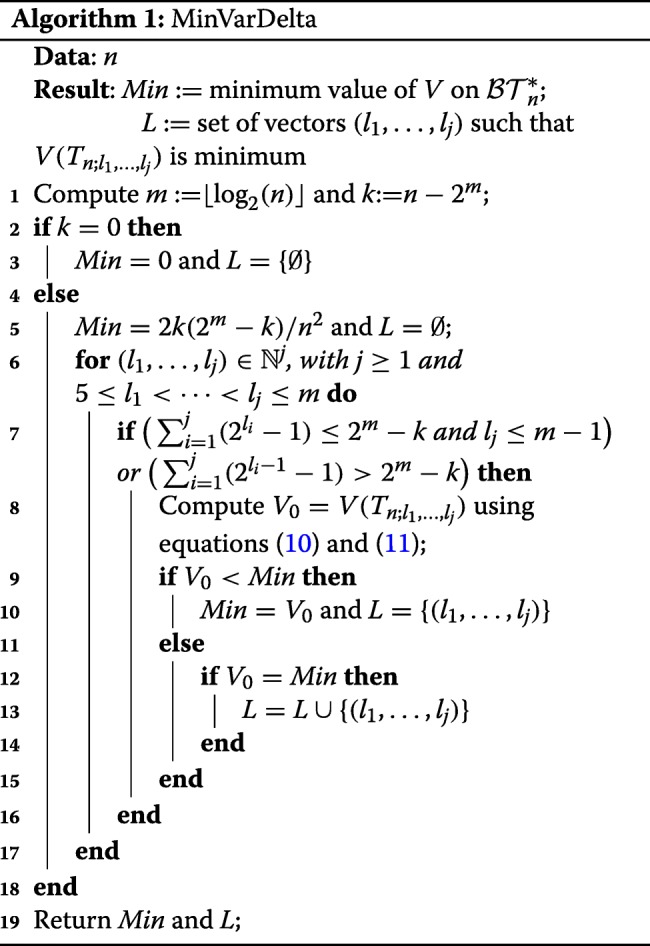


This algorithm runs in time *O*(*n* log(*n*)). Indeed, for every *j*≥1, the set

$$\left\{\left(l_{1},\ldots,l_{j}\right)\in \mathbb{N}^{j}\mid 5\leq l_{1}<\cdots< l_{j}\leq m\right\} $$

has $\binom {m-4}{j}$ elements (where *m*=⌊log2(*n*)⌋) and for each sequence (*l*_1_,…,*l*_*j*_) in this set, Algorithm 1 performs *O*(*j*) operations, and therefore the total number of operations is in

$$O\left(\sum_{j=1}^{m-4}\binom{m-4}{j}j\right)=O\left(2^{m-5}m\right)=O(n\log(n)). $$

### Expected value of *V* under the yule model

Let *V*_*n*_ be the random variable that chooses a phylogenetic tree $T\in \mathcal {BT}_{n}$ and computes *V*(*T*). Its expected value under the Yule model is given by the following result. In it, and henceforth, *H*_*n*_ denotes the *n*-th *harmonic number*, $H_{n}=\sum _{i=1}^{n} 1/i$.

#### **Theorem 4**

For every *n*≥1,

$$E_{Y}(V_{n})=\frac{2(n+1)}{n}\cdot H_{n}+\frac{1}{n}-5. $$

To prove this formula, notice that, by equation (3), if we denote by $S^{(2)}_{n}$ and $S^{2}_{n}$ the random variables that choose a tree $T\in \mathcal {BT}_{n}$ and compute *S*^(2)^(*T*) and *S*(*T*)^2^, respectively, then

4 $$ E_{Y}(V_{n})=\frac{1}{n}E_{Y}\left(S^{(2)}_{n}\right)-\frac{1}{n^{2}}E_{Y}\left(S_{n}^{2}\right).   $$

Now, the expected value $E_{Y}(S_{n}^{2})$ was computed in Theorem 2 in [6]:

5 $$ {}E_{Y}\left(S_{n}^{2}\right)\!= 4n^{2}\left(H_{n}^{2}\,-\,H_{n}^{(2)} \,-\,2H_{n}\right)\! -\! 2nH_{n}+11n^{2}-n   $$

where $H_{n}^{(2)}=\sum _{i=1}^{n} 1/i^{2}$. As to $E_{Y}\left (S^{(2)}_{n}\right)$, its value is given by the next proposition, whose proof we provide in Section SN-8 of the Additional file 1.

#### **Proposition 1**

For every *n*≥1,

$$E_{Y}\left(S^{(2)}_{n}\right)=2n\left(2H_{n}^{2}-3H_{n}-2H_{n}^{(2)}+3\right). $$

Theorem 4 is then deduced from (4), (5), and this proposition as follows:

$$\begin{aligned} E_{Y}(V_{n}) &= \frac{1}{n} E_{Y}\left(S^{(2)}_{n}\right)-\frac{1}{n^{2}}E_{Y}\left(S_{n}^{2}\right)\\[1ex] &= 4H_{n}^{2}-4H_{n}^{(2)}-6H_{n}+6-4H_{n}^{2}+4H_{n}^{(2)}\\ &\quad+8H_{n}+\frac{2}{n}H_{n}-11+\frac{1}{n} \\[1ex] &= \left(2 +\frac{2}{n}\right)H_{n}+\frac{1}{n}-5. \end{aligned} $$

#### **Remark 1**

Using that *H*_*n*_= ln(*n*)+*O*(1)(see, for instance, [20, p. 264]) and that *E*_*Y*_(*S*_*n*_)=2*n*(*H*_*n*_−1) [27, Appendix], we have that

$$\begin{array}{l} E_{Y}\left(\widehat{S}_{n}\right)=2H_{n}-2\sim 2\ln(n)\\[1ex] E_{Y}(V_{n})=\frac{2(n+1)}{n}H_{n}+\frac{1}{n}-5\sim 2\ln(n) \end{array} $$

and therefore the expected values under the Yule model of both the mean and the variance of the leaves’ depths of a bifurcating rooted tree grow asymptotically as 2 ln(*n*).

### Expected value of *V* under the uniform model

The expected value under the uniform model of the random variable *V*_*n*_ is given by the following result.

#### **Theorem 5**

For every *n*≥1,

$$E_{U}(V_{n}) = \frac{(2n-1)(n-1)}{3n}-\frac{n-1}{2n}\cdot \frac{(2n-2)!!}{(2n-3)!!}. $$

To obtain this formula, we shall use the following identity, which is implied again by Eq. (3):

6 $$ E_{U}(V_{n})=\frac{1}{n}E_{U}\left(S^{(2)}_{n}\right)-\frac{1}{n^{2}}E_{U}\left(S_{n}^{2}\right).   $$

Now, $E_{U}\left (S_{n}^{(2)}\right)$ and $E_{U}\left (S_{n}^{2}\right)$ satisfy the following recurrences, which we shall solve using Proposition 6.

#### **Proposition 2**

For every *n*≥2,

$$ E_{U}\left(S_{n}^{(2)}\right) = 2\sum_{k=1}^{n-1} C_{k,n-k} E_{U} \left(S_{k}^{(2)}\right)+2n\cdot \frac{(2n-2)!!}{(2n-3)!!}-3n. $$

#### **Proposition 3**

For every *n*≥2,

$$\begin{array}{@{}rcl@{}} E_{U}\left(S_{n}^{2}\right)&=& 2\sum_{k=1}^{n-1} C_{k,n-k} E_{U}\left(S_{k}^{2}\right)\\&&+ \frac{5n^{2}}{2}\cdot \frac{(2n-2)!!!}{(2n-3)!!}-n(5n-2). \end{array} $$

The proofs of these propositions are provided in Sections SN-9 and SN-10 of the Additional file 1, respectively.

#### **Proposition 4**

For every *n*≥1,

$$\begin{array}{*{20}l} & E_{U}\left(S^{(2)}_{n}\right)=(4n-1)n-3n\cdot\frac{(2n-2)!!}{(2n-3)!!}\\ & E_{U}\left(S_{n}^{2}\right) = \frac{n(10n^{2}-1)}{3} - \frac {n(5n+1)}{2}\cdot \frac{(2n-2)!!}{(2n-3)!!} \end{array} $$

#### *Proof*

By Proposition 2, the sequence $E_{U}\left (S_{n}^{(2)}\right)$ is the solution of the recurrence

$$X_{n} = 2\sum_{k=1}^{n-1} C_{k,n-k} X_{k} -3n +2n\cdot \frac{(2n-2)!!}{(2n-3)!!} $$

with initial condition $X_{1}=E_{U}\left (S_{1}^{(2)}\right)=0$. By Proposition 6, this solution is

$$\begin{array}{@{}rcl@{}} E_{U}\left(S_{n}^{(2)}\right) &=&8\binom{n}{2}+3n-3n\cdot \frac{(2n-2)!!}{(2n-3)!!} \\&=&4n^{2}-n-3n\cdot \frac{(2n-2)!!}{(2n-3)!!}. \end{array} $$

As for the sequence $E_{U}\left (S_{n}^{2}\right)$, by Proposition 3 it is the solution of the recurrence

$$\begin{array}{@{}rcl@{}} X_{n}&=&2\sum_{k=1}^{n-1} C_{k,n-k}X_{k}-10\binom{n}{2}\\&&-3n+\left(5\binom{n}{2}+\frac{5}{2}n\right) \frac{(2n-2)!!}{(2n-3)!!} \end{array} $$

with initial condition $X_{1}=E_{U}\left (S_{1}^{2}\right)=0$. By Proposition 6, this solution is

$$\begin{array}{@{}rcl@{}} E_{U}\left(S_{n}^{2}\right) & =&20\binom{n}{3}+20\binom{n}{2}+3n-\left(5\binom{n}{2}+3n\right)\frac{(2n-2)!!}{(2n-3)!!}\\ & =&\frac{10n^{3}-n}{3}-\frac{5n^{2}+n}{2}\cdot \frac{(2n-2)!!}{(2n-3)!!}. \end{array} $$

□

Combining identity (6) with Proposition 4, we finally obtain the closed formula for *E*_*U*_(*V*_*n*_) given in Theorem 5.

#### **Remark 2**

Using Stirling’s approximation for large factorials we have that

7 $$\begin{array}{@{}rcl@{}} \frac{(2n-2)!!}{(2n-3)!!} & =& \frac{\left(2^{n-1}\cdot (n-1)!\right)^{2}}{(2n-2)!}\\ & \sim &\frac{\left(2^{n-1}\sqrt{2\pi (n-1)} (n-1)^{n-1}e^{-(n-1)}\right)^{2}} {\sqrt{2\pi (2n-2)}(2n-2)^{2n-2}e^{-(2n-2)}}\sim \sqrt{\pi n} \end{array} $$

Then, using the following expression for *E*_*U*_(*S*_*n*_) established in [31, Thm. 22]

8 $$ E_{U}(S_{n}) =n\left(\frac{(2n-2)!!}{(2n-3)!!}-1\right),   $$

we have that

$$\begin{array}{*{20}l} E_{U}\left(\widehat{S}_{n}\right) & \,=\, \frac{(2n-2)!!}{(2n-3)!!}-1\sim \sqrt{\pi n}\\ E_{U}(V_{n}) & \,=\,\frac{(2n-1)(n-1)}{3n}-\frac{n-1}{2n}\cdot \frac{(2n-2)!!}{(2n-3)!!}\sim \frac{2}{3}n \end{array} $$

So, against what happened with the Yule model (see Remark 1), the expected values under the uniform model of the mean depth and the variance of the depths have different asymptotic orders.

### Bonus results

As a by-product of our computations, we have been able to obtain also closed formulas for the variance of the Sackin index and the total cophenetic index under the uniform model as well as of their covariance.

#### **Theorem 6**

Let *S*_*n*_ and *Φ*_*n*_ be, respectively, the random variables that take a phylogenetic tree $T\in \mathcal {BT}_{n}$ and compute its Sackin index *S*(*T*) and its total cophenetic index *Φ*(*T*). Then, for every *n*≥1,

1. The variance of *S*_*n*_ under the uniform model is
  $$\begin{array}{@{}rcl@{}} \sigma_{U}^{2}(S_{n})&=& \frac{n(10n^{2}-3n-1)}{3}\\&&-\binom{n+1}{2}\cdot \frac{(2n-2)!!}{(2n-3)!!} \\&&-n^{2}\left(\frac{(2n-2)!!}{(2n-3)!!}\right)^{2} \end{array} $$
2. The variance of *Φ*_*n*_ under the uniform model is
  $$\begin{array}{@{}rcl@{}} \sigma_{U}^{2}(\Phi_{n}) & =& \binom{n}{2}\frac{(2 n - 1) (7n^{2} - 3n - 2)}{30}\\&&-\binom{n}{2}\frac{5n^{2}-n-2}{32}\cdot \frac{(2n-2)!!}{(2n-3)!!}\\ &&- \frac{1}{4} \binom{n}{2}^{2} \left(\frac{(2n-2)!!}{(2n-3)!!}\right)^{2} \end{array} $$
3. The covariance of *S*_*n*_ and *Φ*_*n*_ under the uniform model is
  $${\begin{aligned} Cov_{U}(S_{n},\Phi_{n}) & =\binom{n}{2}\frac{26n^{2}-5n-4}{15}-\frac{3n+2}{8}\binom{n}{2} \frac{(2n-2)!!}{(2n-3)!!}\\ &\qquad-\frac{n}{2}\binom{n}{2}\left(\frac{(2n-2)!!}{(2n-3)!!}\right)^{2} \end{aligned}} $$

The value of $\sigma _{U}^{2}(S_{n})$ can be obtained directly from Proposition 4 and the expression for *E*_*U*_(*S*_*n*_) recalled in (8) as follows:

$$\begin{array}{@{}rcl@{}} \sigma_{U}^{2}(S_{n})& = &E_{U}\left(S_{n}^{2}\right)-E_{U}(S_{n})^{2}\\ & =&\frac{10}{3} n^{3} -\frac{1}{3}n- \frac {n(5n+1)}{2}\frac{(2n-2)!!}{(2n-3)!!}-n^{2}\left(\frac{(2n-2)!!}{(2n-3)!!}-1\right)^{2}\\ & =& \frac{10}{3}n^{3}-\frac{1}{3}n-n^{2}-n^{2}\left(\frac{(2n-2)!!}{(2n-3)!!}\right)^{2}-\frac{n(n+1)}{2}\cdot \frac{(2n-2)!!}{(2n-3)!!}. \end{array} $$

The proofs of (2) and (3) are longer, and they consist in finding recurrences for $\sigma _{U}^{2}(\Phi _{n})$ and *Cov*_*U*_(*S*_*n*_,*Φ*_*n*_) of the same type as those given in Propositions 2 or 3 and then solving them using Proposition 6. We give these proofs in Sections SN-11 and SN-12 of the Additional file 1, respectively.

Using the limit behaviour $(2n-2)!!/(2n-3)!!\sim \sqrt {\pi n}$ (see (7)) it is straightforward to check that the formula for $\sigma ^{2}_{U}(S_{n})$ given in the last theorem is in agreement with its asymptotic behaviour established in [4, Rem. 3]. Indeed,

9 $$\begin{array}{@{}rcl@{}} \sigma_{U}^{2}(S_{n})\sim \frac{n\left(10n^{2}-3n-1\right)}{3}-\binom{n+1}{2}\sqrt{\pi n}-n^{2}\pi n\sim \frac{10-3\pi}{3}\cdot n^{3}.  \end{array} $$

For the asymptotic behaviour of $\sigma _{U}^{2}(\Phi _{n})$ and $Cov_{U}^{2}(S_{n},\Phi _{n})$, similar computations show that

$$\sigma_{U}^{2}(\Phi_{n})\!\sim\! \frac{56-15\pi}{240}\cdot n^{5},\ Cov_{U}(\Phi_{n},S_{n}) \!\sim\! \frac{52-15\pi}{60}\cdot n^{4}. $$

From these expressions we obtain that the limit behaviour of Pearson’s correlation coefficient of *S*_*n*_ and *Φ*_*n*_ under the uniform model is

$$\rho_{U}(S_{n},\Phi_{n})\sim \frac{\frac{52-15\pi}{60}}{\sqrt{\frac{10-3\pi}{3}\cdot\frac{56-15\pi}{240}}}\approx 0.965. $$

It should be mentioned that, under the Yule model, the limit of Pearson’s correlation coefficient of *S*_*n*_ and *Φ*_*n*_ is around 0.89 [6].

To double-check our formulas, we have computed the values of $E_{Y}(V_{n}), E_{U}(V_{n}), \sigma _{U}^{2}(S_{n}), \sigma _{U}^{2}(\Phi _{n})$, and *Cov*_*U*_(*S*_*n*_,*Φ*_*n*_), for *n*=3,…,8, from the values of *V*, *S* and *Φ* on all trees in the corresponding $\mathcal {BT}_{n}$, and they agree with the figures given by our formulas: these values are given in Table 1. The R script used in these computations is available on the GitHub repository https://github.com/biocom-uib/var_depths.

**Table 1:** $E_{Y}(V_{n}), E_{U}(V_{n}), \sigma _{U}^{2}(S_{n}), \sigma _{U}^{2}(\Phi _{n})$, and *Cov*_*U*_(*S*_*n*_,*Φ*_*n*_) for *n*=3,…,8

*n*	3	4	5	6	7	8
*E*_*Y*_(*V*_*n*_)	0.2222	0.4583	0.6800	0.8833	1.0694	1.2402
*E*_*U*_(*V*_*n*_)	0.2222	0.5500	0.9371	1.3624	1.8145	2.2864
$\sigma _{U}^{2}(S_{n})$	0	0.1600	0.7755	2.2358	4.9991	9.5765
$\sigma _{U}^{2}(\Phi _{n})$	0	0.6400	4.7755	19.5828	58.9752	146.2314
*Cov*_*U*_(*S*_*n*_,*Φ*_*n*_)	0	0.3200	1.9184	6.5805	17.0441	37.0899

## Discussion

**Fig. 4:**
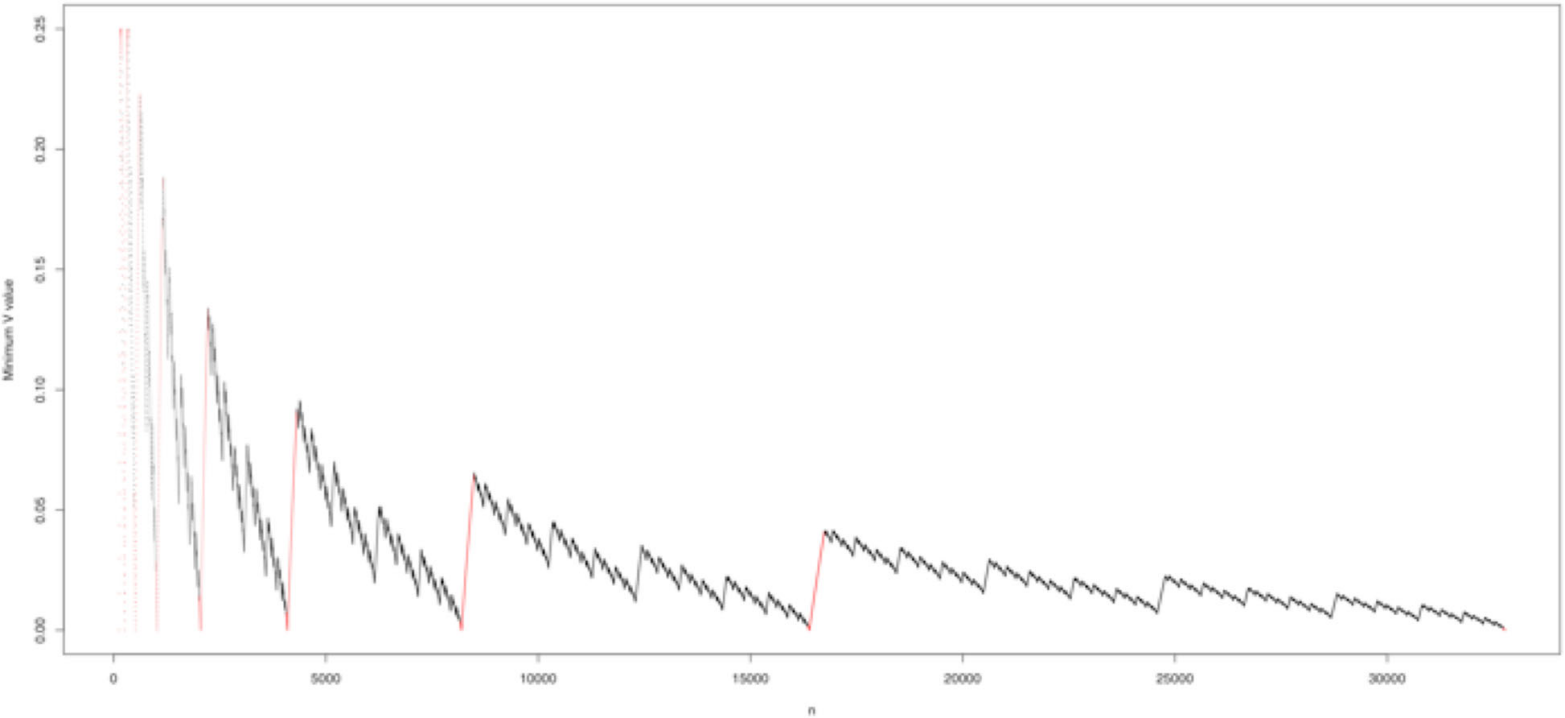
Scatter plot of the minimum *V* values on $\mathcal {BT}^{*}_{n}$, for *n* between 2^7^ and 2^15^. The values of *n* for which the minimum of *V* is achieved at *B*_*n*_ are drawn in red

When the number *n* of leaves is smaller than 184, *V* classifies the maximally balanced trees *B*_*n*_ (as well as those trees depth-equivalent to them) as the most balanced bifurcating rooted trees with *n* leaves. But this last property fails for almost all numbers *n* of leaves larger than 184. So, we have provided a quasilinear time algorithm that produces, for any given *n*, the minimum *V* value on the space $\mathcal {BT}^{*}_{n}$ of bifurcating rooted trees with *n* leaves and the type $T_{n;l_{1},\ldots,l_{j}}$ of trees achieving it. We have run our algorithm for every *n* up to 2^20^. The results are available at the GitHub repository https://github.com/biocom-uib/var_depths companion to this paper. Figure 4 depicts the minimum *V* values for *n*=2^7^ to 2^15^. In this scatter plot, the red dots correspond to values of *n* for which *V*(*B*_*n*_) is minimum.

**Fig. 5:**
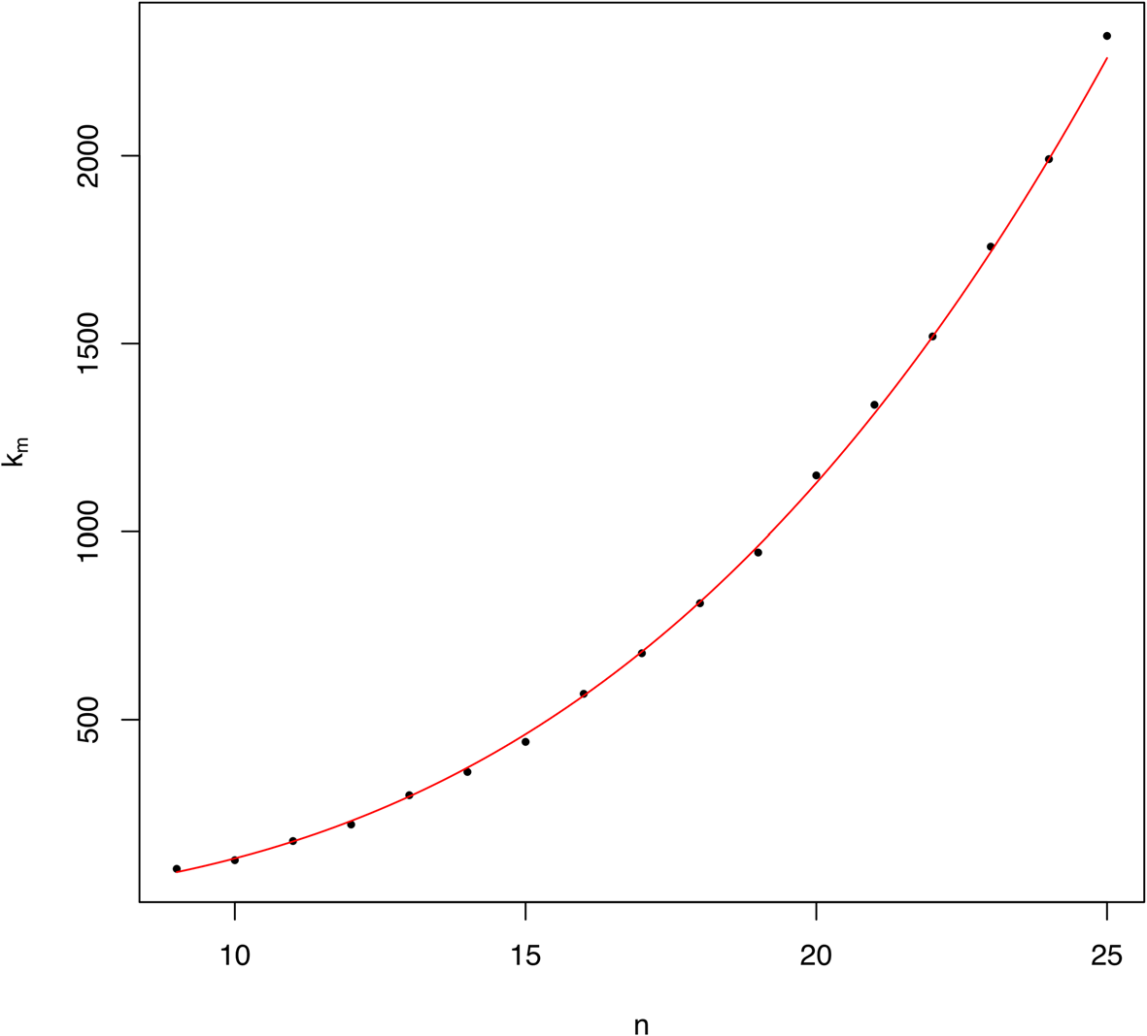
Scatter plot of the points (*m*,*k*_*m*_), for *m*=9,…,25. In red, the curve *y*=0.1042*x*^3.1^, which gives the best fit of *k*_*m*_ as a function of *m* (*R*^2^=0.9987)
- For all tested *n*, if *l*_1_,…,*l*_*j*_ are such that (with the notations *m*=⌊log2(*n*)⌋ and *k*=*n*−2^*m*^ used throughout this paper) *j*≥1 and $k< 2^{m}-\sum _{i=1}^{j} \left (2^{l_{i}}-1\right)$, and if $T_{n;l_{1},\ldots,l_{j}}$ has minimum *V* value on $\mathcal {BT}^{*}_{n}$, then $T_{n+1;l_{1},\ldots,l_{j}}$ has also minimum *V* value on $\mathcal {BT}^{*}_{n+1}$. Again, we have not been able to prove this fact, but we conjecture that it also holds for every *n*. We should mention here that when *j*=0, we have that $k< 2^{m}-\sum _{i=1}^{j} \left (2^{l_{i}}-1\right)$, but it may happen that *T*_*n*;−_ has minimum *V* value on $\mathcal {BT}^{*}_{n}$ and *T*_*n*+1;−_ does not on $\mathcal {BT}^{*}_{n+1}$. So, the premise *j*≥1 is necessary for the implication to be valid.
- In relation to this last point, for all tested intervals [2^*m*^,2^*m*+1^), the sequence formed by the lengths of segments of consecutive numbers *n* of leaves such that the trees $T_{n;l_{1},\ldots,l_{j}}$ achieving the minimum *V* value on $\mathcal {BT}^{*}_{n}$ have the same *l*_1_,…,*l*_*j*_ values presents some intriguing regularities. Take for instance the sequence corresponding to *m*=12, presented in Table 2 in reversed order. The figures in this table mean that, when *n* descends from 2^13^−1 to 2^12^, for the first 29 values the trees $T_{n;l_{1},\ldots,l_{j}}$ achieving the minimum *V* value on $\mathcal {BT}^{*}_{n}$ have the same *l*_1_,…,*l*_*j*_ (actually, as we have mentioned above, they have *j*=0); then, the same happens with the next 2 values of *n*; then, the same happens with the next 25 values of *n*; and so on. As we can see, the sequence ends in 52 each two lines, in 88 each four lines, and in 132 each eight lines. And, as *m* increases, the different sequences associated with it present the same pattern with practically the same numbers: see, for instance, the sequence associated to *m*=13, presented in Table 3.

The computations carried out show that the trees with minimum *V* value present several curious regularities. For instance, Fig. 4 seems to hint a fractal structure in the sequence of minimum values of *V*, as well as a tendency to decrease with *n*. Let us mention some other such observed regularities, of which we have only been able to prove one, leaving the verification of the rest as open problems:

- For all tested *n*, the minimum value of *V* on $\mathcal {BT}^{*}_{n}$ is achieved at only one type of trees $T_{n;l_{1},\ldots,l_{j}}$, and hence the tree in $\mathcal {BT}^{*}_{n}$ with minimum *V* value is unique up to depth-equivalence. We have not been able to prove this uniqueness in general, but we conjecture that it holds for every *n*.
- For all tested *n*≥2^8^, the values of *n* for which the minimum value of *V* on $\mathcal {BT}^{*}_{n}$ is reached at the maximally balanced tree form small intervals around 2^*m*^ of the form [2^*m*^−*x*_0_,2^*m*^+*k*_*m*_] (see the red patches in Fig. 4).
  We have been able to prove that, in the left-hand side end of these intervals, *x*_0_ is always 29. More specifically, if *n*∈[2^*m*^−29,2^*m*^], the minimum value of *V* on $\mathcal {BT}^{*}_{n}$ is always reached at *B*_*n*_, but when *n*=2^*m*^−30 and *m*≥9, the minimum value of *V* on $\mathcal {BT}^{*}_{n}$ is always reached exactly at the trees of type *T*_*n*;6_, which are not of type *F*_*n*_. We provide a proof of this property in Section SN-13 of the Additional file 1.
  As far as the right-hand side end 2^*m*^+*k*_*m*_ of these intervals goes, for the range of values of *m* that we have tested we have obtained that *k*_*m*_∼0.1015*m*^3.11^; see Fig. 5. Since in the proof of Theorem 3 (see Section SN-7 of the Additional file 1) we have obtained, for large enough *m*, an upper bound $\sim \frac {4}{9}m^{3}$ for *k*_*m*_, we conjecture that *k*_*m*_ is actually in *Θ*(*m*^3^).

**Table 2:** Sequence, in reverse order, of the numbers of consecutive values *n*∈[2^12^,2^13^) such that the trees $T_{n;l_{1},\ldots,l_{j}}$ achieving the minimum *V* value on $\mathcal {BT}^{*}_{n}$ have the same *l*_1_,…,*l*_*j*_

29	2	25	12	29	2	18	28	29	2	25	12	29	2	7	52
29	2	25	12	29	2	18	28	29	2	25	12	21	88		
29	2	25	12	29	2	18	28	29	2	25	12	29	2	7	52
29	2	25	12	29	2	18	28	29	2	25	12	132			
29	2	25	12	29	2	18	28	29	2	25	12	29	2	7	52
29	2	25	12	29	2	18	28	29	2	25	12	21	88		
29	2	25	12	29	2	18	28	29	2	25	12	29	2	7	52
29	2	25	12	29	2	18	28	27	206						
29	2	25	12	29	2	18	28	29	2	25	12	29	2	7	52
29	2	25	12	29	2	18	28	29	2	25	12	21	88		
29	2	25	12	29	2	18	28	29	2	25	12	29	2	7	52
29	2	25	12	29	2	18	28	29	2	25	12	132			
29	2	25	12	29	2	18	28	29	2	25	12	29	2	7	52
29	2	25	12	29	2	18	28	29	2	25	12	21	88		
29	2	25	12	29	2	18	28	29	2	25	12	29	2	7	52
29	2	7	442

**Table 3:** Sequence, in reverse order, of the numbers of consecutive values *n*∈[2^13^,2^14^) such that the trees $T_{n;l_{1},\ldots,l_{j}}$ achieving the minimum *V* value on $\mathcal {BT}^{*}_{n}$ have the same *l*_1_,…,*l*_*j*_

29	2	25	12	29	2	18	28	29	2	25	12	29	2	7	52
29	2	25	12	29	2	18	28	29	2	25	12	21	88		
29	2	25	12	29	2	18	28	29	2	25	12	29	2	7	52
29	2	25	12	29	2	18	28	29	2	25	12	1	130		
29	2	25	12	29	2	18	28	29	2	25	12	29	2	7	52
29	2	25	12	29	2	18	28	29	2	25	12	21	88		
29	2	25	12	29	2	18	28	29	2	25	12	29	2	7	52
29	2	25	12	29	2	18	28	28	204						
29	2	25	12	29	2	18	28	29	2	25	12	29	2	7	52
29	2	25	12	29	2	18	28	29	2	25	12	21	88		
29	2	25	12	29	2	18	28	29	2	25	12	29	2	7	52
29	2	25	12	29	2	18	28	29	2	25	12	1	130		
29	2	25	12	29	2	18	28	29	2	25	12	29	2	7	52
29	2	25	12	29	2	18	28	29	2	25	12	21	88		
29	2	25	12	29	2	18	28	29	2	25	12	29	2	7	52
29	2	25	12	29	2	13	302								
29	2	25	12	29	2	18	28	29	2	25	12	29	2	7	52
29	2	25	12	29	2	18	28	29	2	25	12	21	88		
29	2	25	12	29	2	18	28	29	2	25	12	29	2	7	52
29	2	25	12	29	2	18	28	29	2	25	12	1	130		
29	2	25	12	29	2	18	28	29	2	25	12	29	2	7	52
29	2	25	12	29	2	18	28	29	2	25	12	21	88		
29	2	25	12	29	2	18	28	29	2	25	12	29	2	7	52
29	2	25	12	29	2	18	28	28	204						
29	2	25	12	29	2	18	28	29	2	25	12	29	2	7	52
29	2	25	12	29	2	18	28	29	2	25	12	21	88		
29	2	25	12	29	2	18	28	29	2	25	12	29	2	7	52
29	2	25	12	29	2	18	28	29	2	25	12	1	130		
29	2	25	12	29	2	18	28	29	2	25	12	29	2	7	52
29	2	25	12	29	2	18	28	29	2	25	12	21	88		
29	2	25	12	29	2	18	28	29	2	25	12	26	598

## Conclusion

In his seminal paper on the shape of phylogenetic trees [39], Sackin postulated the existence of a direct association between the degree of imbalance of a rooted bifurcating tree and the variation of its leaves’ depths. This led several authors to use the variance *V*(*T*) of the depths of the leaves of a phylogenetic tree *T* as a measure of its imbalance [25–27]. But this shape index based on Sackin’s original proposal seems not to have prospered and the phylogenetics community favoured instead what we call nowadays the *Sackin index* *S*(*T*), the sum of the depths of the leaves of *T*, which, despite its name, was actually introduced by Sokal [41, 42]. In this paper we have investigated some properties of *V* as a balance index: the trees on which it achieves its extremal values for any given number of leaves and its expected value under the Yule and the uniform models for bifurcating phylogenetic trees.

With respect to the extremal values of *V*, its maximum value on the space $\mathcal {BT}^{*}_{n}$ of bifurcating trees with *n* leaves is always reached at the comb, and when the number of leaves *n* is smaller than 184, its minimum value on $\mathcal {BT}^{*}_{n}$ is achieved at the maximally balanced tree (together with those trees depth-equivalent to it). But from *n*=184 on, this last property fails for almost all numbers *n* of leaves. Since the maximally balanced trees were considered “the most balanced trees” by Shao and Sokal [41] and they are classified (sometimes tied with other trees) as most balanced by many other balance indices, including the Sackin index *S*, this hints that *V* is not suitable as a balance index for bifurcating phylogenetic trees with large numbers of leaves, although it can still be of interest as a shape index.

We have then provided a quasilinear algorithm that produces, for every *n*, the minimum *V* value on the space $\mathcal {BT}^{*}_{n}$ of rooted bifurcating trees with *n* leaves and the trees achieving it. This algorithm simply searches for these minimal trees in a suitably small set of candidate tree types $T_{n;l_{1},\ldots,l_{j}}$ (with *j*≥0 and 5≤*l*_1_<⋯<*l*_*j*_≤⌊log2(*n*)⌋), defined as those bifurcating trees containing leaves only of maximal depth *δ* and submaximal depth, *δ*−1, plus a single leaf of each depth *δ*−*l*_*i*_, for *i*=1,…,*j*. The trees depth-equivalent to maximally balanced trees are exactly those of type *T*_*n*;−_. Implementations both in R and Python of this algorithm are available at the GitHub repository https://github.com/biocom-uib/var_depths. Unfortunately, we have not been able to find a closed formula that, given *n*, gives the type of trees $T_{n;l_{1},\ldots,l_{j}}$ in $\mathcal {BT}^{*}_{n}$ with minimum *V* value or even such minimum value, without resorting to a search procedure.

The second main contribution of this paper are the closed formulas for the expected value of *V* under the Yule and the uniform models, as well as for the variance under the uniform model of the Sackin index *S* and the total cophenetic index *Φ*, and for their covariance (Theorems 4 to 6). These formulas are explicit and hold on spaces $\mathcal {BT}_{n}$ of bifurcating phylogenetic trees with any number *n* of leaves, and therefore they can be meaningfully used in tests involving trees of any size. Additionally, the formulas for the variances of *S* and *Φ* can be used to properly *standardize* them in each $\mathcal {BT}_{n}$ relative to the uniform model. Recall that the *standardization* of a shape index relative to a probabilistic model of phylogenetic trees is performed, in principle, by subtracting to the index its expected value and dividing the result by its standard deviation. But, for instance, because it lacks of a closed formula for *σ*_*U*_(*S*_*n*_), the current version of the R package apTreeshape standardizes the Sackin index relative to the uniform model by dividing by the square root of the asymptotic approximation (9) of $\sigma ^{2}_{U}(S_{n})$ [5].

## Methods

### Trees depth-equivalent to maximally balanced trees

An internal node *v* of a bifurcating tree *T* is said to be *balanced* when the numbers of descendant leaves of its two children are as similar as possible: equal if *v* has an even number of descendant leaves, and differing by 1 if the number of descendant leaves of *v* is odd. In other words, a node *v* with *k* descendant leaves is balanced if its two children have ⌊*k*/2⌋ and ⌈*k*/2⌉ descendant leaves, respectively.

#### **Definition 1**

A bifurcating tree *T* is *maximally balanced* when all its internal nodes are balanced.

**Fig. 6:**
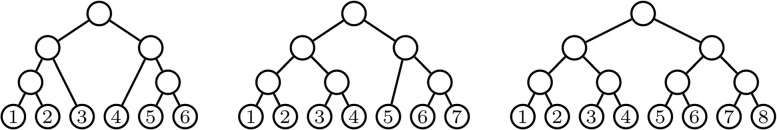
Three maximally balanced trees. The tree with 8 leaves is fully symmetric

Recurrently, a bifurcating tree is maximally balanced when its root is balanced and both subtrees rooted at the children of the root are maximally balanced. This easily implies that, for every number *n* of nodes, there is only one maximally balanced tree with *n* leaves. Indeed, in a maximally balanced tree, the numbers of leaves of the subtrees rooted at the children of the root are fixed, because the root is balanced, and then, since these subtrees are maximally balanced, by recurrence they are unique. We shall denote by *B*_*n*_ the maximally balanced bifurcating tree with *n* leaves. Figure 6 depicts the trees *B*_*n*_ with *n*=6,7,8 leaves. Notice that *B*_8_ is *fully symmetric*: for each internal node, the pair of subtrees rooted at its children are isomorphic. In fact, it is straightforward to prove by induction that, for every $m\in \mathbb {N}$, the maximally balanced tree $\phantom {\dot {i}\!}B_{2^{m}}$ is the fully symmetric bifurcating tree with 2^*m*^ leaves, all of them of depth *m*.

We provide in Proposition 5 below several alternative characterizations of the trees that are depth-equivalent to maximally balanced trees. One of these characterizations says that they are exactly the bifurcating trees with minimum Sackin index, characterized recently by M. Fischer in [15]. Let us recall Fischer’s construction of her minimal Sackin trees.

#### **Definition 2**

**Fig. 7:**
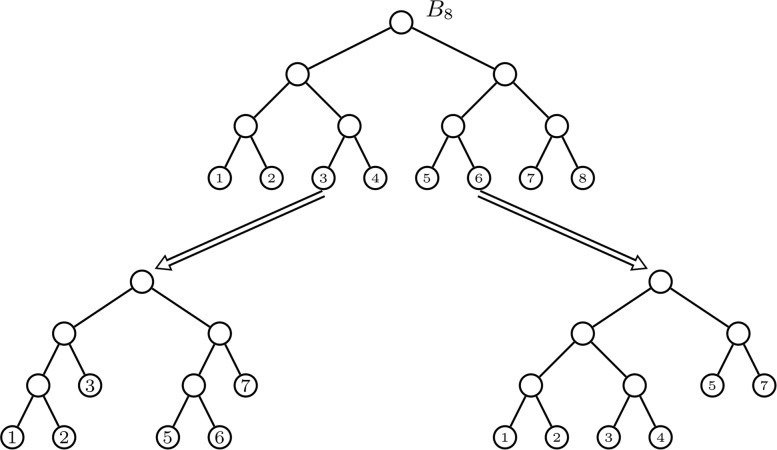
The only two trees in $\mathcal {BT}^{*}_{6}$ of type *F*_6_ as obtained from the fully symmetric tree *B*_8_ by removing 2 cherries: in the left-hand side tree, the cherries (3,4) and (7,8) are replaced by single leaves 3 and 7, respectively, while in the right-hand side tree the cherries replaced by single leaves are (5,6) and (7,8). The left-hand side tree is the maximally balanced tree *B*_6_, the other is only depth-equivalent to it

For every *n*=2^*m*^+*k*, with *m*=⌊log2(*n*)⌋ and 0≤*k*<2^*m*^, a tree $T\in \mathcal {BT}^{*}_{n}$ is of *type* *F*_*n*_ when it is obtained from the fully symmetric bifurcating tree $\phantom {\dot {i}\!}B_{2^{m+1}}$ by removing from it 2^*m*^−*k* cherries and replacing them by their roots, which become leaves of depth *m*; cf. Fig 7.

Equivalently, each tree $T\in \mathcal {BT}^{*}_{n}$ of type *F*_*n*_, with *n*=2^*m*^+*k* and 0≤*k*<2^*m*^, is obtained from the fully symmetric bifurcating tree $\phantom {\dot {i}\!}B_{2^{m}}$ by replacing *k* leaves in it (of depth *m*) by cherries.

#### **Remark 3**

By construction, any tree of type $\phantom {\dot {i}\!}F_{2^{m}+k}$ has 2*k* leaves of depth *m*+1 and the remaining 2^*m*^−*k* leaves of depth *m*. Therefore, all trees of type *F*_*n*_, for any given *n*, are depth-equivalent, and in particular they all have the same *V* value, which can be easily seen to be

$$V\left(F_{2^{m}+k}\right) =\frac{2k(2^{m}-k)}{(2^{m}+k)^{2}}. $$

Notice also that if *n* is a power of 2, then the only tree of type *F*_*n*_ is the corresponding fully symmetric tree *B*_*n*_.

#### **Proposition 5**

For every $T\in \mathcal {BT}^{*}_{n}$, the following conditions are equivalent:

1. *T* is of type *F*_*n*_.
2. There exists a $d_{0}\in \mathbb {N}$ such that *δ*_*T*_(*x*)∈{*d*_0_,*d*_0_+1} for every *x*∈*L*(*T*).
3. $|\delta _{T}(x)-\widehat {S}(T)|<1$ for every *x*∈*L*(*T*).
4. *T* is depth-equivalent to *B*_*n*_.

The proof of this proposition is given in Section SN-2 in the Additional file 1.

### A family of trees that generalize those depth-equivalent to maximally balanced

In our study of the bifurcating trees achieving the minimum *V* value we have encountered the family of trees introduced in the next definition.

#### **Definition 3**

A tree $T\in \mathcal {BT}^{*}_{n}$*is of type*$T_{n;l_{1},\ldots,l_{j}}$, with *j*≥0 and 2≤*l*_1_<⋯<*l*_*j*_≤*δ*(*T*)−2, when it has a single leaf of depth *δ*(*T*)−*l*_*i*_, for each *i*=1,…,*j*, and the rest of its leaves have depths *δ*(*T*) or *δ*(*T*)−1.

Let us emphasize the fact that this definition implies that if *T* is of type $T_{n;l_{1},\ldots,l_{j}}$ with *j*≥1, then *δ*(*T*)≥4 and therefore that if *n*≥4, the trees of type $T_{n;l_{1},\ldots,l_{j}}$ do not have leaves of depth 1. Also notice that the trees of type *F*_*n*_ are those of type *T*_*n*;−_ (i.e., of type $T_{n;l_{1},\ldots,l_{j}}$ with *j*=0), because by Proposition 5, a tree *T* is of type *F*_*n*_ if, and only if, all its leaves have depths *δ*(*T*) or *δ*(*T*)−1.

For every tree $T\in \mathcal {BT}^{*}_{n}$, we shall denote henceforth its numbers of leaves of depths *δ*(*T*) and *δ*(*T*)−1 by *p*_0_(*T*) and *p*_1_(*T*), respectively. The next lemma gives the value of *p*_1_(*T*) in a tree *T* of type $T_{n;l_{1},\ldots,l_{j}}$ as a function of *n*,*l*_1_,…,*l*_*j*_; we provide its proof in Section SN-3 in the Additional file 1. Since if *T* is of type $T_{n;l_{1},\ldots,l_{j}}$, then *p*_0_(*T*)+*p*_1_(*T*)+*j*=*n*, this lemma implies that the multiset of depths of a tree of type $T_{n;l_{1},\ldots,l_{j}}$ depends only on *n*,*l*_1_,…,*l*_*j*_ and therefore that these types of trees are depth-equivalence classes.

#### **Lemma 1**

Let *n*=2^*m*^+*k* with *m*=⌊log2(*n*)⌋ and *k*=*n*−2^*m*^. For every tree *T* of type $T_{n;l_{1},\ldots,l_{j}}$, with *j*≥0 and 2≤*l*_1_<⋯<*l*_*j*_≤*δ*(*T*)−2:

1. If $k+\sum _{i=1}^{j} \left (2^{l_{i}}-1\right)=0$, then *p*_1_(*T*)=0 and the tree is fully symmetric.
2. If $0< k+\sum _{i=1}^{j} \left (2^{l_{i}}-1\right)\leq 2^{m}$, then $p_{1}(T)=2^{m}-k-\sum _{i=1}^{j} \left (2^{l_{i}}-1\right)$ and *δ*(*T*)=*m*+1.
3. If $k+\frac {1}{2}\sum _{i=1}^{j} \left (2^{l_{i}}-2\right)> 2^{m}$, then $p_{1}(T)=3\cdot 2^{m}-k-\sum _{i=1}^{j} \left (2^{l_{i}}-1\right)$ and *δ*(*T*)=*m*+2.
4. If $k+\frac {1}{2}\sum _{i=1}^{j} \left (2^{l_{i}}-2\right)\leq 2^{m}< k+\sum _{i=1}^{j} \left (2^{l_{i}}-1\right)$, then there does not exist any tree *T* of type $T_{n;l_{1},\ldots,l_{j}}$.

The *V* value of a tree of type $T_{n;l_{1},\ldots,l_{j}}$ is given by the following formula, whose easy proof we also give in Section SN-3 in the Additional file 1.

#### **Lemma 2**

If *T* is a tree of type $T_{n;l_{1},\ldots,l_{j}}$, then

$$\begin{array}{@{}rcl@{}} V(T)\,=\,\frac{1}{n^{2}}\left(n\left(p_{1}(T)+\sum_{i=1}^{j} l_{i}^{2}\right)\,-\, \left(p_{1}(T)+\sum_{i=1}^{j} l_{i}\right)^{2}\right). \end{array} $$

Combining the last two lemmas, we obtain that, if *n*=2^*m*^+*k* with *m*=⌊log2(*n*)⌋, then, for every tree *T* of type $T_{n;l_{1},\ldots,l_{j}}$:

- If $\sum _{i=1}^{j} \left (2^{l_{i}}-1\right)\leq 2^{m}-k$
  10 $$ {\begin{aligned} V(T) =\frac{2^{m}-k-\sum\limits_{i=1}^{j} \left(2^{l_{i}}-l_{i}^{2}-1\right)}{n} - \frac{\left(2^{m}-k-\sum\limits_{i=1}^{j} \left(2^{l_{i}}-l_{i}-1\right)\right)^{2}}{n^{2}}. \end{aligned}}  $$
- If $\sum _{i=1}^{j} \left (2^{l_{i}-1}-1\right)> 2^{m}-k$
  11 $$ {\begin{aligned} V(T) =\frac{3\cdot 2^{m}-k-\sum\limits_{i=1}^{j} \left(2^{l_{i}}-l_{i}^{2}-1\right)}{n} - \frac{\left(3\cdot 2^{m}-k-\sum\limits_{i=1}^{j} \left(2^{l_{i}}-l_{i}-1\right)\right)^{2}}{n^{2}}. \end{aligned}}  $$

In particular, when *j*=0, the formula (10) applies and we obtain

$$V(F_{n})=V(T_{n;-})=\frac{2^{m}-k}{n}- \frac{(2^{m}-k)^{2}}{n^{2}}=\frac{2k(2^{m}-k)}{n^{2}} $$

in agreement with Remark 3.

### A general solution for a family of recurrences

For every *n*≥2 and for every 1≤*k*≤*n*−1, set

$$C_{k,n-k} \ {:=} \frac{1}{2}\binom{n}{k}\frac{(2k-3)!! (2(n-k)-3)!!}{(2n-3)!!}. $$

Notice that, since $\binom {n}{k}=\binom {n}{n-k}, C_{k,n-k}=C_{n-k,n}$.

It is straightforward to deduce from (2) that, for every $T\in \mathcal {BT}_{n}$ with *n*≥2, if $T_{k}\in \mathcal {BT}(X_{k})$ and $T^{\prime }_{n-k}\in \mathcal {BT}\left (X_{k}^{c}\right)$ (where $X_{k}\subsetneq [n]$, with |*X*_*k*_|=*k*, and $X_{k}^{c}=[n]\setminus X_{k}$) are its subtrees rooted at the children of its root, then

$$P_{U,n}(T)=\frac{2C_{k,n-k}}{\binom{n}{k}}P_{U,k}(T_{k})P_{U,n-k}\left(T'_{n-k}\right). $$

This will entail that the expected values under the uniform model appearing in this paper turn out to satisfy recurrences of the form

$$X_{n}=2\sum_{k=1}^{n-1} C_{k,n-k} X_{k}+p(n)+q(n)\cdot \frac{(2n-2)!!}{(2n-3)!!} $$

with $p(x),q(x)\in \mathbb {R}[x]$ polynomials without an independent term. The next proposition solves this kind of equation. We provide its proof in Section SN-4 in the Additional file 1.

#### **Proposition 6**

The solution *X*_*n*_ of the equation

$$X_{n}=2\sum_{k=1}^{n-1} C_{k,n-k} X_{k}+\sum_{l=1}^{r} a_{l}\binom{n}{l}+\frac{(2n-2)!!}{(2n-3)!!}\sum_{l=1}^{s} b_{l}\binom{n}{l} $$

(where *r*,*s*≥1 and $a_{1},\ldots,a_{r},b_{1},\ldots,b_{s}\in \mathbb {R}$) with given initial condition *X*_1_ is

$$X_{n}=\sum_{l=1}^{s+1} \widehat{a}_{l}\binom{n}{l}+\frac{(2n-2)!!}{(2n-3)!!}\sum_{l=1}^{r} \widehat{b}_{l}\binom{n}{l} $$

with

$$\begin{array}{*{20}l} & \widehat{a}_{1}= X_{1}-a_{1}\\ & \widehat{a}_{l} =\frac{l\cdot (2l-2)!!}{(2l-3)!!}\left(\frac{b_{l}}{l} +\frac{b_{l-1}}{l-1}\right),\quad l=2,\ldots,s\\ &\widehat{a}_{s+1} =\frac{(s+1)\cdot (2s)!!}{s\cdot (2s-1)!!}\cdot b_{s}\\ &\widehat{b}_{l}=\frac{(2l-3)!!}{(2l-2)!!}\cdot a_{l},\quad l=1,\ldots,r \end{array} $$

## Supplementary information

**Additional file 1** Proofs omitted in the main text.

## Data Availability

All R and Python scripts used in this paper, as well as all data used in it, are available at the GitHub repository page https://github.com/biocom-uib/var_depths.
