# Supplementary material for: On Sackin’s original proposal: the variance of the leaves’ depths as a phylogenetic balance index
Source: BMC Bioinformatics. 2020 Apr 23;21:154. doi: 10.1186/s12859-020-3405-1 (PMC7181513; doi:10.1186/s12859-020-3405-1)
Supplement: Supplementary file 1 — Additional file 1 Proofs omitted in the main text. [file 12859_2020_3405_MOESM1_ESM.pdf]

RESEARCH

On Sackin's original proposal: The variance of the leaves' depths as a phylogenetic balance index (Supplementary Material)

Tomás M. Coronado, Arnau Mir, Francesc Rosselló and Lucía Rotger

Abstract
This document contains the missing proofs in the main text of the paper "On Sackin's original proposal: The variance of the leaves' depths as a phylogenetic balance index."

SN-1 Further background

In this section we gather several definitions and preliminary results that were omitted in the main text's Background section because they are only used in this Supplementary Material.

Given $k \geqslant 2$ trees $T_1, \ldots, T_k$, with every $T_i \in \mathcal{T}^*_{n_i}$, their *root join* is the tree $T_1 \star T_2 \star \cdots \star T_k \in \mathcal{T}^*_{n_1+\cdots+n_k}$ obtained by connecting the roots of (disjoint copies of) $T_1, \ldots, T_k$ to a new common root r; see Fig. 8. In a similar way, given $k \geqslant 2$ phylogenetic trees $T_1 \in \mathcal{T}(X_1), \ldots, T_k \in \mathcal{T}(X_k)$, with $X_1, \ldots, X_k$ pairwise disjoint sets of labels, their *root join* is the phylogenetic tree $T_1 \star \cdots \star T_k \in \mathcal{T}\big(\bigcup_{j=1}^k X_j\big)$ obtained by connecting the roots of $T_1, \ldots, T_k$ to a new common root. Notice that the shape of the root join of a family of phylogenetic trees is the root join of their shapes, and that the root join of a pair of bifurcating trees is again bifurcating.

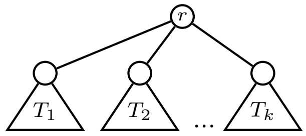

Figure 8 The tree $T_1 \star \cdots \star T_k$.

A probabilistic model of bifurcating phylogenetic trees P_n is *shape invariant* when, for every $n \geqslant 1$ and for every $T, T' \in \mathcal{BT}_n$, if T and T' have the same shape, then $P_n(T) = P_n(T')$. When P_n is shape invariant, it induces well-defined probability mappings $P_X : \mathcal{BT}(X) \to [0,1]$, for every set X of cardinality n, through any shape-preserving bijection $\mathcal{BT}(X) \leftrightarrow \mathcal{BT}_n$ induced by a bijection $X \leftrightarrow [n]$.

A probabilistic model of bifurcating phylogenetic trees P_n is *Markovian* when there exists a symmetric mapping $q : \mathbb{N} \times \mathbb{N} \to \mathbb{R}$, called the *conditional split distribution* of P_n, such that, for every $T_k \in \mathcal{BT}(X_k)$ and $T'_l \in \mathcal{BT}(X_l)$, with X_k and X_l disjoint sets of cardinalities $|X_k| = k$ and $|X_l| = l$, $P_{X_k \cup X_l}(T_k \star T'_l) = q(k,l) P_{X_k}(T_k) P_{X_l}(T'_l)$.

The formulas (1) and (2) in the main text clearly imply that both the Yule and the uniform models are shape invariant. As to their Markovianity, it is easy to deduce from those formulas that

$$P_{Y,n}(T_k \star T'_{n-k}) = \frac{2}{(n-1)\binom{n}{k}} P_{Y,k}(T_k) P_{Y,n-k}(T'_{n-k}) \tag{11}$$

$$\begin{aligned} P_{U,n}(T_k \star T'_{n-k}) &= \frac{(2k-3)!! \cdot (2(n-k)-3)!!}{(2n-3)!!} P_{U,k}(T_k) P_{U,n-k}(T'_{n-k}) \\ &= \frac{2C_{k,n-k}}{\binom{n}{k}} P_{U,k}(T_k) P_{U,n-k}(T'_{n-k}) \end{aligned} \tag{12}$$

where

$$C_{k,n-k} := \frac{1}{2}\binom{n}{k} \frac{(2k-3)!!(2(n-k)-3)!!}{(2n-3)!!}.$$

Consider the random variables S_n and Φ_n that take a phylogenetic tree $T \in \mathcal{BT}_n$ and compute $S(T)$ and $\Phi(T)$, respectively. Their expected values under the Yule model (denoted by E_Y) and the uniform model (denoted by E_U), as well as their variance under the Yule model (denoted by σ_Y^2), are known:

$$E_Y(S_n) = 2n(H_n - 1) \qquad [3, \text{Appendix}] \tag{13}$$

$$E_U(S_n) = n\Big(\frac{(2n-2)!!}{(2n-3)!!} - 1\Big) \qquad [5, \text{Thm. } 22] \tag{14}$$

$$\sigma_Y^2(S_n) = 7n^2 - n - 2nH_n - 4n^2 H_n^{(2)} \qquad [1, \text{Cor. } 1] \tag{15}$$

$$E_Y(\Phi_n) = n(n+1) - 2nH_n \qquad [5, \text{Thm. } 17] \tag{16}$$

$$E_U(\Phi_n) = \frac{1}{2}\binom{n}{2}\left(\frac{(2n-2)!!}{(2n-3)!!} - 2\right) \qquad [5, \text{Thm. } 23] \tag{17}$$

$$\sigma_Y^2(\Phi_n) = \frac{n^4 - 10n^3 + 131n^2 - 2n}{12} - 6nH_n - 4n^2 H_n^{(2)} \qquad [1, \text{Cor. } 3] \tag{18}$$

where $H_n = \sum_{i=1}^{n} 1/i$, the n-th *harmonic number*, and $H_n^{(2)} = \sum_{i=1}^{n} 1/i^2$.

SN-2 Proof of Proposition 5

Proposition 5 *For every $T \in \mathcal{BT}^*_n$, the following conditions are equivalent:*

(a) T is of type F_n.

(b) There exists a $d_0 \in \mathbb{N}$ such that $\delta_T(x) \in \{d_0, d_0+1\}$ for every $x \in L(T)$.

(c) $|\delta_T(x) - \widehat{S}(T)| < 1$ for every $x \in L(T)$.

(d) T is depth-equivalent to B_n.

Proof The implications (a)⇒(b)⇔(c) are straightforward:

- By construction, if T is of type F_n with $n = 2^m + k$, then $\Delta(T)$ consists of $2k$ leaves of depth $m+1$ and $2^m - k$ leaves of depth m.
- If $\Delta(T)$ consists of l leaves of depth d_0 and $n-l$ leaves of depth d_0+1, then

$$\widehat{S}(T) = d_0 + \frac{n-l}{n}$$

and therefore each $|\delta_T(x) - \widehat{S}(T)|$ is either $(n-l)/n$ or l/n. If $0 < l < n$, these two values are smaller than 1, and if $l = 0$ or $l = n$, then all leaves in T have the same depth and therefore $|\delta_T(x) - \widehat{S}(T)| = 0$ for every $x \in L(T)$.

- If $|\delta_T(x) - \widehat{S}(T)| < 1$, then, since $\delta_T(x) \in \mathbb{N}$, $\delta_T(x) \in \{\lfloor \widehat{S}(T) \rfloor, \lceil \widehat{S}(T) \rceil\}$.

As far as (b)⇒(a) goes, we prove it by induction on n. The base case when $n = 1$ is obvious, because the only tree with one leaf is the fully symmetric tree B_1. Assume now that (b)⇒(a) is true for every tree in $\mathcal{BT}^*_n$ and let T be a bifurcating tree with $n+1$ leaves for which there exists a $d_0 \in \mathbb{N}$ such that $\delta_T(x) \in \{d_0, d_0+1\}$ for every $x \in L(T)$; to simplify the discussion, we shall assume that $\delta(T) = d_0 + 1$. Let x_0 be a leaf in T of depth $d_0 + 1$ and let $T' \in \mathcal{BT}^*_n$ be obtained by replacing the cherry that contained x_0 by a leaf of depth d_0. Then, $\delta_{T'}(x) \in \{d_0, d_0+1\}$ for every $x \in L(T')$, too. Thus, by the induction hypothesis, T' is of type F_n. Finally, since T is obtained from a tree of type F_n by replacing a leaf of minimum depth, d_0, by a cherry with leaves of depth one unit larger, T is of type F_{n+1}.

This completes the proof that conditions (a), (b) and (c) are equivalent.

Let us prove now that if a bifurcating tree T satisfies (d) then it satisfies (b) with $d_0 = \lfloor \log_2(n) \rfloor$, by induction on the depth of the tree. This implication is trivially true when $\delta(T) = 0$, because the only tree of depth 0 is B_1. Assume now that the implication is true for every tree of depth at most δ and let $T \in \mathcal{BT}^*_n$ be a bifurcating tree of depth $\delta + 1$ that is depth-equivalent to B_n. Since (b) is an assertion on the depths of the leaves of T, and $\Delta(T) = \Delta(B_n)$, in order to prove that T satisfies (b) we can assume without any loss of generality that $T = B_n$. Let $m = \lfloor \log_2(n) \rfloor$ and $k = n - 2^m$.

Let T_1 and T_2 be the subtrees rooted at the children of the root of T and n_1 and n_2 their respective numbers of leaves, with $n_1 \leqslant n_2$ and $n = n_1 + n_2$. Since T is maximally balanced, T_1 and T_2 are also maximally balanced and $n_1 = \lfloor n/2 \rfloor$ and $n_2 = \lceil n/2 \rceil$. Then, since $\delta(T_1), \delta(T_2) \leqslant \delta = \delta(T) - 1$, by the induction hypothesis we deduce that if, for every $i = 1, 2$, we set $d_i = \lfloor \log_2(n_i) \rfloor$, then $\delta_{T_i}(x) \in \{d_i, d_i + 1\}$ for every $x \in L(T_i)$. Now:

- If $k < 2^{m+1} - 1$, then $d_1 = d_2 = m - 1$.
- If $k = 2^{m+1} - 1$, then $n_1 = 2^m - 1$, and thus $d_1 = m - 1$, and $n_2 = 2^m$, and thus $d_2 = m$; but then, T_2 is fully symmetric, because it is maximally balanced with 2^m leaves, which implies in particular that all its leaves have depth m.

Then, in both cases, $\delta_{T_i}(x) \in \{m-1, m\}$ for every $x \in L(T_i)$ and $i = 1, 2$. Since $\delta_T(x) = \delta_{T_i}(x) + 1$ if $x \in L(T_i)$, we conclude that $\delta_T(x) \in \{m, m+1\}$ for every $x \in L(T)$, where $m = \lfloor \log_2(n) \rfloor$. This is what we wanted to prove, and hence the implication (d)⇒(b) is established.

Finally, we prove the implication (a)⇒(d). Let $T \in \mathcal{BT}^*_n$ be of type F_n. Since we have already proved that (d)⇒(b)⇒(a), we know that B_n is also of type F_n. But then, by Remark 3 in the main text, T and B_n are depth-equivalent. □

SN-3 Proofs of Lemmas 1 and 2

Recall that we denote the numbers of leaves of depths $\delta(T)$ and $\delta(T) - 1$ of a tree $T \in \mathcal{BT}^*_n$ by $p_0(T)$ and $p_1(T)$, respectively.

Lemma 1 *Let $n = 2^m + k$ with $m = \lfloor \log_2(n) \rfloor$ and $k = n - 2^m$. For every tree T of type $T_{n;l_1,\ldots,l_j}$, with $j \geqslant 0$ and $2 \leqslant l_1 < \cdots < l_j \leqslant \delta(T) - 2$:*
(a) If $k + \sum_{i=1}^{j}(2^{l_i} - 1) = 0$, then $p_1(T) = 0$ and the tree is fully symmetric.
(b) If $0 < k + \sum_{i=1}^{j}(2^{l_i} - 1) \leqslant 2^m$, then $p_1(T) = 2^m - k - \sum_{i=1}^{j}(2^{l_i} - 1)$ and $\delta(T) = m + 1$.
(c) If $k + \frac{1}{2}\sum_{i=1}^{j}(2^{l_i} - 2) > 2^m$, then $p_1(T) = 3 \cdot 2^m - k - \sum_{i=1}^{j}(2^{l_i} - 1)$ and $\delta(T) = m + 2$.
(d) If $k + \frac{1}{2}\sum_{i=1}^{j}(2^{l_i} - 2) \leqslant 2^m < k + \sum_{i=1}^{j}(2^{l_i} - 1)$, then there does not exist any tree T of type $T_{n;l_1,\ldots,l_j}$.

Proof Let $n = 2^m + k$ with $0 \leqslant k < 2^m$, let $T \in \mathcal{BT}_n^*$ be a tree of type $T_{n;l_1,\ldots,l_j}$, with $2 \leqslant l_1 < \cdots < l_j \leqslant \delta(T) - 2$, and set $\delta = \delta(T)$ and $p_1 = p_1(T)$.

If $j = 0$, T has only leaves of depths δ and $\delta - 1$ and then it is of type F_n. In particular, in this case, if $k = 0$, T is fully symmetric of depth m, and hence $p_1 = 0$, while if $k > 0$, then $p_1 = 2^m - k$ and $\delta = m+1$. This proves (a) as well as (b) when $j = 0$. So, we shall assume henceforth that $j \geqslant 1$.

To begin with, notice that, in order to complete T to a fully symmetric tree with 2^δ leaves, we must append a cherry to each leaf of depth $\delta - 1$, which adds p_1 new leaves, and we must append a fully symmetric tree of depth l_i to each leaf of depth $\delta - l_i$, thus adding for each such leaf $2^{l_i} - 1$ new leaves. This implies that

$$2^\delta = n + p_1 + \sum_{i=1}^{j}(2^{l_i} - 1). \tag{19}$$

Since we are assuming that $j > 0$, this implies that $n < 2^\delta$ and hence $m \leqslant \delta - 1$. On the other hand, since $p_1 < n < 2^{m+1}$, we have that

$$2^\delta = n + p_1 + \sum_{i=1}^{j}(2^{l_i} - 1) < 2n + \sum_{i=2}^{\delta-2}(2^i - 1) < 2^{m+2} + 2^{\delta-1},$$

which implies that $2^{\delta-1} < 2^{m+2}$ and therefore $\delta - 1 \leqslant m + 1$. So, in summary, $m + 1 \leqslant \delta \leqslant m + 2$.

Now, on the one hand, if $\delta = m + 1$, (19) implies that

$$p_1 = 2^m - k - \sum_{i=1}^{j}(2^{l_i} - 1).$$

In particular, in this case, $k + \sum_{i=1}^{j}(2^{l_i} - 1) \leqslant 2^m$ because $p_1 \geqslant 0$.

On the other hand, if $\delta = m + 2$, (19) implies that

$$p_1 = 3 \cdot 2^m - k - \sum_{i=1}^{j}(2^{l_i} - 1).$$

And in this case, since T contains at least 2 leaves of depth δ and j leaves of depths different from δ or $\delta - 1$, it must happen that $p_1 \leqslant n - j - 2$, that is

$$3 \cdot 2^m - k - \sum_{i=1}^{j}(2^{l_i} - 1) \leqslant 2^m + k - j - 2,$$

which is equivalent to $k + \frac{1}{2}\sum_{i=1}^{j}(2^{l_i} - 2) > 2^m$.

Since $\delta = m + 1$ or $\delta = m + 2$, this completes the proof of the statement. □

Lemma 2 *If T is a tree of type $T_{n;l_1,\dots,l_j}$, then*

$$V(T) = \frac{1}{n^2}\left(n\Big(p_1(T) + \sum_{i=1}^{j} l_i^2\Big) - \Big(p_1(T) + \sum_{i=1}^{j} l_i\Big)^2\right).$$

Proof Set $\delta = \delta(T)$, $p_0 = p_0(T)$, and $p_1 = p_1(T)$. Since $n = p_0 + p_1 + j$,

$$\widehat{S}(T) = \frac{p_0\delta + p_1(\delta - 1) + \sum_{i=1}^{j}(\delta - l_i)}{n} = \delta - \frac{p_1 + \sum_{i=1}^{j} l_i}{n}$$

and hence

$$\begin{aligned}
n \cdot V(T) &= p_0(\delta - \widehat{S}(T))^2 + p_1(\delta - 1 - \widehat{S}(T))^2 + \sum_{i=1}^{j}(\delta - l_i - \widehat{S}(T))^2 \\
&= p_0\Big(\frac{p_1 + \sum_{i=1}^{j} l_i}{n}\Big)^2 + p_1\Big(\frac{p_1 + \sum_{i=1}^{j} l_i}{n} - 1\Big)^2 + \sum_{i=1}^{j}\Big(\frac{p_1 + \sum_{i=1}^{j} l_i}{n} - l_i\Big)^2 \\
&= p_0\Big(\frac{p_1 + \sum_{i=1}^{j} l_i}{n}\Big)^2 + p_1\Big(\frac{p_1 + \sum_{i=1}^{j} l_i}{n}\Big)^2 - 2p_1\Big(\frac{p_1 + \sum_{i=1}^{j} l_i}{n}\Big) + p_1 \\
&\qquad + j\Big(\frac{p_1 + \sum_{i=1}^{j} l_i}{n}\Big)^2 - 2\Big(\sum_{i=1}^{j} l_i\Big)\Big(\frac{p_1 + \sum_{i=1}^{j} l_i}{n}\Big) + \sum_{i=1}^{j} l_i^2 \\
&= n\Big(\frac{p_1 + \sum_{i=1}^{j} l_i}{n}\Big)^2 - 2\Big(p_1 + \sum_{i=1}^{j} l_i\Big)\Big(\frac{p_1 + \sum_{i=1}^{j} l_i}{n}\Big) + p_1 + \sum_{i=1}^{j} l_i^2 \\
&= p_1 + \sum_{i=1}^{j} l_i^2 - \frac{(p_1 + \sum_{i=1}^{j} l_i)^2}{n}.
\end{aligned}$$

□

SN-4 Proof of Proposition 6

In this section, and henceforth, ${}_pF_q$ denotes the (*generalized*) *hypergeometric function* defined by

$${}_pF_q\left[\begin{matrix} a_1, & \dots, & a_p \\ b_1, & \dots, & b_q \end{matrix}; z\right] = \sum_{k\geqslant 0} \frac{(a_1)_k \cdots (a_p)_k}{(b_1)_k \cdots (b_q)_k} \cdot \frac{z^k}{k!},$$

where $(a)_0 = 1$ and $(a)_k := a \cdot (a+1) \cdots (a+k-1)$ for $k \geqslant 1$. Recall, moreover, that for every $n \geqslant 2$ and for every $1 \leqslant k \leqslant n-1$,

$$C_{k,n-k} := \frac{1}{2}\binom{n}{k}\frac{(2k-3)!!(2(n-k)-3)!!}{(2n-3)!!}.$$

We start by proving two auxiliary lemmas. These lemmas will be used not only in the proof of Proposition 6, but also later in the proofs of the main results in Sections SN-10 to SN-12.

Lemma 3 *For every $n \geqslant 2$:*

(a) $\sum_{k=1}^{n-1} C_{k,n-k} = 1$

(b) For every $m \geqslant 1$,

$$\sum_{k=1}^{n-1} C_{k,n-k}\binom{k}{m} = \frac{1}{2}\binom{n}{m}\Big(1 - \frac{m-1}{n-1}\cdot\frac{(2m-3)!!}{(2m-2)!!}\cdot\frac{(2n-2)!!}{(2n-3)!!}\Big).$$

Proof We consider first the case when $1 \leqslant m \leqslant n-1$. In this case

$$\begin{aligned}\sum_{k=1}^{n-1} C_{k,n-k}\binom{k}{m} &= \sum_{k=m}^{n-1} C_{k,n-k}\binom{k}{m} = \sum_{k=m}^{n-1}\frac{n!(2k-3)!!(2n-2k-3)!!k!}{2\cdot k!(n-k)!(2n-3)!!m!(k-m)!}\\ &= \frac{n!}{2\cdot m!(2n-3)!!}\sum_{k=m}^{n-1}\frac{(2k-2)!(2n-2k-2)!}{2^{k-1}(k-1)!2^{n-k-1}(n-k-1)!(n-k)!(k-m)!}\\ &= \frac{n!}{2^{n-1}\cdot m!(2n-3)!!}\sum_{k=m}^{n-1}\frac{(2k-2)!(2n-2k-2)!}{(k-1)!(n-k-1)!(n-k)!(k-m)!}\\ &= \frac{n!}{2^{n-1}\cdot m!(2n-3)!!}\sum_{k=0}^{n-m-1}\frac{(2k+2m-2)!(2n-2k-2m-2)!}{(k+m-1)!(n-k-m-1)!(n-k-m)!k!}\end{aligned} \tag{20}$$

We shall compute now this last sum using the *lookup algorithm* given in [6, p. 36]. Take

$$t_j = \frac{(2j+2m-2)!(2n-2j-2m-2)!}{(j+m-1)!(n-j-m-1)!(n-j-m)!j!}$$

Then

$$t_0 = \frac{(2m-2)!(2n-2m-2)!}{(m-1)!(n-m-1)!(n-m)!}, \qquad \frac{t_{j+1}}{t_j} = \frac{(j+m-1/2)(j+m-n)}{(j+m+3/2-n)(j+1)}.$$

But now, it is wrong to deduce from the lookup algorithm that

$$\begin{aligned}&\sum_{k=0}^{n-m-1}\frac{(2k+2m-2)!(2n-2k-2m-2)!}{(k+m-1)!(n-k-m-1)!(n-k-m)!k!}\\ &\quad= \frac{(2m-2)!(2n-2m-2)!}{(m-1)!(n-m-1)!(n-m)!}\cdot {}_2F_1\left[\begin{matrix} m-\frac{1}{2} & m-n \\ m+\frac{3}{2}-n & \end{matrix};1\right]\end{aligned}$$

because $(m-n)_k = 0$ for every $k > n-m$, but

$$(m-n)_{n-m} = (m-n)(m-n+1)\cdots(-1) = (-1)^{n-m}(n-m)! \neq 0,$$

and therefore

$${}_2F_1\left[\begin{matrix} m-\frac{1}{2} & m-n \\ m+\frac{3}{2}-n & \end{matrix};1\right] = \sum_{k=0}^{n-m}\frac{\left(m-\frac{1}{2}\right)_k(m-n)_k}{\left(m+\frac{3}{2}-n\right)_k k!}$$

while the index k in our original sum stops a $n-m-1$. Therefore, what the lookup algorithm actually implies is that

$$\sum_{k=0}^{n-m-1} \frac{(2k+2m-2)!(2n-2k-2m-2)!}{(k+m-1)!(n-k-m-1)!(n-k-m)!k!}$$
$$= \frac{(2m-2)!(2n-2m-2)!}{(m-1)!(n-m-1)!(n-m)!}\left({}_2F_1\left[\begin{matrix} m-\frac{1}{2} & m-n \\ m+\frac{3}{2}-n & \end{matrix};1\right] - \frac{\left(m-\frac{1}{2}\right)_{n-m}(m-n)_{n-m}}{\left(m+\frac{3}{2}-n\right)_{n-m}(n-m)!}\right). \tag{21}$$

The subtrahend in this expression can be computed using that $(m-n)_{n-m} = (-1)^{n-m}(n-m)!$ and

$$\left(m-\frac{1}{2}\right)_{n-m} = \left(m-\frac{1}{2}\right)\left(m+\frac{1}{2}\right)\cdots\left(n-\frac{3}{2}\right) = \frac{(2n-3)!!}{2^{n-m}\cdot(2m-3)!!}$$
$$\left(m+\frac{3}{2}-n\right)_{n-m} = \left(m+\frac{3}{2}-n\right)\left(m+\frac{5}{2}-n\right)\cdots\left(-\frac{1}{2}\right)\cdot\frac{1}{2}$$
$$= \frac{(-1)^{n-m-1}(2n-2m-3)!!}{2^{n-m}}$$

and its value is then

$$\frac{\left(m-\frac{1}{2}\right)_{n-m}(m-n)_{n-m}}{\left(m+\frac{3}{2}-n\right)_{n-m}(n-m)!}$$
$$= \frac{(2n-3)!!(-1)^{n-m}(n-m)!2^{n-m}}{2^{n-m}(2m-3)!!(-1)^{n-m-1}(2n-2m-3)!!(n-m)!}$$
$$= -\frac{(2n-3)!!}{(2m-3)!!(2n-2m-3)!!} \tag{22}$$

As to the ${}_2F_1$ hypergeometric function in (21), since $m \leqslant n$, we can apply the identity http://functions.wolfram.com/07.23.03.0003.01 and we obtain

$${}_2F_1\left[\begin{matrix} m-\frac{1}{2} & m-n \\ m+\frac{3}{2}-n & \end{matrix};1\right] = \frac{(2-n)_{n-m}}{\left(m+\frac{3}{2}-n\right)_{n-m}}$$

and since

$$(2-n)_{n-m} = (2-n)(3-n)\cdots(1-m) = \begin{cases} 0 & \text{if } m=1 \\ (-1)^{n-m}\frac{(n-2)!}{(m-2)!} & \text{if } m>1 \end{cases}$$
$$= (-1)^{n-m}(m-1)\frac{(n-2)!}{(m-1)!}$$

and, as we have seen $\left(m+\frac{3}{2}-n\right)_{n-m} = (-1)^{n-m-1}2^{m-n}(2n-2m-3)!!$, we have

$${}_2F_1\left[\begin{matrix} m-\frac{1}{2} & m-n \\ m+\frac{3}{2}-n & \end{matrix};1\right] = -\frac{2^{n-m}(m-1)(n-2)!}{(m-1)!(2n-2m-3)!!} \tag{23}$$

Then, combining (20), (21), (22), and (23), we obtain

$$\begin{aligned}
&\sum_{k=1}^{n-1} C_{k,n-k}\binom{k}{m}\\
&= \frac{n!(2m-2)!(2n-2m-2)!}{2^{n-1}\cdot m!(2n-3)!!(m-1)!(n-m-1)!(n-m)!}\\
&\qquad\cdot\Big(\frac{(2n-3)!!}{(2m-3)!!(2n-2m-3)!!} - \frac{2^{n-m}(m-1)(n-2)!}{(m-1)!(2n-2m-3)!!}\Big)\\
&= \frac{1}{2}\binom{n}{m}\Big(1-\frac{m-1}{n-1}\cdot\frac{(2m-3)!!}{(2m-2)!!}\cdot\frac{(2n-2)!!}{(2n-3)!!}\Big).
\end{aligned}$$

This proves (b) when $1 \leqslant m \leqslant n-1$. Now notice that when $m = n \geqslant 2$,

$$\sum_{k=1}^{n-1} C_{k,n-k}\binom{k}{n} = 0 = \frac{1}{2}\binom{n}{n}\Big(1-\frac{n-1}{n-1}\cdot\frac{(2n-3)!!}{(2n-2)!!}\cdot\frac{(2n-2)!!}{(2n-3)!!}\Big),$$

and when $m > n$,

$$\sum_{k=1}^{n-1} C_{k,n-k}\binom{k}{m} = 0 = \binom{n}{m}.$$

Therefore the identity stated in (b) also holds when $m \geqslant n$.

Finally, as far as (a) goes, by the symmetry of $C_{k,n-k}$, we have that

$$\sum_{k=1}^{n-1} C_{k,n-k}k = \sum_{k=1}^{n-1} C_{k,n-k}(n-k)$$

from which we deduce that

$$\sum_{k=1}^{n-1} C_{k,n-k} = \frac{2}{n}\sum_{k=1}^{n-1} C_{k,n-k}k = \frac{2}{n}\cdot\frac{n}{2} = 1,$$

where the second equality is simply (b) for $m = 1$. □

Lemma 4 *For every $n \geqslant 2$,*

(a) $\displaystyle\sum_{k=1}^{n-1} C_{k,n-k}\cdot\frac{(2k-2)!!}{(2k-3)!!} = \frac{1}{2}\cdot\frac{(2n-2)!!}{(2n-3)!!} + \frac{1}{4}\big(2H_{2n-2} - H_{n-1} - 2\big).$

(b) For every $m \geqslant 1$,

$$\sum_{k=1}^{n-1} C_{k,n-k}\binom{k}{m}\frac{(2k-2)!!}{(2k-3)!!} = \frac{1}{2}\binom{n}{m}\Big(\frac{(2n-2)!!}{(2n-3)!!} - \frac{(2m-2)!!}{(2m-3)!!}\Big).$$

Proof We consider again first the case when $1 \leqslant m \leqslant n-1$. Let us develop our sum of interest:

$$\begin{aligned}
\sum_{k=1}^{n-1} C_{k,n-k}\binom{k}{m}\frac{(2k-2)!!}{(2k-3)!!} &= \sum_{k=m}^{n-1} C_{k,n-k}\binom{k}{m}\frac{(2k-2)!!}{(2k-3)!!} \\
&= \sum_{k=m}^{n-1} \frac{n!(2k-3)!!(2n-2k-3)!!k!(2k-2)!!}{2\cdot k!(n-k)!(2n-3)!!(k-m)!m!(2k-3)!!} \\
&= \frac{n!}{2\cdot m!(2n-3)!!}\sum_{k=m}^{n-1}\frac{(2n-2k-3)!!(2k-2)!!}{(n-k)!(k-m)!} \\
&= \frac{n!}{2\cdot m!(2n-3)!!}\sum_{k=m}^{n-1}\frac{(2n-2k-2)!2^{k-1}(k-1)!}{2^{n-k-1}(n-k-1)!(n-k)!(k-m)!} \\
&= \frac{n!}{2^{n+1}\cdot m!(2n-3)!!}\sum_{k=m}^{n-1}\frac{(2n-2k-2)!(k-1)!2^{2k}}{(n-k-1)!(n-k)!(k-m)!} \\
&= \frac{n!}{2^{n-2m+1}\cdot m!(2n-3)!!}\sum_{k=0}^{n-m-1}\frac{(2n-2k-2m-2)!(k+m-1)!2^{2k}}{(n-k-m-1)!(n-k-m)!k!}
\end{aligned} \tag{24}$$

We shall apply again the lookup algorithm to compute this sum. Taking

$$t_j = \frac{(2n-2j-2m-2)!(j+m-1)!2^{2j}}{(n-j-m-1)!(n-j-m)!j!}$$

we have

$$t_0 = \frac{(2n-2m-2)!(m-1)!}{(n-m-1)!(n-m)!}, \qquad \frac{t_{j+1}}{t_j} = \frac{(j+m)(j+m-n)}{(j+m-n+3/2)(j+1)}$$

Then, arguing as in the corresponding step in the proof of Lemma 3 we obtain that, by the lookup algorithm and taking into account that $(m-n)_k = 0$ for every $k > n-m$,

$$\begin{aligned}
&\sum_{k=0}^{n-m-1}\frac{(2n-2k-2m-2)!(k+m-1)!2^{2k}}{(n-k-m-1)!(n-k-m)!k!} \\
&\quad= \frac{(2n-2m-2)!(m-1)!}{(n-m-1)!(n-m)!}\left({}_2F_1\left[\begin{matrix} m & m-n \\ m+\frac{3}{2}-n & \end{matrix};1\right]\right. \\
&\qquad\left. - \frac{(m)_{n-m}(m-n)_{n-m}}{\left(m+\frac{3}{2}-n\right)_{n-m}(n-m)!}\right)
\end{aligned} \tag{25}$$

Since $m \leqslant n$, the first summand inside the parenthesis is equal, by identity http://functions.wolfram.com/07.23.03.0003.01, to

$${}_2F_1\left[\begin{matrix} m & m-n \\ m+\frac{3}{2}-n & \end{matrix};1\right] = \frac{\left(\frac{3}{2}-n\right)_{n-m}}{\left(\frac{3}{2}+m-n\right)_{n-m}}$$

and since

$$\left(\frac{3}{2}-n\right)_{n-m}=\left(\frac{3}{2}-n\right)\left(\frac{5}{2}-n\right)\cdots\left(\frac{3}{2}-m-1\right)=\frac{(-1)^{n-m}(2n-3)!!}{2^{n-m}\cdot(2m-3)!!}$$

and, as we established in the proof of Lemma 3 (page 7), $\left(m+\frac{3}{2}-n\right)_{n-m}=(-1)^{n-m-1}2^{m-n}(2n-2m-3)!!$, we have

$${}_2F_1\left[\begin{matrix}m & m-n\\ m+\frac{3}{2}-n & \end{matrix};1\right]=-\frac{(2n-3)!!}{(2m-3)!!(2n-2m-3)!!}$$

As for the subtrahend, we have

$$\begin{aligned}\frac{(m)_{n-m}(m-n)_{n-m}}{\left(m+\frac{3}{2}-n\right)_{n-m}(n-m)!}&=\frac{(n-1)!(-1)^{n-m}(n-m)!2^{n-m}}{(m-1)!(-1)^{n-m-1}(2n-2m-3)!!(n-m)!}\\&=-\frac{(n-1)!2^{n-m}}{(m-1)!(2n-2m-3)!!}\end{aligned}$$

So, by (24) and (25),

$$\begin{aligned}&\sum_{k=1}^{n-1}C_{k,n-k}\binom{k}{m}\cdot\frac{(2k-2)!!}{(2k-3)!!}\\&\quad=\frac{n!(2n-2m-2)!(m-1)!}{2^{n-2m+1}\cdot m!(2n-3)!!(n-m-1)!(n-m)!}\\&\qquad\qquad\cdot\Big(\frac{(n-1)!2^{n-m}}{(m-1)!(2n-2m-3)!!}-\frac{(2n-3)!!}{(2m-3)!!(2n-2m-3)!!}\Big)\\&\quad=\frac{1}{2}\binom{n}{m}\Big(\frac{(2n-2)!!}{(2n-3)!!}-\frac{(2m-2)!!}{(2m-3)!!}\Big)\end{aligned}$$

This finishes the proof of (b) in the case when $1\leqslant m\leqslant n-1$. Now, it is clear that when $m\geqslant n$

$$\sum_{k=1}^{n-1}C_{k,n-k}\binom{k}{m}\cdot\frac{(2k-2)!!}{(2k-3)!!}=0=\binom{n}{m}\Big(\frac{(2n-2)!!}{(2n-3)!!}-\frac{(2m-2)!!}{(2m-3)!!}\Big)$$

and therefore the equality in (b) holds actually for every $m\geqslant 1$.

It remains to cover the case when $m = 0$, i.e., (a). We start again by developing the sum of interest.

$$\begin{aligned}
\sum_{k=1}^{n-1} C_{k,n-k} \frac{(2k-2)!!}{(2k-3)!!} &= \sum_{k=1}^{n-1} \frac{n!(2k-3)!!(2n-2k-3)!!(2k-2)!!}{2\cdot k!(n-k)!(2n-3)!!(2k-3)!!} \\
&= \frac{n!}{2\cdot(2n-3)!!} \sum_{k=1}^{n-1} \frac{(2n-2k-3)!!(2k-2)!!}{k!(n-k)!} \\
&= \frac{n!}{2\cdot(2n-3)!!} \sum_{k=1}^{n-1} \frac{(2n-2k-2)!2^{k-1}(k-1)!}{2^{n-k-1}(n-k-1)!(n-k)!k!} \\
&= \frac{n!}{2^{n+1}\cdot(2n-3)!!} \sum_{k=1}^{n-1} \frac{(2n-2k-2)!2^{2k}}{(n-k-1)!(n-k)!k} \\
&= \frac{n!}{2^{n-1}\cdot(2n-3)!!} \sum_{k=0}^{n-2} \frac{(2n-2k-4)!2^{2k}}{(n-k-2)!(n-k-1)!(k+1)} \qquad (26)
\end{aligned}$$

We apply again the lookup algorithm. Taking

$$t_j = \frac{(2n-2j-4)!2^{2j}}{(n-j-2)!(n-j-1)!(j+1)}$$

we have

$$t_0 = \frac{(2n-4)!}{(n-2)!(n-1)!}, \qquad \frac{t_{j+1}}{t_j} = \frac{(j+1)^2(j+1-n)}{(j+2)(j-n+5/2)(j+1)}$$

and therefore, by the lookup algorithm and taking into account that $(1-n)_k = 0$ for every $k \geqslant n$ but $(1-n)_{n-1} = (-1)^{n-1}(n-1)!$, we have that

$$\begin{aligned}
&\sum_{k=0}^{n-2} \frac{(2n-2k-4)!2^{2k}}{(n-k-2)!(n-k-1)!(k+1)} \\
&\quad = \frac{(2n-4)!}{(n-2)!(n-1)!} \left({}_3F_2 \left[\begin{matrix} 1 & 1 & 1-n \\ 2 & \frac{5}{2}-n & \end{matrix} ; 1 \right] \right. \\
&\qquad \left. - \frac{(1)_{n-1}^2 (1-n)_{n-1}}{(2)_{n-1}\left(\frac{5}{2}-n\right)_{n-1}(n-1)!} \right) \qquad (27)
\end{aligned}$$

Now, since $(1)_{n-1} = (n-1)!$, $(2)_{n-1} = n!$, $(1-n)_{n-1} = (-1)^{n-1}(n-1)!$, and $(5/2-n)_{n-1} = (-1)^{n-2}(2n-5)!!/2^{n-1}$, the subtrahend in this last expression is

$$\frac{(1)_{n-1}^2(1-n)_{n-1}}{(2)_{n-1}\left(\frac{5}{2}-n\right)_{n-1}(n-1)!} = \frac{(-1)^{n-1}(n-1)!^3 2^{n-1}}{n!(-1)^{n-2}(2n-5)!!(n-1)!} = -\frac{(2n-2)!!}{n\cdot(2n-5)!!}. \qquad (28)$$

As to the ${}_3F_2$ hypergeometric series in (27), applying transformation (3.1.2) in [2] we obtain

$$ {}_3F_2\left[\begin{matrix} 1 & 1 & 1-n \\ 2 & \frac{5}{2}-n & \end{matrix}; 1\right] = \frac{\Gamma(2)\Gamma\left(\frac{5}{2}-n\right)\Gamma\left(\frac{3}{2}\right)}{\Gamma(1)\Gamma\left(\frac{5}{2}\right)\Gamma\left(\frac{5}{2}-n\right)} {}_3F_2\left[\begin{matrix} 1 & \frac{3}{2}-n & \frac{3}{2} \\ \frac{5}{2} & \frac{5}{2}-n & \end{matrix}; 1\right] = \frac{2}{3}\cdot {}_3F_2\left[\begin{matrix} \frac{3}{2} & 1 & \frac{3}{2}-n \\ \frac{5}{2} & \frac{5}{2}-n & \end{matrix}; 1\right] $$

and, by identity http://functions.wolfram.com/07.27.03.0017.01,

$$ \begin{aligned} &{}_3F_2\left[\begin{matrix} \frac{3}{2} & 1 & \frac{3}{2}-n \\ \frac{5}{2} & \frac{5}{2}-n & \end{matrix}; 1\right] \\ &= \frac{\left(\frac{3}{2}-n\right)\left(-\frac{1}{2}\right)_n (n-1)!\Gamma\left(\frac{5}{2}\right)\Gamma(1)}{\frac{1}{2}\left(-\frac{1}{2}\right)_n (1)_n \Gamma(1)\Gamma\left(\frac{3}{2}\right)} \sum_{k=0}^{n-1} \frac{\left(-\frac{1}{2}\right)_k (1)_k}{\left(\frac{1}{2}\right)_k k!} \\ &= -\frac{9-6n}{2n}\sum_{k=0}^{n-1}\frac{1}{2k-1} = -\frac{9-6n}{2n}\left(-1+\sum_{j=1}^{2n-2}\frac{1}{j}-\frac{1}{2}\sum_{j=1}^{n-1}\frac{1}{j}\right) \\ &= -\frac{9-6n}{2n}\left(H_{2n-2}-\frac{1}{2}H_{n-1}-1\right) \end{aligned} $$

So,

$$ \begin{aligned} {}_3F_2\left[\begin{matrix} 1 & 1 & 1-n \\ 2 & \frac{5}{2}-n & \end{matrix}; 1\right] &= -\frac{2}{3}\cdot\frac{9-6n}{2n}\left(H_{2n-2}-\frac{1}{2}H_{n-1}-1\right) \\ &= -\frac{3-2n}{2n}\left(2H_{2n-2}-H_{n-1}-2\right) \end{aligned} $$

and finally, combining this identity with (26), (27) and (28), we obtain

$$ \begin{aligned} &\sum_{k=1}^{n-1} C_{k,n-k}\frac{(2k-2)!!}{(2k-3)!!} \\ &= \frac{n!(2n-4)!}{2^{n-1}\cdot(2n-3)!!(n-2)!(n-1)!} \\ &\qquad \cdot\left(\frac{(2n-2)!!}{n\cdot(2n-5)!!}-\frac{3-2n}{2n}\left(2H_{2n-2}-H_{n-1}-2\right)\right) \\ &= \frac{1}{2}\cdot\frac{(2n-2)!!}{(2n-3)!!}+\frac{1}{4}\left(2H_{2n-2}-H_{n-1}-2\right) \end{aligned} $$

as we claimed. □

Now we can proceed with the proof of Proposition 6.

Proposition 6 *The solution X_n of the equation*

$$ X_n = 2\sum_{k=1}^{n-1} C_{k,n-k}X_k + \sum_{l=1}^{r} a_l\binom{n}{l} + \frac{(2n-2)!!}{(2n-3)!!}\sum_{l=1}^{s} b_l\binom{n}{l} $$

(where $r, s \geqslant 1$ *and* $a_1, \ldots, a_r, b_1, \ldots, b_s \in \mathbb{R}$*) with given initial condition* X_1 *is*

$$X_n = \sum_{l=1}^{s+1} \widehat{a}_l \binom{n}{l} + \frac{(2n-2)!!}{(2n-3)!!} \sum_{l=1}^{r} \widehat{b}_l \binom{n}{l}$$

with

$$\begin{aligned}
\widehat{a}_1 &= X_1 - a_1 \\
\widehat{a}_l &= \frac{l \cdot (2l-2)!!}{(2l-3)!!} \Big(\frac{b_l}{l} + \frac{b_{l-1}}{l-1} \Big), \quad l = 2, \ldots, s \\
\widehat{a}_{s+1} &= \frac{(s+1) \cdot (2s)!!}{s \cdot (2s-1)!!} \cdot b_s \\
\widehat{b}_l &= \frac{(2l-3)!!}{(2l-2)!!} \cdot a_l, \quad l = 1, \ldots, r
\end{aligned}$$

Proof Consider a sequence of the form

$$X_n = \sum_{l=1}^{s+1} \widehat{a}_l \binom{n}{l} + \frac{(2n-2)!!}{(2n-3)!!} \sum_{l=1}^{r} \widehat{b}_l \binom{n}{l}$$

with $\widehat{a}_1, \ldots, \widehat{a}_{s+1}, \widehat{b}_1, \ldots, \widehat{b}_r \in \mathbb{R}$. Then

$$\begin{aligned}
X_n &- 2 \sum_{k=1}^{n-1} C_{k,n-k} X_k \\
&= \sum_{l=1}^{s+1} \widehat{a}_l \binom{n}{l} + \sum_{r=1}^{r} \widehat{b}_l \binom{n}{l} \frac{(2n-2)!!}{(2n-3)!!} \\
&\quad - 2 \sum_{k=1}^{n-1} C_{k,n-k} \left(\sum_{l=1}^{s+1} \widehat{a}_l \binom{k}{l} + \sum_{l=1}^{r} \widehat{b}_l \binom{k}{l} \frac{(2k-2)!!}{(2k-3)!!} \right) \\
&= \sum_{l=1}^{s+1} \widehat{a}_l \left(\binom{n}{l} - 2 \sum_{k=1}^{n-1} C_{k,n-k} \binom{k}{l} \right) \\
&\quad + \sum_{l=1}^{r} \widehat{b}_l \left(\binom{n}{l} \frac{(2n-2)!!}{(2n-3)!!} - 2 \sum_{k=1}^{n-1} C_{k,n-k} \binom{k}{l} \frac{(2k-2)!!}{(2k-3)!!} \right) \\
&= \sum_{l=1}^{s+1} \widehat{a}_l \cdot \frac{l-1}{n-1} \cdot \frac{(2l-3)!!}{(2l-2)!!} \binom{n}{l} \frac{(2n-2)!!}{(2n-3)!!} + \sum_{l=1}^{r} \widehat{b}_l \binom{n}{l} \frac{(2l-2)!!}{(2l-3)!!}
\end{aligned}$$

by Lemmas 3.(b) and 4.(b). The fact that X_n satisfies the recurrence equation in the statement is then equivalent to

$$\begin{aligned}
\sum_{l=1}^{r} \widehat{b}_l \cdot \frac{(2l-2)!!}{(2l-3)!!} \cdot \binom{n}{l} + \frac{(2n-2)!!}{(2n-3)!!} \sum_{l=1}^{s+1} \frac{(l-1) \cdot (2l-3)!!}{(2l-2)!!} \cdot \widehat{a}_l \binom{n}{l} \frac{1}{n-1} \\
= \sum_{l=1}^{r} a_l \binom{n}{l} + \frac{(2n-2)!!}{(2n-3)!!} \sum_{l=1}^{s} b_l \binom{n}{l}.
\end{aligned}$$

This equality will be satisfied if the coefficients $\widehat{a}_1,\dots,\widehat{a}_{s+1},\widehat{b}_1,\dots,\widehat{b}_r$ satisfy that

$$\sum_{l=1}^{r}\widehat{b}_l\cdot\frac{(2l-2)!!}{(2l-3)!!}\binom{n}{l}=\sum_{l=1}^{r}a_l\binom{n}{l} \tag{29}$$

$$\sum_{l=1}^{s+1}\frac{(l-1)\cdot(2l-3)!!}{(2l-2)!!}\cdot\widehat{a}_l\binom{n}{l}\frac{1}{n-1}=\sum_{l=1}^{s}b_l\binom{n}{l} \tag{30}$$

Now, (29) is clearly satisfied if

$$\widehat{b}_l=\frac{(2l-3)!!}{(2l-2)!!}\cdot a_l,\quad l=1,\dots,r.$$

As to (30), it is easy to check that, if $l\geqslant 1$,

$$\binom{n}{l}\frac{1}{n-1}=\frac{1}{l}\binom{n}{l-1}-\frac{l-2}{l}\binom{n}{l-1}\frac{1}{n-1} \tag{31}$$

which implies, by induction on l, that, for every $l\geqslant 2$,

$$\binom{n}{l}\frac{1}{n-1}=\sum_{j=1}^{l-1}(-1)^{j+1}\frac{(l-j)}{l(l-1)}\cdot\binom{n}{l-j}. \tag{32}$$

Indeed, the base case $l=2$ is a direct consequence of (31), and, as to the inductive step, if for a given $l\geqslant 3$ we assume that

$$\binom{n}{l-1}\frac{1}{n-1}=\sum_{j=1}^{l-2}(-1)^{j+1}\frac{(l-1-j)}{(l-1)(l-2)}\cdot\binom{n}{l-1-j},$$

then

$$\begin{aligned}\binom{n}{l}\frac{1}{n-1}&=\frac{1}{l}\binom{n}{l-1}-\frac{l-2}{l}\binom{n}{l-1}\frac{1}{n-1}\quad\text{(by (31))}\\&=\frac{1}{l}\binom{n}{l-1}-\frac{l-2}{l}\sum_{j=1}^{l-2}(-1)^{j+1}\frac{(l-1-j)}{(l-1)(l-2)}\cdot\binom{n}{l-1-j}\\&=\frac{l-1}{l(l-1)}\binom{n}{l-1}-\sum_{j=1}^{l-2}(-1)^{j+1}\frac{(l-1-j)}{l(l-1)}\cdot\binom{n}{l-1-j}\\&=\frac{l-1}{l(l-1)}\binom{n}{l-1}+\sum_{j=2}^{l-1}(-1)^{j+1}\frac{(l-j)}{l(l-1)}\cdot\binom{n}{l-j}\\&=\sum_{j=1}^{l-1}(-1)^{j+1}\frac{(l-j)}{l(l-1)}\cdot\binom{n}{l-j}\end{aligned}$$

Therefore, returning back to (30), using (32) we have that

$$\begin{aligned}
\sum_{l=1}^{s+1} \frac{(l-1)\cdot(2l-3)!!}{(2l-2)!!}\cdot \widehat{a}_l \binom{n}{l}\frac{1}{n-1} &= \sum_{l=2}^{s+1} \frac{(l-1)\cdot(2l-3)!!}{(2l-2)!!}\cdot \widehat{a}_l \binom{n}{l}\frac{1}{n-1}\\
&= \sum_{l=2}^{s+1}\left(\frac{(l-1)\cdot(2l-3)!!}{(2l-2)!!}\cdot \widehat{a}_l \sum_{j=1}^{l-1}(-1)^{j+1}\frac{(l-j)}{l(l-1)}\cdot\binom{n}{l-j}\right)\\
&= \sum_{l=2}^{s+1}\left(\frac{(2l-3)!!}{l\cdot(2l-2)!!}\cdot \widehat{a}_l \sum_{h=1}^{l-1}(-1)^{l-h+1}h\binom{n}{h}\right)\\
&= \sum_{h=1}^{s}\left(\sum_{l=h+1}^{s+1}(-1)^{l-h+1}\frac{h\cdot(2l-3)!!}{l\cdot(2l-2)!!}\cdot \widehat{a}_l\right)\binom{n}{h}
\end{aligned}$$

and thus, (30) is satisfied if

$$\sum_{l=h+1}^{s+1}(-1)^{l-h+1}\frac{h\cdot(2l-3)!!}{l\cdot(2l-2)!!}\cdot \widehat{a}_l = b_h, \quad h=1,\ldots,s.$$

This system of linear equations in $\widehat{a}_2,\ldots,\widehat{a}_{s+1}$ is non singular, and its only solution satisfies

$$\begin{aligned}
\widehat{a}_{s+1} &= \frac{(s+1)\cdot(2s)!!}{s\cdot(2s-1)!!}\cdot b_s\\
\widehat{a}_h &= \frac{h\cdot(2h-2)!!}{(h-1)\cdot(2h-3)!!}\cdot b_{h-1}\\
&\qquad - \sum_{l=h+1}^{s+1}(-1)^{l-h}\frac{h\cdot(2h-2)!!(2l-3)!!}{l\cdot(2l-2)!!(2h-3)!!}\cdot \widehat{a}_l\\
&= \frac{h\cdot(2h-2)!!}{(2h-3)!!}\left(\frac{b_{h-1}}{h-1} - \sum_{l=h+1}^{s+1}\frac{(-1)^{l-h}(2l-3)!!}{l\cdot(2l-2)!!}\cdot \widehat{a}_l\right), \quad h=2,\ldots,s
\end{aligned}$$

Now, let

$$\widetilde{a}_l = \frac{(2l-3)!!}{l\cdot(2l-2)!!}\widehat{a}_l.$$

Then, the previous formulas can be rewritten as

$$\widetilde{a}_{s+1} = \frac{b_s}{s} \qquad\qquad \widetilde{a}_l = \frac{b_{l-1}}{l-1} + \sum_{h=l+1}^{s+1}(-1)^{h-l-1}\widetilde{a}_h, \quad l=2,\ldots,s$$

and the solution of the last recurrence is

$$\widetilde{a}_l = \frac{b_{l-1}}{l-1} + \frac{b_l}{l}. \tag{33}$$

Indeed:

$$\widetilde{a}_s = \frac{b_{s-1}}{s-1} + \widetilde{a}_{s+1} = \frac{b_{s-1}}{s-1} + \frac{b_s}{s}$$

and if (33) holds for every $h = l+1, \dots, s+1$, then

$$\widetilde{a}_l = \frac{b_{l-1}}{l-1} + \sum_{h=l+1}^{s} (-1)^{h-l-1}\Big(\frac{b_{h-1}}{h-1} + \frac{b_h}{h}\Big) + (-1)^{s-l}\cdot\frac{b_s}{s} = \frac{b_{l-1}}{l-1} + \frac{b_l}{l}.$$

Then, finally, for every $l = 2, \dots, s$,

$$\widehat{a}_l = \frac{l\cdot(2l-2)!!}{(2l-3)!!}\widetilde{a}_l = \frac{l\cdot(2l-2)!!}{(2l-3)!!}\Big(\frac{b_{l-1}}{l-1} + \frac{b_l}{l}\Big)$$

as we claimed.

Finally, $\widehat{a}_1$ is obtained by imposing the initial condition

$$\begin{aligned} X_1 &= \sum_{l=1}^{s+1}\widehat{a}_l\binom{1}{l} + \frac{(2-2)!!}{(2-3)!!}\sum_{l=1}^{r}\widehat{b}_l\binom{1}{l} = \widehat{a}_1 + \widehat{b}_1 \\ &\Rightarrow \widehat{a}_1 = X_1 - \widehat{b}_1 = X_1 - \frac{(2-3)!!}{(2-2)!!}\cdot a_1 = X_1 - a_1. \end{aligned}$$

□

SN-5 Proof of Theorem 1

We start by proving a series of lemmas describing the behaviour of V when we remove a deepest leaf from a rooted tree. To simplify the notations, we shall set henceforth

$$W(T) := |L(T)|\cdot V(T) = \sum_{x\in L(T)} (\delta_T(x) - \widehat{S}(T))^2.$$

Moreover, given a tree $T \in \mathcal{T}_n$, we shall denote by $x_1, \dots, x_n$ its leaves ordered in non-decreasing order of depth and we shall set $d_i := \delta_T(x_i)$, for $i = 1, \dots, n$, so that the elements of $\Delta(T)$ are

$$d_1 \leqslant d_2 \leqslant \cdots \leqslant d_{n-2} \leqslant d_{n-1} \leqslant d_n = \delta(T).$$

Since the maximum depth of a tree is reached at least at two sibling leaves, we have that $d_{n-1} = d_n$ and we shall assume without any loss of generality that x_{n-1} and x_n are sibling.

Lemma 5 *Let $T \in \mathcal{T}^*_n$ be a tree with two leaves of maximum depth forming a cherry. Let $T' \in \mathcal{T}^*_{n-1}$ be the tree obtained by removing both leaves in this cherry, so that the root of the cherry becomes a leaf. Then,*

$$W(T') = W(T) - \frac{n}{n-1}(\delta(T) - \widehat{S}(T) + 1)^2 + 2.$$

Proof For simplicity, we shall denote $\delta(T)$ by δ. With the notations on the leaves x_i of T and their depths d_i introduced above, we shall assume without any loss of generality that x_{n-1} and x_n are not only sibling, but they form the cherry in the hypothesis. Let $T' \in \mathcal{T}^*_{n-1}$ be the tree obtained by removing from T these two leaves,

so that their common parent becomes a new leaf of depth $d_{n-1}-1=d_n-1=\delta-1$, which we shall still denote by x_{n-1}; cf. Fig. 9. Thus,

$$\Delta(T')=\{d_1,\ldots,d_{n-2},d_{n-1}-1\}$$

and then

$$\widehat{S}(T')=\frac{\sum_{i=1}^{n-1}d_i-1}{n-1}=\frac{n\widehat{S}(T)-\delta-1}{n-1}=\widehat{S}(T)-\frac{\delta-\widehat{S}(T)+1}{n-1}. \tag{34}$$

Finally,

$$\begin{aligned}
W(T') &= \sum_{j=1}^{n-2}(d_j-\widehat{S}(T'))^2+(d_{n-1}-1-\widehat{S}(T'))^2\\
&= \sum_{j=1}^{n}(d_j-\widehat{S}(T'))^2-2(d_{n-1}-\widehat{S}(T'))+1-(d_n-\widehat{S}(T'))^2\\
&= \sum_{j=1}^{n}(d_j-\widehat{S}(T'))^2-2(\delta-\widehat{S}(T'))+1-(\delta-\widehat{S}(T'))^2
\end{aligned}$$

(using that $d_{n-1}=d_n=\delta$)

$$\begin{aligned}
&= \sum_{j=1}^{n}(d_j-\widehat{S}(T'))^2-(\delta-\widehat{S}(T')+1)^2+2\\
&= \sum_{j=1}^{n}\Big(d_j-\widehat{S}(T)+\frac{\delta-\widehat{S}(T)+1}{n-1}\Big)^2-\Big(\delta-\widehat{S}(T)+\frac{\delta-\widehat{S}(T)+1}{n-1}+1\Big)^2+2
\end{aligned}$$

(using (34))

$$\begin{aligned}
&= \sum_{j=1}^{n}(d_j-\widehat{S}(T))^2+2\Big(\frac{\delta-\widehat{S}(T)+1}{n-1}\Big)\sum_{j=1}^{n}(d_j-\widehat{S}(T))\\
&\quad +n\Big(\frac{\delta-\widehat{S}(T)+1}{n-1}\Big)^2-\Big(\frac{n(\delta-\widehat{S}(T)+1)}{n-1}\Big)^2+2\\
&= W(T)-\frac{n}{n-1}(\delta-\widehat{S}(T)+1)^2+2
\end{aligned}$$

(because $\sum_{j=1}^{n}(d_j-\widehat{S}(T))=0$) as we claimed. □

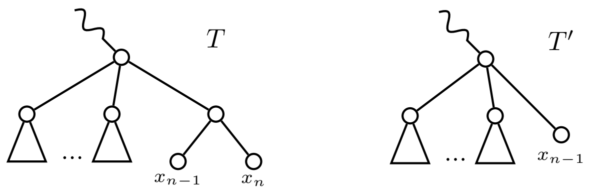

Figure 9 A tree T with a cherry at the bottom and the tree T' obtained by removing this cherry.

Lemma 6 *Let $T\in\mathcal{T}^*_n$ be a tree with k leaves of maximum depth forming a k-fan with $k\geqslant 3$. Let $T'\in\mathcal{T}^*_{n-1}$ be the tree obtained by removing one leaf from this k-fan.*

Then,

$$W(T') = W(T) - \frac{n}{n-1}(\delta(T) - \widehat{S}(T))^2.$$

Proof We shall denote again $\delta(T)$ by δ and we shall use again the notations on the leaves x_i of T and their depths d_i introduced at the beginning of this section. We shall assume without any loss of generality that x_n belongs to the k-fan in the hypothesis, and that this is the leaf we remove to obtain T'. In this way, since the remaining leaves have the same depths in T and in T',

$$\Delta(T') = \{d_1, \ldots, d_{n-2}, d_{n-1}\}.$$

Then

$$\widehat{S}(T') = \frac{\sum_{i=1}^{n} d_i - d_n}{n-1} = \frac{n\widehat{S}(T) - \delta}{n-1} = \widehat{S}(T) - \frac{\delta - \widehat{S}(T)}{n-1}$$

and hence, computing $W(T')$ in terms of $W(T)$ as in the proof of the previous lemma, we obtain

$$\begin{aligned} W(T') &= \sum_{j=1}^{n-1}(d_j - \widehat{S}(T'))^2 = \sum_{j=1}^{n}(d_j - \widehat{S}(T'))^2 - (d_n - \widehat{S}(T'))^2 \\ &= \sum_{j=1}^{n}\Big(d_j - \widehat{S}(T) + \frac{\delta - \widehat{S}(T)}{n-1}\Big)^2 - \Big(\delta - \widehat{S}(T) + \frac{\delta - \widehat{S}(T)}{n-1}\Big)^2 \\ &= \sum_{j=1}^{n}(d_j - \widehat{S}(T))^2 + 2\Big(\frac{\delta - \widehat{S}(T)}{n-1}\Big)\sum_{j=1}^{n}(d_j - \widehat{S}(T)) + n\Big(\frac{\delta - \widehat{S}(T)}{n-1}\Big)^2 \\ &\qquad -\Big(\frac{n(\delta - \widehat{S}(T))}{n-1}\Big)^2 \\ &= W(T) - \frac{n}{n-1}(\delta - \widehat{S}(T))^2 \end{aligned}$$

as we claimed. □

Lemma 7 *Let $T \in \mathcal{T}_n^*$ be a tree with two leaves of maximum depth forming a cherry. Then,*

$$\delta(T) - \widehat{S}(T) \leqslant \frac{(n-1)(n-2)}{2n}$$

and the equality holds only when $T = K_n$.

Proof First of all, since (cf. Example 1 in the main text)

$$\delta(K_n) = n-1 \quad \text{and} \quad \widehat{S}(K_n) = \frac{(n-1)(n+2)}{2n},$$

we have that $\delta(K_n) - \widehat{S}(K_n) = (n-1)(n-2)/(2n)$, for every n, and therefore the inequality in the statement is an equality for combs.

Now, we shall prove the statement by induction on n. The cases when $n = 1$ or $n = 2$ are obvious, because then $\mathcal{T}_n^* = \{K_n\}$, and the case when $n = 3$ is also obvious, because the only tree in $\mathcal{T}_3^*$ with two leaves of maximum depth forming

a cherry is K_3. Let us prove now the inductive step when $n \geqslant 4$. To simplify the notations, for every tree T we shall set

$$\Psi(T) \coloneqq \delta(T) - \widehat{S}(T) + 1.$$

Let $T \in \mathcal{T}^*_n$, with $n \geqslant 4$, be a tree with two leaves of maximum depth forming a cherry. We use again the notations on the leaves x_i of T and their depths d_i introduced at the beginning of this section, and we assume that the cherry at maximum depth is formed by the leaves x_{n-1} and x_n. Let $T' \in \mathcal{T}^*_{n-1}$ be the tree obtained from T by removing this cherry and replacing it by its root, that becomes a leaf which we shall still denote by x_{n-1}; cf. again Fig. 9. On the one hand, we have that $\delta(T') = \delta(T) - 1$ if T contains only the leaves x_{n-1}, x_n at depth $\delta(T)$, and $\delta(T') = \delta(T)$ if T contains leaves of depth $\delta(T)$ other than x_{n-1} and x_n. On the other hand, by the expression (34) for $\widehat{S}(T')$ obtained in the proof of Lemma 5, we have that

$$\delta(T) - \widehat{S}(T') + 1 = \delta(T) - \widehat{S}(T) + \frac{\delta(T) - \widehat{S}(T) + 1}{n-1} + 1 = \frac{n}{n-1}\Psi(T).$$

Combining these two facts we obtain that

$$\begin{aligned}\Psi(T) &= \frac{n-1}{n}(\delta(T) - \widehat{S}(T') + 1)\\ &\leqslant \frac{n-1}{n}(\delta(T') + 1 - \widehat{S}(T') + 1) = \frac{n-1}{n}(\Psi(T') + 1)\end{aligned}$$

and the equality holds only when T contains no other leaf of depth $\delta(T)$ than x_{n-1} and x_n.

Now we must distinguish two cases:

(a) If T' contains again a cherry at maximum depth, we can apply the induction hypothesis and we obtain that

$$\Psi(T') = \delta(T') - \widehat{S}(T') + 1 \leqslant \frac{(n-2)(n-3)}{2(n-1)} + 1,$$

with the equality holding only if $T' = K_{n-1}$. Then,

$$\begin{aligned}\delta(T) - \widehat{S}(T) = \Psi(T) - 1 &\leqslant \frac{n-1}{n}(\Psi(T') + 1) - 1\\ &\leqslant \frac{n-1}{n}\Big(\frac{(n-2)(n-3)}{2(n-1)} + 2\Big) - 1 = \frac{(n-1)(n-2)}{2n}\end{aligned}$$

and the equality holds only if $T' = K_{n-1}$ and T is obtained by replacing a leaf of largest depth by a cherry, that is, when $T = K_n$.

(b) Assume now that T' has no cherry at maximum depth, and in particular that x_{n-1} belongs to a k-fan with $k \geqslant 3$. Without any loss of generality, assume that this fan is $x_{n-k}, \ldots, x_{n-1}$ and let y be their common parent. In this case, let $T'' \in \mathcal{T}^*_{n-1}$ be the tree obtained from T' by adding a new node z and replacing the arcs $(y, x_{n-2}), (y, x_{n-1})$ by new arcs $(y, z), (z, x_{n-2}), (z, x_{n-1})$; see Fig. 10.

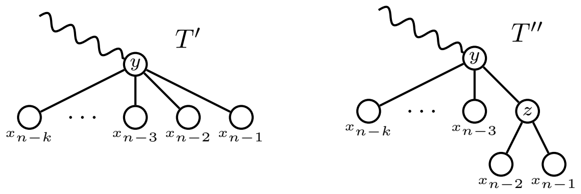

Figure 10 The trees T' and T'' in the proof of case (b) of Lemma 7.

The tree T'' obtained in this way has the cherry (x_{n-2}, x_{n-1}) at maximum depth $\delta(T'') = \delta(T')+1$, and, since these two leaves increase their depths in T'' in one unit with respect to T' and the other leaves in T' maintain their depths in T'', we have that $\widehat{S}(T'') = \widehat{S}(T') + 2/(n-1)$. Thus,

$$\begin{aligned}\Psi(T'') &= \delta(T'') - \widehat{S}(T'') + 1 = \delta(T') + 1 - \widehat{S}(T') - \frac{2}{n-1} + 1\\ &= \Psi(T') + \frac{n-3}{n-1} > \Psi(T')\end{aligned}$$

(because we are assuming $n \geqslant 4$) and hence

$$\Psi(T) \leqslant \frac{n-1}{n}(\Psi(T')+1) < \frac{n-1}{n}(\Psi(T'')+1)$$

where now $T'' \in \mathcal{T}^*_{n-1}$ has a cherry at maximum depth and therefore we can apply to it the induction hypothesis:

$$\delta(T'') - \widehat{S}(T'') \leqslant \frac{(n-2)(n-3)}{2(n-1)}.$$

We can now proceed as in the last step in (a):

$$\begin{aligned}\delta(T) - \widehat{S}(T) = \Psi(T) - 1 &< \frac{n-1}{n}(\Psi(T'')+1) - 1\\ &\leqslant \frac{n-1}{n}\Big(\frac{(n-2)(n-3)}{2(n-1)} + 2\Big) - 1 = \frac{(n-1)(n-2)}{2n}\end{aligned}$$

Let us emphasize that in this case T can never be a comb, and the inequality in the statement is strict.

This completes the proof of the statement by induction on n. □

We are now in position to prove Theorem 1.

Theorem 1 *The maximum value of V on $\mathcal{T}^*_n$ is always reached exactly at the combs K_n.*

Proof Since, for any fixed n, the maximum values on $\mathcal{T}^*_n$ of V and W are reached at the same trees, it will be enough to prove, by induction on n, the following property

for every $T \in \mathcal{T}_n^*$:

$$W(T) \leqslant W(K_n), \textit{ and the equality holds if, and only if, } T = K_n. \tag{35}$$

The cases when $n = 1$ or $n = 2$ are obvious, because $\mathcal{T}_1^* = \{K_1\}$ and $\mathcal{T}_2^* = \{K_2\}$. So, we assume henceforth that $n \geqslant 3$ and that assertion (35) is true for $n - 1$.

Let $T \in \mathcal{T}_n^*$. With the notations on the leaves x_i of T and their depths d_i introduced at the beginning of this section, we must distinguish two cases, depending on whether the leaf x_n has only one sibling, forming a cherry, or more than one sibling. We start with this last case.

(A) Let us assume that x_n belongs to a k-fan, with $k \geqslant 3$; without any loss of generality we consider that the leaves in this fan are $x_{n-k+1}, \dots, x_{n-1}, x_n$. Let y be the common parent of this fan. Let $T' \in \mathcal{T}_n^*$ be the tree obtained from T by adding a new node z and replacing the arcs $(y, x_{n-1}), (y, x_n)$ by new arcs $(y, z), (z, x_{n-1}), (z, x_n)$. In this way, the leaves $x_1, \dots, x_{n-2}$ in T' have the same depths as in T and the depths of x_{n-1} and x_n in T' are their depths in T plus 1, and therefore $\delta(T') = \delta(T) + 1$ and $\widehat{S}(T') = \widehat{S}(T) + 2/n$.

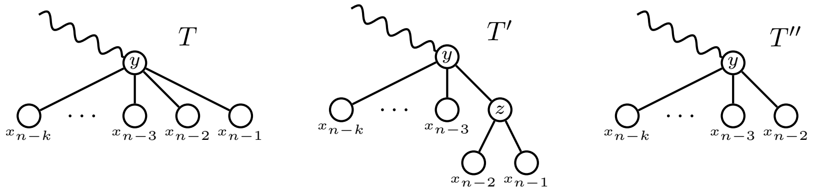

Figure 11 The trees T, T', and T'' in the proof of case (a) of Theorem 1.

Now, let $T'' \in \mathcal{T}_{n-1}^*$ be the tree obtained from T by removing from it the leaf x_n. It is obvious that T'' can also be understood as the tree obtained from T' by removing the cherry (x_{n-1}, x_n) and replacing it by a leaf x_{n-1}. Therefore, by Lemmas 5 and 6

$$W(T'') = W(T') - \frac{n}{n-1}(\delta(T') - \widehat{S}(T') + 1)^2 + 2$$
$$W(T'') = W(T) - \frac{n}{n-1}(\delta(T) - \widehat{S}(T))^2$$

from where we obtain that

$$\begin{aligned} W(T) &= W(T') - \frac{n}{n-1}(\delta(T') - \widehat{S}(T') + 1)^2 + 2 + \frac{n}{n-1}(\delta(T) - \widehat{S}(T))^2 \\ &= W(T') + 2 - \frac{n}{n-1}\Big(\delta(T) - \widehat{S}(T) - \frac{2}{n} + 2\Big)^2 + \frac{n}{n-1}(\delta(T) - \widehat{S}(T))^2 \\ &= W(T') + 2 - 4\Big(\delta(T) - \widehat{S}(T) + \frac{n-1}{n}\Big) < W(T') \end{aligned}$$

where this last inequality holds because $\delta(T) \geqslant \widehat{S}(T)$ and, by assumption, $n \geqslant 3$.

Therefore, if x_n belongs to a k-fan, for some $k \geqslant 3$, there exists a tree $T' \in \mathcal{T}^*_n$ with larger W value that contains a leaf of maximum depth belonging to a cherry. This is the case we consider next.

(B) Assume now that x_{n-1} and x_n form a cherry. Let $T' \in \mathcal{T}^*_{n-1}$ be the tree obtained by removing from T this cherry, leaving its root as a leaf. Then:

$$\begin{aligned}
&W(T) = W(T') + \frac{n}{n-1}(\delta(T) - \widehat{S}(T) + 1)^2 - 2\\
&\text{(by Lemma 5)}\\
&\quad \leqslant W(T') + \frac{n}{n-1}\Big(\frac{(n-1)(n-2)}{2n} + 1\Big)^2 - 2\\
&\text{(by Lemma 7)}\\
&\quad \leqslant W(K_{n-1}) + \frac{n}{n-1}\Big(\frac{(n-1)(n-2)}{2n} + 1\Big)^2 - 2\\
&\text{(by the induction hypothesis)}\\
&\quad = W(K_{n-1}) + \frac{n}{n-1}\Big(\delta(K_n) - \widehat{S}(K_n) + 1\Big)^2 - 2 = W(K_n)\\
&\text{(again by Lemma 5).}
\end{aligned}$$

This proves that, when T has a cherry at the bottom, $W(T) \leqslant W(K_n)$. Moreover, the equality holds only when both intermediate inequalities are equalities, that is (by Lemma 7 and the induction hypothesis) exactly when $T = K_n$.

In summary, we have proved that if $T \in \mathcal{T}^*_n$ has some cherry at maximum depth, then $W(T) \leqslant W(K_n)$, with the equality holding only when $T = K_n$, and that if $T \in \mathcal{T}^*_n$ does not have any cherry at maximum depth, then there exists a tree $T_0 \in \mathcal{T}^*_n$ with a cherry at maximum depth such that $W(T) < W(T_0) \leqslant W(K_n)$. This completes the proof by induction of property (35). □

SN-6 Proof of Theorem 2

The goal of this section is to prove the necessary condition on the bifurcating trees with minimum V value stated in the following result.

Theorem 2 *If $T \in \mathcal{BT}^*_n$ has the minimum value of V, then it is of some type $T_{n;l_1,\dots,l_j}$ with $5 \leqslant l_1 < \cdots < l_j \leqslant \delta(T) - 2$.*

Now, since each one of $\mathcal{BT}^*_1$, $\mathcal{BT}^*_2$, and $\mathcal{BT}^*_3$ contains only one tree, it is enough to consider the case $n \geqslant 4$. Moreover, the tree in $\mathcal{BT}^*_4$ with minimum V value is clearly B_4, because when n is a power of 2, B_n is fully symmetric and hence $V(B_n) = 0$ is minimum in $\mathcal{BT}^*_n$. Therefore, we can restrict ourselves to the case $n \geqslant 5$.

Lemma 8 *Let $n \geqslant 5$. If $T \in \mathcal{BT}^*_n$ has a leaf of depth 1, then $V(T)$ is not minimum in $\mathcal{BT}^*_n$.*

Proof Let $T \in \mathcal{BT}^*_n$ be a tree with a leaf of depth 1, so that it has the form $T = B_1 \star T_0$ with B_1 a tree with a single leaf and $T_0 \in \mathcal{BT}^*_{n-1}$. Let $T' \in \mathcal{BT}^*_n$ be the tree obtained from T_0 by replacing a leaf of smallest depth in it by a cherry (of depth one unit larger). So, if the depths of the leaves in T are $1 < d_2 \leqslant d_3 \leqslant \cdots \leqslant d_n$,

the depths of the leaves in T_0 are $d_2-1 \leqslant d_3-1 \leqslant \cdots \leqslant d_n-1$, and the depths of the leaves in T' are then $d_2, d_2, d_3-1, \ldots, d_n-1$. Then,

$$\begin{aligned}\widehat{S}(T') &= \frac{\sum_{i=2}^n d_i + d_2 - (n-2)}{n} = \frac{\left(1+\sum_{i=2}^n d_i\right) + d_2 - n + 1}{n}\\ &= \frac{n\widehat{S}(T) + d_2 - n + 1}{n} = \widehat{S}(T) - 1 + \frac{d_2+1}{n}\end{aligned}$$

and

$$\begin{aligned}W(T') &= 2(d_2 - \widehat{S}(T'))^2 + \sum_{j=3}^n (d_j - 1 - \widehat{S}(T'))^2\\ &= 2\Big(d_2 - \widehat{S}(T) + 1 - \frac{d_2+1}{n}\Big)^2 + \sum_{j=3}^n \Big(d_j - \widehat{S}(T) - \frac{d_2+1}{n}\Big)^2\\ &= 2(d_2 - \widehat{S}(T))^2 + 4(d_2 - \widehat{S}(T))\Big(1 - \frac{d_2+1}{n}\Big) + 2\Big(1 - \frac{d_2+1}{n}\Big)^2\\ &\quad + \sum_{j=3}^n (d_j - \widehat{S}(T))^2 - 2\Big(\frac{d_2+1}{n}\Big)\sum_{j=3}^n (d_j - \widehat{S}(T)) + (n-2)\Big(\frac{d_2+1}{n}\Big)^2\\ &= W(T) + (d_2 - \widehat{S}(T))^2 - (1 - \widehat{S}(T))^2 + 4(d_2 - \widehat{S}(T))\\ &\quad -2(d_2 - \widehat{S}(T))\Big(\frac{d_2+1}{n}\Big) - 2\Big(\frac{d_2+1}{n}\Big)\sum_{j=2}^n (d_j - \widehat{S}(T))\\ &\quad +2\Big(1 - \frac{d_2+1}{n}\Big)^2 + (n-2)\Big(\frac{d_2+1}{n}\Big)^2\\ &\text{(because } W(T) = (1-\widehat{S}(T))^2 + \textstyle\sum_{j=2}^n (d_j - \widehat{S}(T))^2)\\ &= W(T) + (d_2 - \widehat{S}(T) + 2)^2 - 4 - (1 - \widehat{S}(T))^2\\ &\quad -2(d_2 - \widehat{S}(T))\Big(\frac{d_2+1}{n}\Big) + 2\Big(\frac{d_2+1}{n}\Big)(1 - \widehat{S}(T))\\ &\quad +2 - 4\Big(\frac{d_2+1}{n}\Big) + n\Big(\frac{d_2+1}{n}\Big)^2\\ &\text{(using that } 1 + \textstyle\sum_{i=2}^n d_i = n\widehat{S}(T))\\ &= W(T) + (d_2 - \widehat{S}(T) + 2)^2 - (1 - \widehat{S}(T))^2 - 2 - \frac{(d_2+1)^2}{n}\\ &= W(T) + (d_2+1)\Big(d_2 + 3 - 2\widehat{S}(T) - \frac{d_2+1}{n}\Big) - 2\end{aligned}$$

Then, if

$$(d_2+1)\Big(d_2 + 3 - 2\widehat{S}(T) - \frac{d_2+1}{n}\Big) - 2 < 0 \tag{36}$$

we have that $W(T') < W(T)$ and therefore $V(T)$ cannot be minimum in $\mathcal{BT}_n^*$. Let us check now that this inequality always holds if $n \geqslant 5$. To do that, we rephrase it in terms of T_0.

To begin with,

$$\widehat{S}(T) = \frac{1 + \sum_{i=2}^n d_i}{n} = \frac{n + \sum_{i=2}^n (d_i - 1)}{n} = 1 + \frac{(n-1)\widehat{S}(T_0)}{n}.$$

Then

$$
\begin{aligned}
&(d_2+1)\Big(d_2+3-2\widehat{S}(T)-\frac{d_2+1}{n}\Big)-2\\
&\quad=(d_2+1)\Big(d_2+1-\frac{2(n-1)\widehat{S}(T_0)}{n}-\frac{d_2+1}{n}\Big)-2\\
&\quad=\frac{n-1}{n}(d_2+1)(d_2+1-2\widehat{S}(T_0))-2
\end{aligned}
$$

Now, since $\widehat{S}(T_0)\geqslant d_2-1$, because d_2-1 is the smallest depth of a leaf in T_0, if $\widehat{S}(T_0)\geqslant 2$ then we guarantee that inequality (36) holds. But, since T_0 has at least $n-1\geqslant 4$ leaves, all its leaves but at most two of them have depth at least 3, and if it contains two leaves of depth smaller than 3, they have depths 1 and 2 or depth 2 both of them. Therefore,

$$
\widehat{S}(T_0)\geqslant\frac{1+2+3(n-3)}{n-1}=\frac{3n-6}{n-1}>2\quad\text{if } n\geqslant 5.
$$

Therefore, inequality (36) holds and hence $V(T)$ is not minimum in $\mathcal{BT}^*_n$, as we claimed. □

Lemma 9 *Let $T\in\mathcal{BT}^*_n$ be a bifurcating tree containing a leaf of depth $d<\delta(T)$. Let $T'_d\in\mathcal{BT}^*_n$ be the tree obtained by removing a cherry of depth $\delta(T)$ and replacing a leaf of depth d by a cherry of depth $d+1$. Then,*

$$
W(T'_d)=W(T)-\Big(\frac{\delta(T)-d-1}{n}\Big)\big(n(\delta(T)+d+3-2\widehat{S}(T))+\delta(T)-d-1\big)
$$

Proof Let $T\in\mathcal{BT}^*_n$. With the notations on the leaves x_i of T and their depths d_i introduced at the beginning of the previous section, we shall assume without any loss of generality that $d=d_i$ and that the pair of leaves of depth $\delta:=\delta(T)$ that are removed from T are x_{n-1},x_n. Therefore

$$
\Delta(T'_d)=\{d_1,\ldots,d_{i-1},d_i+1,d_i+1,d_{i+1},\ldots,d_{n-2},d_{n-1}-1\}.
$$

Then

$$
\begin{aligned}
\widehat{S}(T'_d)\ &=\frac{\sum_{j=1}^{n-2}d_j+d_i+2+d_{n-1}-1}{n}=\frac{\sum_{j=1}^{n}d_j+d_i+1-d_n}{n}\\
&=\widehat{S}(T)-\frac{d_n-d_i-1}{n}=\widehat{S}(T)-\frac{\delta-d-1}{n}
\end{aligned}
\tag{37}
$$

and

$$
\begin{aligned}
W(T'_d) &= \sum_{\substack{j=1,\dots,n-2\\ j\neq i}} (d_j-\widehat{S}(T'_d))^2 + 2(d_i+1-\widehat{S}(T'_d))^2 + (d_{n-1}-1-\widehat{S}(T'_d))^2\\
&= \sum_{j=1}^{n}(d_j-\widehat{S}(T'_d))^2 + (d_i+1-\widehat{S}(T'_d))^2 - (d_n-\widehat{S}(T'_d))^2\\
&\qquad +2(d_i-\widehat{S}(T'_d))+1-2(d_{n-1}-\widehat{S}(T'_d))+1\\
&= \sum_{j=1}^{n}(d_j-\widehat{S}(T'_d))^2 + (d+2-\widehat{S}(T'_d))^2 - (\delta+1-\widehat{S}(T'_d))^2\\
&\text{(using that } d_{n-1}=d_n=\delta \text{ and } d_i=d)\\
&= \sum_{j=1}^{n}(d_j-\widehat{S}(T'_d))^2 - (\delta+d+3-2\widehat{S}(T'_d))(\delta-d-1)\\
&= \sum_{j=1}^{n}\Big(d_j-\widehat{S}(T)+\frac{\delta-d-1}{n}\Big)^2\\
&\qquad -\Big(\delta+d+3-2\widehat{S}(T)+2\cdot\frac{\delta-d-1}{n}\Big)(\delta-d-1)\\
&= \sum_{j=1}^{n}(d_j-\widehat{S}(T))^2 + 2\Big(\frac{\delta-d-1}{n}\Big)\sum_{j=1}^{n}(d_j-\widehat{S}(T)) + n\Big(\frac{\delta-d-1}{n}\Big)^2\\
&\qquad -(\delta+d+3-2\widehat{S}(T))(\delta-d-1) - 2\Big(\frac{(\delta-d-1)^2}{n}\Big)\\
&= W(T) - \frac{(\delta-d-1)^2}{n} - (\delta-d-1)(\delta+d+3-2\widehat{S}(T))\\
&= W(T) - \Big(\frac{\delta-d-1}{n}\Big)\big(n(\delta+d+3-2\widehat{S}(T))+\delta-d-1\big)
\end{aligned}
$$

as we claimed. □

Corollary 1 *If $T \in \mathcal{BT}^*_n$ has the minimum value of V and it contains some leaf of depth $\delta(T)-l$, with $l>1$, then*

$$l \geqslant 3 + \frac{2(n(\delta(T)-\widehat{S}(T))+1)}{n-1}$$

and in particular T does not contain leaves of depth $\delta(T)-2$ or $\delta(T)-3$.

Proof If $V(T)$, or, equivalently, $W(T)$ is minimum on $\mathcal{BT}^*_n$ and T contains a leaf of depth $d=\delta(T)-l<\delta(T)-1$, then, with the notations of the last lemma, it must happen that

$$(\delta(T)-d-1)\big(n(\delta(T)+d+3-2\widehat{S}(T))+\delta(T)-d-1\big) = n(W(T)-W(T'_d)) \leqslant 0.$$

Since $\delta(T)-d-1=l-1>0$, this is equivalent to

$$n(\delta(T)+d+3-2\widehat{S}(T))+\delta(T)-d-1 \leqslant 0.$$

Replacing in this inequality d by $\delta(T)-l$ and solving for l we finally obtain

$$l \geqslant \frac{2n(\delta(T)-\widehat{S}(T))+3n-1}{n-1} = 3 + \frac{2(n(\delta(T)-\widehat{S}(T))+1)}{n-1} > 3.$$

□

Corollary 2 *Let $T \in \mathcal{BT}_n^*$ be a bifurcating tree that has a cherry of depth $d < \delta(T)$. Let $T_d^* \in \mathcal{BT}_n^*$ be the tree obtained by removing this cherry, leaving in its place a leaf of depth $d-1$, and replacing a leaf of depth $\delta(T)$ by a cherry of depth $\delta(T)+1$. Then,*

$$W(T_d^*) = W(T) + \Big(\frac{\delta(T) - d + 1}{n}\Big)\big(n(\delta(T) + d + 3 - 2\widehat{S}(T)) - (\delta(T) - d + 1)\big).$$

Proof T is obtained from T_d^* by removing a cherry of maximum depth $\delta(T_d^*) = \delta(T)+1$ and replacing a leaf of depth $d-1$ by a cherry of depth d. In other words, with the notations of Lemma 9, $T = (T_d^*)'_{d-1}$. Then, by (37),

$$\widehat{S}(T) = \widehat{S}((T_d^*)'_{d-1}) = \widehat{S}(T_d^*) - \frac{\delta(T_d^*) - (d-1) - 1}{n} = \widehat{S}(T_d^*) - \frac{\delta(T) + 1 - d}{n}$$

and, by Lemma 9,

$$\begin{aligned}
W(T) &= W((T_d^*)'_{d-1}) \\
&= W(T_d^*) - \Big(\frac{\delta(T_d^*) - (d-1) - 1}{n}\Big)\big(n(\delta(T_d^*) + (d-1) + 3 - 2\widehat{S}(T_d^*)) \\
&\qquad + \delta(T_d^*) - (d-1) - 1\big) \\
&= W(T_d^*) - \Big(\frac{\delta(T) + 1 - d}{n}\Big)\Big(n\Big[\delta(T) + 1 + d + 2 \\
&\qquad -2\Big(\widehat{S}(T) + \frac{\delta(T) + 1 - d}{n}\Big)\Big] + \delta(T) + 1 - d\Big) \\
&= W(T_d^*) - \Big(\frac{\delta(T) - d + 1}{n}\Big)\big(n(\delta(T) + d + 3 - 2\widehat{S}(T)) - (\delta(T) - d + 1)\big)
\end{aligned}$$

from where the expression in the statement follows. □

Corollary 3 *If $T \in \mathcal{BT}_n^*$ contains two leaves of the same depth $d < \delta(T) - 1$, then $V(T)$ is not minimum in $\mathcal{BT}_n^*$.*

Proof Let $T \in \mathcal{BT}_n^*$ and assume that it has two leaves, y_0 and y_1, of the same depth $d < \delta(T) - 1$. If $\delta(T) + d + 3 - 2\widehat{S}(T) \geqslant 0$ then, with the notations of Lemma 9,

$$\begin{aligned}
W(T'_d) &= W(T) - (\delta(T) - d - 1)\Big(\delta(T) + d + 3 - 2\widehat{S}(T) + \frac{\delta(T) - d - 1}{n}\Big) \\
&< W(T)
\end{aligned}$$

and therefore $V(T)$ cannot be minimum in $\mathcal{BT}_n^*$.

So, assume that $\delta(T) + d + 3 - 2\widehat{S}(T) < 0$. In this case, if one of the leaves y_0 or y_1 belongs to a cherry, then, with the notations of Corollary 2,

$$\begin{aligned}
W(T_d^*) &= W(T) + (\delta(T) - d + 1)\Big(\delta(T) + d + 3 - 2\widehat{S}(T) - \frac{\delta(T) - d + 1}{n}\Big) \\
&< W(T)
\end{aligned}$$

and therefore in this case $V(T)$ cannot be minimum in $\mathcal{BT}^*_n$, either.

Finally, if y_0 and y_1 do not belong to any cherry, let v_0 and v_1 their respective parents and z_0 and z_1 their respective siblings, which are not leaves. Let T' be obtained from T by interchanging y_1 with z_0: that is, by removing the arcs (v_0, z_0) and (v_1, y_1) and replacing them by arcs (v_0, y_1) and (v_1, z_0) (see Fig. 12). Since $\delta_T(y_1) = \delta_T(y_0) = \delta_T(z_0)$, the resulting tree T' is depth-equivalent to T, and in particular $V(T) = V(T')$. But T' contains the cherry (y_0, y_1) of depth $d < \delta(T) - 1$ and therefore, as we have just seen, $V(T) = V(T')$ cannot minimum in $\mathcal{BT}^*_n$. □

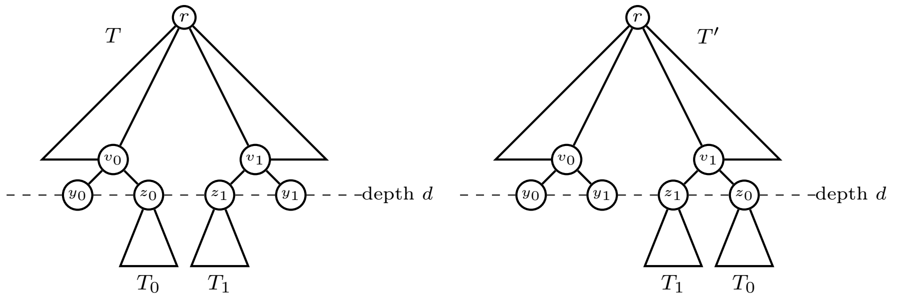

Figure 12 The depth-equivalent trees T and T' appearing in the last paragraph of the proof of Corollary 3.

We can summarize the results obtained so far in the following corollary:

Corollary 4 *If $T \in \mathcal{BT}^*_n$ has the minimum value of V, then it is of some type $T_{n;l_1,\dots,l_j}$ with $4 \leqslant l_1 < \cdots < l_j \leqslant \delta(T) - 2$.*

Next lemma finally completes the proof of Theorem 2.

Lemma 10 *Let T be a tree of type $T_{n;l_1,\dots,l_j}$ with $j \geqslant 1$. If $V(T)$ is minimum in $\mathcal{BT}^*_n$, then $l_1 \geqslant 5$.*

Proof Let T be a tree of type $T_{n;l_1,\dots,l_j}$, with $j \geqslant 1$, such that $V(T)$ is minimum in $\mathcal{BT}^*_n$. By the last corollary, we already know that $4 \leqslant l_1 < \cdots < l_j \leqslant \delta - 2$. Set $\delta := \delta(T)$ and $m := \lfloor \log_2(n) \rfloor$, so that $n = 2^m + k$ with $0 \leqslant k < 2^m$.

In the proof of Lemma 2 we saw that

$$\widehat{S}(T) = \delta - \frac{p_1 + \sum_{i=1}^{j} l_i}{n}.$$

Then, if $p_0 \leqslant n/2$, we have by Corollary 1 that

$$\begin{aligned} l_1 &\geqslant 3 + \frac{2(n(\delta - \widehat{S}(T)) + 1)}{n-1} = 3 + \frac{2(p_1 + \sum_{i=1}^{j} l_i + 1)}{n-1} \\ &> 3 + \frac{2(p_1 + j)}{n-1} = 3 + \frac{2(n - p_0)}{n-1} \geqslant 3 + \frac{n}{n-1} > 4 \end{aligned}$$

which implies that $l_1 \geqslant 5$.

Assume now that $p_0 > n/2$. In this case, we can also assume that $n \geqslant 32$. Indeed, if $n < 32$, then $m \leqslant 4$ and hence, by Lemma 1, $\delta \leqslant 6$. Thus, since $4 \leqslant l_1 \leqslant \delta - 2 \leqslant 4$, it must happen that $\delta = 6$, $m = 4$, $j = 1$, and $l_1 = 4$. So, $n = 2^4 + k$ with $k \leqslant 15$ and since $\delta = 4 + 2$ we are in case (c) in the statement of the aforementioned lemma, and therefore $p_1 = 3 \cdot 2^4 - k - (2^4 - 1) = 33 - k$. Thus,

$$p_0 = n - p_1 - 1 = 2^4 + k - 33 + k - 1 = -18 + 2k \leqslant 8 + \frac{k}{2} = \frac{n}{2}$$

because $k \leqslant 15$, which contradicts the assumption that $p_0 > n/2$.

So, in particular, we can assume that T contains at least 16 leaves of depth δ. Assume that T contains a leaf x of depth $\delta - 4$, so that T is of type $T_{n;4,l_2,\ldots,l_j}$, and let y its sibling, also of depth $\delta - 4$, and z their common parent. Since T does not contain either any other leaf of depth $\delta - 4$ or leaves of depths $\delta - 3$ or $\delta - 2$, the leaves that descend from y have depths $\delta - 1$ or δ. Since T contains at least 16 leaves of depth δ, by pruning and regrafting cherries at maximum depth if necessary, we can assume without any loss of generality that all the leaves that descend from y have depth δ and hence that the subtree of T rooted at y is the fully symmetric tree B_{16}.

Let T' be now the tree obtained from T by, on the one hand, removing the leaf x, its parent z and the three arcs incident to z, and replacing them by an arc from the parent of z to y (which now becomes of depth $\delta - 5$), and, on the other hand, replacing the subtree B_{16} rooted at y by a maximally balanced tree B_{17}; see Fig. 13. From the 17 leaves in T' that are descendant of y, 2 have depth δ in T', and the remaining 15 have depths $\delta - 1$, and the rest of leaves in T' have the same depth as in T. In particular, $\delta(T') = \delta$ and then $T' = T_{n;l_2,\ldots,l_j}$. Moreover, $p_1(T') = p_1 + 15$. Therefore, using the expression for the variance of a tree of type $T_{n;l'_1,\ldots,l'_{j'}}$ given in Lemma 2, we have that

$$n^2 \cdot V(T) = n\Big(p_1 + 16 + \sum_{i=2}^{j} l_i^2\Big) - \Big(p_1 + 4 + \sum_{i=2}^{j} l_i\Big)^2$$

$$n^2 \cdot V(T') = n\Big(p_1 + 15 + \sum_{i=2}^{j} l_i^2\Big) - \Big(p_1 + 15 + \sum_{i=2}^{j} l_i\Big)^2$$

Then, $V(T') < V(T)$, against the assumption that T had the minimum value of V of $\mathcal{BT}_n^*$. So, when $p_0 > n/2$ we also conclude that if T has the minimum value of V of $\mathcal{BT}_n^*$, $l_1 > 4$. □

SN-7 Proof of Theorem 3

To simplify the language, in this section and in Section SN-13, for every sequence $\underline{l} = (l_1, \ldots, l_j) \in \mathbb{N}^j$ we shall set

$$A(\underline{l}) := \sum_{i=1}^{j} (2^{l_i} - l_i^2 - 1), \quad B(\underline{l}) := \sum_{i=1}^{j} (2^{l_i} - l_i - 1).$$

Our goal is to prove the following result:

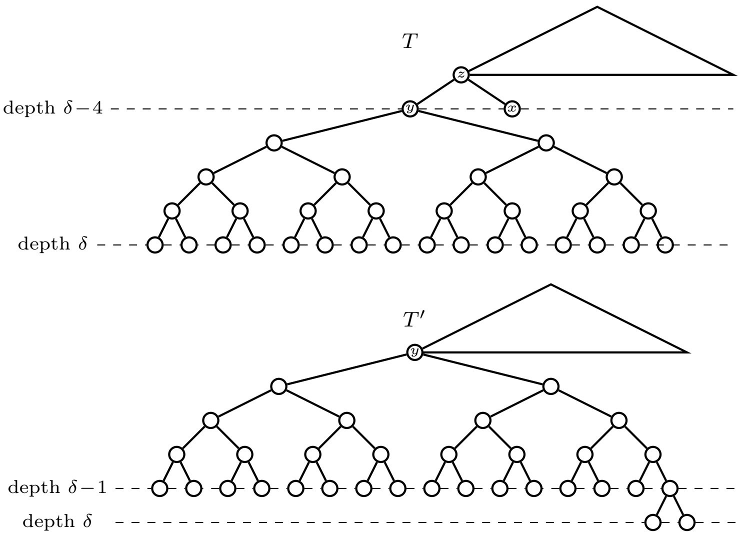

Figure 13 The trees T and T' appearing in the last part of the proof of Lemma 10.

Theorem 3 *As m grows to ∞, the fraction of values $n \in [2, 2^m]$ such that $V(B_n)$ is minimal on $\mathcal{BT}_n^*$ tends to 0.*

Proof Let $n = 2^m + k$ with $m = \lfloor \log_2(n) \rfloor$ and $0 \leqslant k < 2^m$, and take a non-empty sequence of indices $5 \leqslant l_1 < \cdots < l_j \leqslant m-1$ such that $k \leqslant 2^m - \sum_{i=1}^{j}(2^{l_i} - 1)$, so that

$$V(T_{n;l_1,\ldots,l_j}) = \frac{1}{n^2}\Big((2^m + k)\big(2^m - k - A(\underline{l})\big) - \big(2^m - k - B(\underline{l})\big)^2\Big).$$

Then

$$\begin{aligned} & n^2(V(B_n) - V(T_{n;l_1,\ldots,l_j})) \\ & \quad = 2k(2^m - k) - (2^m + k)\big(2^m - k - A(\underline{l})\big) + \big(2^m - k - B(\underline{l})\big)^2 \\ & \quad = k(A(\underline{l}) + 2B(\underline{l})) + 2^m(A(\underline{l}) - 2B(\underline{l})) + B(\underline{l})^2. \end{aligned}$$

Since $A(\underline{l}) + 2B(\underline{l}) > 0$ because each $l_i \geqslant 5$ (a fact that we shall use henceforth without any further notice), this implies that

$$V(B_n) > V(T_{n;l_1,\ldots,l_j}) \Longleftrightarrow k > \frac{2^m(2B(\underline{l}) - A(\underline{l})) - B(\underline{l})^2}{A(\underline{l}) + 2B(\underline{l})}.$$

So, we have the following fact:

Claim 1. If there exist $5 \leqslant l_1 < \cdots < l_j \leqslant m-1$, with $j \geqslant 1$, such that

$$\frac{2^m(2B(\underline{l}) - A(\underline{l})) - B(\underline{l})^2}{A(\underline{l}) + 2B(\underline{l})} < k \leqslant 2^m - \sum_{i=1}^{j}(2^{l_i} - 1),$$

then $V(B_{2^m+k})$ is not minimal on $\mathcal{BT}_{2^m+k}^*$.

Consider now the particular case of tree types $T_{n;l_1}$, i.e., with $j=1$. Set $l_1 = x \in \{5,\dots,m-1\}$, so that $A(\underline{l}) = 2^x - x^2 - 1$ and $B(\underline{l}) = 2^x - x - 1$. Then, Claim 1 implies that if $n = 2^m + k$ and if k belongs to

$$\bigcup_{x=5}^{m-1} \left(\frac{2^m(2^x + x^2 - 2x - 1) - (2^x - x - 1)^2}{3 \cdot 2^x - x^2 - 2x - 3}, 2^m - 2^x + 1 \right]$$

then $V(B_n)$ is not minimal on $\mathcal{BT}^*_n$. To simplify the notations, let

$$F_1(x) \coloneqq \frac{2^m(2^x + x^2 - 2x - 1) - (2^x - x - 1)^2}{3 \cdot 2^x - x^2 - 2x - 3}, \quad G_1(x) \coloneqq 2^m - 2^x + 1,$$

so that this union of intervals can be rewritten as

$$\bigcup_{x=5}^{m-1} \big(F_1(x), G_1(x)\big]. \tag{38}$$

We shall prove that there exists an $m_1 \in \mathbb{N}$ such that, for every $m \geqslant m_1$, this union of intervals is the interval $(F_1(m-1), G_1(5)]$. To do that, we use of the next two Claims.

Claim 2. For every $m \geqslant 7$ and for every $x \in \{5,\dots,m-2\}$, $F_1(x+1) < F_1(x)$ and $G_1(x+1) < G_1(x)$.

The decreasing monotonicity of G_1 is clear. As far as that of F_1 goes, we have that

$$\begin{aligned}
&F_1(x) > F_1(x+1)\\
&\quad\Longleftrightarrow \big(2^m(2^x + x^2 - 2x - 1) - (2^x - x - 1)^2\big)\\
&\qquad\qquad\qquad \cdot \big(3 \cdot 2^{x+1} - (x+1)^2 - 2(x+1) - 3\big)\\
&\qquad\quad > \big(2^m(2^{x+1} + (x+1)^2 - 2(x+1) - 1) - (2^{x+1} - (x+1) - 1)^2\big)\\
&\qquad\qquad\qquad \cdot \big(3 \cdot 2^x - x^2 - 2x - 3\big)\\
&\quad\Longleftrightarrow 2^x\big(6 \cdot 2^{2x} + 2^{m+2}x^2 - 3 \cdot 2^x x^2 - 3 \cdot 2^{m+2}x - 4 \cdot 2^x x - 18 \cdot 2^x\\
&\qquad\qquad\qquad + 2x^3 + 3x^2 + 8x + 18\big) + 2^{m+2}(x^2 + 3x) - 4x - 6 > 0
\end{aligned}$$

Now, if $5 \leqslant x \leqslant m-2$,

$$\begin{aligned}
&2^x\big(6 \cdot 2^{2x} + 2^{m+2}x^2 - 3 \cdot 2^x x^2 - 3 \cdot 2^{m+2}x - 4 \cdot 2^x x - 18 \cdot 2^x\\
&\qquad\qquad + 2x^3 + 3x^2 + 8x + 18\big) + 2^{m+2}(x^2 + 3x) - 4x - 6\\
&\quad \geqslant 2^{m+2}x^2 - 3 \cdot 2^x x^2 - 3 \cdot 2^{m+2}x - 4 \cdot 2^x x - 18 \cdot 2^x\\
&\text{(because } x \geqslant 5)\\
&\quad \geqslant 2^{m+2}x^2 - 3 \cdot 2^{m-2}x^2 - 3 \cdot 2^{m+2}x - 4 \cdot 2^{m-2}x - 18 \cdot 2^{m-2}\\
&\text{(because } x \leqslant m-2)\\
&\qquad = 2^{m-2}(13x^2 - 52x - 18) > 0
\end{aligned}$$

again because $x \geqslant 5$. This finishes the proof of Claim 2.

Claim 3. For every $m \geqslant 9$ and for every $x \in \{5, \dots, m-2\}$, $G_1(x+1) > F_1(x)$.

Indeed, we have that

$$\begin{aligned} &G_1(x+1) > F_1(x) \\ &\quad \Longleftrightarrow (2^m - 2^{x+1} + 1)(3 \cdot 2^x - x^2 - 2x - 3) \\ &\qquad\quad - (2^m(2^x + x^2 - 2x - 1) - (2^x - x - 1)^2) > 0 \\ &\quad \Longleftrightarrow 2^{m+1}(2^x - x^2 - 1) - 2^x(5 \cdot 2^x - 2x^2 - 2x - 7) - 2 > 0 \\ &\quad \Longleftrightarrow 2^{m-2}(3 \cdot 2^x - 8x^2 - 8) + 2^x(5 \cdot 2^{m-2} - 5 \cdot 2^x + 2x^2 + 2x + 7) - 2 > 0 \end{aligned}$$

Now, it turns out that if $m \geqslant 9$ and if $5 \leqslant x \leqslant m-2$, then this last inequality holds:

- If $8 \leqslant x \leqslant m-2$, because for this range of values of x and for every $m \geqslant 10$,

$$3 \cdot 2^x - 8x^2 - 8 > 0$$

and

$$2^x(5 \cdot 2^{m-2} - 5 \cdot 2^x + 2x^2 + 2x + 7) \geqslant 2^x(2x^2 + 2x + 7) \geqslant 38656.$$

- If $x \in \{5, 6, 7\}$, because

$$\begin{aligned} &2^{m-2}(3 \cdot 2^5 - 8 \cdot 5^2 - 8) + 2^5(5 \cdot 2^{m-2} - 5 \cdot 2^5 + 2 \cdot 5^2 + 2 \cdot 5 + 7) - 2 \\ &\qquad = 12 \cdot 2^m - 2978 \\ &2^{m-2}(3 \cdot 2^6 - 8 \cdot 6^2 - 8) + 2^6(5 \cdot 2^{m-2} - 5 \cdot 2^6 + 2 \cdot 6^2 + 2 \cdot 6 + 7) - 2 \\ &\qquad = 6(9 \cdot 2^m - 2443) \\ &2^{m-2}(3 \cdot 2^7 - 8 \cdot 7^2 - 8) + 2^7(5 \cdot 2^{m-2} - 5 \cdot 2^7 + 2 \cdot 7^2 + \cdot 7 + 7) - 2 \\ &\qquad = 78(2 \cdot 2^m - 855) \end{aligned}$$

  and hence they are all > 0 if $m \geqslant 9$.

This finishes the proof of Claim 3.

Claims 2 and 3 imply that there exists an $m_1 \in \mathbb{N}$ (in fact, they even imply that we can take $m_1 = 9$) such that if $m \geqslant m_1$, the intervals $(F_1(x), G_1(x)]$ satisfy that both their left-hand side and right-hand side ends decrease with x, and each interval $(F_1(x), G_1(x)]$, for $x = 6, \dots, m-1$, has non-empty overlap with the interval $(F_1(x-1), G_1(x-1)]$ on its right-hand side. Therefore, when $m \geqslant m_1$,

$$\bigcup_{x=5}^{m-1} (F_1(x), G_1(x)] = (F_1(m-1), G_1(5)].$$

The next claim summarizes what we have proved so far:

Claim 4. There exists an $m_1 \in \mathbb{N}$ such that, for every $m \geqslant m_1$, if $k \in \big(F_1(m-1), G_1(5)\big]$, then $V(B_{2^m+k})$ is not minimal on $\mathcal{BT}^*_{2^m+k}$.

Let us consider now another particular case of tree types $T_{n;l_1,\dots,l_j}$, namely those with $j = m-5$ and $\{l_1,\dots,l_{m-5}\} = \{5,\dots,x-1,x+1,\dots,m-1\}$, for some $x = 6,\dots,m-2$. In this case,

$$A(\underline{l}) = 2^m - 2^x + x^2 - \frac{2m^3-3m^2+7m-24}{6}$$
$$B(\underline{l}) = 2^m - 2^x + x - \frac{m^2+m+32}{2}$$
$$2^m - \Big(\sum_{j=5}^{m-1}(2^j-1) - (2^x-1)\Big) = 2^x + m + 26$$

and hence

$$2^m(2B(\underline{l}) - A(\underline{l})) - B(\underline{l})^2 = \frac{1}{12}\Big(2^{m+1}(6\cdot 2^x + 2m^3 - 3m^2 + 7m - 6x^2 - 24) - 3(2^{x+1} - 2x + m^2 + m + 32)^2\Big)$$
$$A(\underline{l}) + 2B(\underline{l}) = \frac{1}{6}\big(9\cdot 2^{m+1} - 9\cdot 2^{x+1} - 2m^3 - 3m^2 - 13m + 6x^2 + 12x - 168\big)$$

Thus, writing

$$F_2^{(num)}(x) = 2^{m+1}(6\cdot 2^x + 2m^3 - 3m^2 + 7m - 6x^2 - 24) - 3(2^{x+1} - 2x + m^2 + m + 32)^2$$
$$F_2^{(den)}(x) = 2\big(9\cdot 2^{m+1} - 9\cdot 2^{x+1} - 2m^3 - 3m^2 - 13m + 6x^2 + 12x - 168\big)$$
$$F_2(x) = F_2^{(num)}(x)/F_2^{(den)}(x)$$
$$G_2(x) = 2^x + m + 26$$

Claim 1 implies that if $n = 2^m + k$ and k belongs to

$$\bigcup_{x=6}^{m-2}\big(F_2(x), G_2(x)\big] \tag{39}$$

then $V(B_n)$ is not minimal on $\mathcal{BT}_n^*$. We shall prove now that there exists an $m_2 \in \mathbb{N}$ such that, for every $m \geqslant m_2$,

$$\bigcup_{x=5\lceil \log_2(m)\rceil}^{m-2}\big(F_2(x), G_2(x)\big] = \big(F_2(5\lceil \log_2(m)\rceil), G_2(m-2)\big].$$

This proof is based on the obvious fact that G_2 is increasing on x and the following two Claims.

Claim 5. There exists an $m_2' \in \mathbb{N}$ such that, for every $m \geqslant m_2'$ and for every $x \in \{6,\dots,m-3\}$, $F_2(x+1) > F_2(x)$.

Indeed, on the one hand notice that $F_2^{(den)}(x)$ can be written as

$$2\big(9\cdot 2^{m+1} - 2m^3 - 3m^2 - 13m - 168 - 6(3\cdot 2^x - x^2 - 2x)\big),$$

and this expression is decreasing for $x \in [6, m-2]$ because the function $x \mapsto 3 \cdot 2^x - x^2 - 2x$ is increasing on $[6, \infty)$. As far as $F_2^{(num)}(x)$ goes, its derivative is, up to a factor of 12 that does not affect its sign,

$$2^x \ln(2)(2^m - 2^{x+1} - m^2 - m + 2x - 32) + 2^{x+1} - 2^{m+1}x + m^2 + m - 2x + 32. \quad (40)$$

Now, when $x \leqslant m-2$

$$\begin{aligned} &2^x \ln(2)(2^m - 2^{x+1} - m^2 - m + 2x - 32) \\ &\qquad\qquad + 2^{x+1} - 2^{m+1}x + m^2 + m - 2x + 32 \\ &\quad \geqslant 2^x \ln(2)(2^m - 2^{m-1} - m^2 - m + 2x - 32) \\ &\qquad\qquad + 2^{x+1} - 2^{m+1}x + m^2 + m - 2x + 32 \\ &\quad = 2^x \ln(2)(2^{m-1} - m^2 - m + 2x - 32) \\ &\qquad\qquad + 2^{x+1} - 2^{m+1}x + m^2 + m - 2x + 32 \\ &\quad = 2^x \ln(2)(2^{m-3} - m^2 - m + 2x - 32) \\ &\qquad\qquad + 2^{m-3}(3 \cdot 2^x \ln(2) - 16x) + m^2 + m + 2^{x+1} - 2x + 32 \end{aligned}$$

In this last expression, if $x \geqslant 6$ then $3 \cdot 2^x \ln(2) - 16x > 0$ and $2^{x+1} - 2x > 0$, and if m is large enough, $2^{m-3} - m^2 - m - 32 > 0$. Therefore, if m is large enough, the derivative (40) is positive on the interval $[6, m-2]$ and therefore $F_2^{(num)}(x)$ is increasing on this interval.

So, on $[6, m-2]$, $F_2^{(num)}(x)$ is increasing and $F_2^{(den)}(x)$ is decreasing and thus $F_2(x)$ is increasing. This finishes the proof of Claim 5.

Claim 6. There exists an $m_2'' \in \mathbb{N}$ such that for every $m \geqslant m_2''$ and for every $3\log_2(m) \leqslant x \leqslant m-3$, $F_2(x+1) < G_2(x)$

Indeed, the inequality

$$F_2(x+1) = \frac{F_2^{(num)}(x+1)}{F_2^{(den)}(x+1)} < G_2(x)$$

is equivalent to

$$F_2^{(den)}(x+1) \cdot G_2(x) > F_2^{(num)}(x+1),$$

that is, to

$$\begin{aligned} 2\big(9(2^{m+1} - 2^{x+2}) - 2m^3 - 3m^2 - 13m + 6(x+1)^2 + 12(x+1) - 168\big)(2^x + m + 26) \\ - 2^{m+1}(6 \cdot 2^{x+1} + 2m^3 - 3m^2 + 7m - 6(x+1)^2 - 24) \\ + 3(2^{x+2} - 2(x+1) + m^2 + m + 32)^2 > 0 \end{aligned}$$

Let us develop the expression in left-hand side of this inequality

$$\begin{aligned}
&2\big(9(2^{m+1}-2^{x+2})-2m^3-3m^2-13m+6(x+1)^2+12(x+1)-168\big)(2^x+m+26)\\
&\qquad\qquad -2^{m+1}(6\cdot 2^{x+1}+2m^3-3m^2+7m-6(x+1)^2-24)\\
&\qquad\quad +3(2^{x+2}-2(x+1)+m^2+m+32)^2\\
&\quad = 2^{x+1}(3\cdot 2^{m+1}-3\cdot 2^{x+2}-2m^3+9m^2-37m+6x^2-726)\\
&\qquad +2^{m+1}(-2m^3+3m^2+11m+6x^2+12x+498)\\
&\qquad -m^4-104m^3-m^2\,(12x-1)+m(36x-796)\\
&\qquad +12mx^2+324x^2+888x-5100\\
&\quad \geqslant 2^{x+1}(3\cdot 2^{m+1}-3\cdot 2^{m-1}-2m^3+9m^2-37m+6x^2-726)\\
&\qquad +2^{m+1}(-2m^3+3m^2+11m+6x^2+12x+498)\\
&\qquad -m^4-104m^3-m^2\,(12x-1)+m(36x-796)\\
&\qquad +12mx^2+324x^2+888x-5100\\
&(\text{because } x\leqslant m-3)\\
&\quad = 2^{x+1}(9\cdot 2^{m-1}-2m^3+9m^2-37m+6x^2-726)\\
&\qquad +2^{m+1}(-2m^3+3m^2+11m+6x^2+12x+498)\\
&\qquad -m^4-104m^3-m^2(12x-1)+m(36x-796)\\
&\qquad +12mx^2+324x^2+888x-5100\\
&\quad = 2^{x+1}(5\cdot 2^{m-1}-2m^3+9m^2-37m+6x^2-726)\\
&\qquad +2^{m+1}(2^{x+1}-2m^3+3m^2+11m+6x^2+12x+498)\\
&\qquad -m^4-104m^3-m^2(12x-1)+m(36x-796)\\
&\qquad +12mx^2+324x^2+888x-5100
\end{aligned}\tag{41}$$

Now, on the one hand,

$$5\cdot 2^{m-1}-2m^3+9m^2-37m+6x^2-726\geqslant 5\cdot 2^{m-1}-2m^3+9m^2-37m-726$$

and if m is large enough the expression on the right-hand side of this inequality is positive. On the other hand, if $3\log_2(m)\leqslant x\leqslant m-3$, then

$$\begin{aligned}
&2^{m+1}(2^{x+1}-2m^3+3m^2+11m+6x^2+12x+498)\\
&\qquad -m^4-104m^3-m^2(12x-1)+m(36x-796)\\
&\qquad +12mx^2+324x^2+888x-5100\\
&\quad\geqslant 2^{m+1}(2m^3-2m^3+3m^2+11m+54\log_2(m)^2+36\log_2(m)+498)\\
&\qquad -m^4-104m^3-m^2(12(m-3)-1)+m(108\log_2(m)-796)\\
&\qquad +108m\log_2(m)^2+2664\log_2(m)-5100
\end{aligned}$$

and if m is large enough, this expression (which is dominated by $3\cdot 2^{m+1}m^2$) is also positive. Therefore, expression (41) is positive for large enough m, which proves Claim 6.

Claims 5 and 6 jointly imply that there exists an $m_2 \in \mathbb{N}$ such that if $m \geqslant m_2$ and $3\log_2(m) \leqslant x \leqslant m-2$, the intervals $(F_2(x), G_2(x)]$ satisfy that both their left-hand side and right-hand side ends increase with x, and each interval $(F_2(x), G_2(x)]$, for $x = \lceil 3\log_2(m)\rceil, \dots, m-3$, has non-empty overlap with the "next" interval $(F_2(x+1), G_2(x+1)]$. Therefore, when $m \geqslant m_2$,

$$\bigcup_{x=\lceil \log_2(m^3)\rceil}^{m-2} (F_2(x), G_2(x)] = (F_2(\lceil \log_2(m^3)\rceil), G_2(m-2)].$$

So, using Claim 1, we deduce the following claim:

Claim 7. There exists an $m_2 \in \mathbb{N}$ such that, for every $m \geqslant m_2$, if

$$k \in \big(F_2(\lceil \log_2(m^3)\rceil), G_2(m-2)\big],$$

then $V(B_{2^m+k})$ is not minimal on $\mathcal{BT}^*_{2^m+k}$.

Now, it turns out that if m is large enough, the intervals

$$\big(F_2(\lceil \log_2(m^3)\rceil), G_2(m-2)\big],\ \big(F_1(m-1), G_1(5)\big]$$

overlap: more specifically, for large enough m we have that

$$F_2(\lceil \log_2(m^3)\rceil) < F_1(m-1) < G_2(m-2) < G_1(5).$$

Indeed, on the one hand, the inequality $G_2(m-2) < G_1(5)$, that is, $2^{m-2}+m+26 < 2^m - 31$, holds if $m \geqslant 7$. On the other hand, the inequality

$$\begin{aligned}
&F_1(m-1) < G_2(m-2)\\
&\iff 4^{m-1} + 2^m(m-1)(m-2) - m^2 < (2^{m-2}+m+26)(3\cdot 2^{m-1} - m^2 - 2)\\
&\iff 2^{2m} - 10\cdot 2^m m^2 + 36\cdot 2^m m + 292\cdot 2^m - 8m^3 - 200m^2 - 16m - 416 > 0
\end{aligned}$$

holds if $m \geqslant 6$. Finally, the inequality $F_2(\lceil \log_2(m^3)\rceil) < F_1(m-1)$ holds for large enough m because $F_2(\lceil \log_2(m^3)\rceil)$ is in $O(m^3)$ and $F_1(m-1)$ is in $O(2^m)$.

Therefore, there exists an $m_3 \in \mathbb{N}$ such that if $m \geqslant m_3$,

$$\big(F_2(\lceil \log_2(m^3)\rceil), G_2(m-2)\big] \cup \big(F_1(m-1), G_1(5)\big] = \big(F_2(\lceil \log_2(m^3)\rceil), G_1(5)\big]$$

and setting $M = \max\{m_1, m_2, m_3\}$ we deduce from Claims 4 and 7 that, for every $m \geqslant M$, if

$$n \in \big(2^m + F_2(\lceil \log_2(m^3)\rceil), 2^m + G_1(5)\big]$$

then $V(B_n)$ is not minimal on $\mathcal{BT}^*_n$. Let us mention incidentally that $2^m + G_1(5) = 2^{m+1} - 31$ and in Section SN-13 we shall prove that this end can be replaced optimally by $2^{m+1} - 30$. This does not change what remains of our argument.

Since $F_2(\lceil \log_2(m^3) \rceil) \sim \frac{4}{9}m^3$, we deduce that, for every $m \geqslant M$, the cardinality of the set of numbers $n \in [2^m, 2^{m+1})$ such that $V(B_n)$ is minimal on $\mathcal{BT}^*_n$ is in $O(m^3)$. Then, for every $m \geqslant M$, the *fraction* of values $n \in [2, 2^{m+1})$ such that $V(B_n)$ is minimal on $\mathcal{BT}^*_n$ is bounded from above by

$$O\Big(\frac{2^{M+1} + \sum_{p=M+1}^{m} p^3}{2^{m+1}}\Big) = O\Big(\frac{m^4}{2^{m+1}}\Big)$$

which tends to 0 as $m \to \infty$. □

SN-8 Proof of Proposition 1

To begin with, notice that the index $S^{(2)}$ satisfies the following recurrence.

Lemma 11 *For every $T \in \mathcal{T}^*_n$, if $T = T_1 \star \cdots \star T_k$ with $k \geqslant 2$, then*

$$S^{(2)}(T) = \sum_{i=1}^{k} S^{(2)}(T_i) + 2\sum_{i=1}^{k} S(T_i) + n.$$

Proof Under the hypothesis in the statement,

$$\begin{aligned} S^{(2)}(T) &= \sum_{i=1}^{k} \sum_{x \in L(T_i)} \delta_T(x)^2 = \sum_{i=1}^{k} \sum_{x \in L(T_i)} (\delta_{T_i}(x) + 1)^2 \\ &= \sum_{i=1}^{k} \sum_{x \in L(T_i)} \delta_{T_i}(x)^2 + 2\sum_{i=1}^{k} \sum_{x \in L(T_i)} \delta_{T_i}(x) + \sum_{i=1}^{k} |L(T_i)| \\ &= \sum_{i=1}^{k} S^{(2)}(T_i) + 2\sum_{i=1}^{k} S(T_i) + n \end{aligned}$$

as we claimed. □

Now, our goal is to prove the following proposition.

Proposition 1 *For every $n \geqslant 1$, $E_Y(S^{(2)}_n) = 2n(2H_n^2 - 3H_n - 2H_n^{(2)} + 3)$.*

Proof When $n = 1$, both sides of the identity in the statement are equal to 0, and therefore we shall consider henceforth only the case $n \geqslant 2$. Recall from equation (11) that, if $T_k \in \mathcal{BT}(X_k)$, where $X_k \subsetneq [n]$ with $|X_k| = k$, and $T'_{n-k} \in \mathcal{BT}(X_k^c)$, where $X_k^c = [n] \setminus X_k$, then

$$P_{Y,n}(T_k \star T'_{n-k}) = \frac{2}{(n-1)\binom{n}{k}} P_{Y,k}(T_k) P_{Y,n-k}(T'_{n-k}).$$

Since every $T \in \mathcal{BT}_n$, with $n \geqslant 2$, is produced twice by choosing an integer $k = 1, \ldots, n-1$, a subset $X_k \subseteq [n]$ with $|X_k| = k$, a tree $T_k \in \mathcal{BT}(X_k)$, and a tree $T'_{n-k} \in \mathcal{BT}(X_k^c)$, and taking $T = T_k \star T'_{n-k}$, equation (11) and Lemma 11 allow us

to compute $E_Y(S_n^{(2)})$ from its very definition as follows:

$$\begin{aligned}
E_Y(S_n^{(2)}) &= \sum_{T\in\mathcal{BT}_n^*} S^{(2)}(T)\cdot P_{Y,n}(T)\\
&= \frac{1}{2}\sum_{k=1}^{n-1}\sum_{\substack{X_k\subseteq[n]\\|X_k|=k}}\sum_{T_k\in\mathcal{BT}(X_k)}\sum_{T'_{n-k}\in\mathcal{BT}(X_k^c)} S^{(2)}(T_k\star T'_{n-k})\cdot P_{Y,n}(T_k\star T'_{n-k})\\
&= \frac{1}{2}\sum_{k=1}^{n-1}\binom{n}{k}\sum_{T_k\in\mathcal{BT}_k^*}\sum_{T'_{n-k}\in\mathcal{BT}_{n-k}^*}(S^{(2)}(T_k)+S^{(2)}(T'_{n-k})\\
&\qquad +2S(T_k)+2S(T'_{n-k})+n)\cdot\frac{2}{(n-1)\binom{n}{k}}P_{Y,k}(T_k)P_{Y,n-k}(T'_{n-k})\\
&\text{(by the shape invariance of } P_Y\text{, Lemma 11, and identity (11))}\\
&= \frac{1}{n-1}\sum_{k=1}^{n-1}\Big(\sum_{T_k}\sum_{T'_{n-k}} S^{(2)}(T_k)P_{Y,k}(T_k)P_{Y,n-k}(T'_{n-k})\\
&\qquad +\sum_{T_k}\sum_{T'_{n-k}} S^{(2)}(T'_{n-k})P_{Y,k}(T_k)P_{Y,n-k}(T'_{n-k})\\
&\qquad +2\sum_{T_k}\sum_{T'_{n-k}} S(T_k)P_{Y,k}(T_k)P_{Y,n-k}(T'_{n-k})\\
&\qquad +2\sum_{T_k}\sum_{T'_{n-k}} S(T'_{n-k})P_{Y,k}(T_k)P_{Y,n-k}(T'_{n-k})\\
&\qquad +n\sum_{T_k}\sum_{T'_{n-k}} P_{Y,k}(T_k)P_{Y,n-k}(T'_{n-k})\Big)\\
&= \frac{1}{n-1}\sum_{k=1}^{n-1}\Big(\sum_{T_k} S^{(2)}(T_k)P_{Y,k}(T_k)+\sum_{T'_{n-k}} S^{(2)}(T'_{n-k})P_{Y,n-k}(T'_{n-k})\\
&\qquad +2\sum_{T_k} S(T_k)P_{Y,k}(T_k)+2\sum_{T'_{n-k}} S(T'_{n-k})P_{Y,n-k}(T'_{n-k})+n\Big)\\
&= \frac{1}{n-1}\sum_{k=1}^{n-1}(E_Y(S_k^{(2)})+E_Y(S_{n-k}^{(2)})+2E_Y(S_k)+2E_Y(S_{n-k})+n)\\
&= \frac{2}{n-1}\sum_{k=1}^{n-1}E_Y(S_k^{(2)})+\frac{4}{n-1}\sum_{k=1}^{n-1}E_Y(S_k)+n
\end{aligned}$$

In particular,

$$E_Y(S_{n-1}^{(2)}) = \frac{2}{n-2}\sum_{k=1}^{n-2}E_Y(S_k^{(2)})+\frac{4}{n-2}\sum_{k=1}^{n-2}E_Y(S_k)+n-1$$

and therefore

$$\begin{aligned}
E_Y(S_n^{(2)}) = &\frac{2}{n-1}E_Y(S_{n-1}^{(2)})+\frac{2}{n-1}\sum_{k=1}^{n-2}E_Y(S_k^{(2)})+\frac{4}{n-1}E_Y(S_{n-1})\\
&+\frac{4}{n-1}\sum_{k=1}^{n-2}E_Y(S_k)+n
\end{aligned}$$

$$
\begin{aligned}
&= \frac{2}{n-1}E_Y(S^{(2)}_{n-1}) + \frac{n-2}{n-1}\cdot\frac{2}{n-2}\sum_{k=1}^{n-2}E_Y(S^{(2)}_k) + \frac{4}{n-1}E_Y(S_{n-1}) \\
&\qquad + \frac{n-2}{n-1}\cdot\frac{4}{n-2}\sum_{k=1}^{n-2}E_Y(S_k) + \frac{n-2}{n-1}\cdot(n-1)+2 \\
&= \frac{2}{n-1}E_Y(S^{(2)}_{n-1}) + \frac{4}{n-1}E_Y(S_{n-1}) + \frac{n-2}{n-1}E_Y(S^{(2)}_{n-1}) + 2 \\
&= \frac{n}{n-1}E_Y(S^{(2)}_{n-1}) + \frac{4}{n-1}E_Y(S_{n-1}) + 2
\end{aligned}
$$

If we let $x_n = \frac{1}{n}E_Y(S^{(2)}_n)$, dividing this last equality by n we obtain

$$x_n = x_{n-1} + \frac{4}{n(n-1)}E_Y(S_{n-1}) + \frac{2}{n}$$

and using the formula for $E_Y(S_n)$ given in (13), this recurrence becomes

$$x_n = x_{n-1} + \frac{4}{n(n-1)}2(n-1)(H_{n-1}-1) + \frac{2}{n} = x_{n-1} + \frac{8}{n}H_{n-1} - \frac{6}{n}.$$

Then, since $x_1 = 0$ and

$$\sum_{k=1}^{n-1}\frac{H_k}{k+1} = \frac{1}{2}(H_n^2 - H_n^{(2)}),$$

(for a proof, see Lemma 1.(4) in the Supplementary Material of [1]), we have that

$$
\begin{aligned}
x_n &= \sum_{k=2}^{n}\frac{8H_{k-1}}{k} - \sum_{k=2}^{n}\frac{6}{k} = 8\sum_{k=1}^{n-1}\frac{H_k}{k+1} - 6(H_n-1) \\
&= 4(H_n^2 - H_n^{(2)}) - 6H_n + 6
\end{aligned}
$$

from where we finally deduce

$$E_Y(S^{(2)}_n) = nx_n = 2n(2H_n^2 - 2H_n^{(2)} - 3H_n + 3)$$

as we claimed. □

SN-9 Proof of Proposition 2

Proposition 2 *For every $n \geqslant 2$,*

$$E_U(S^{(2)}_n) = 2\sum_{k=1}^{n-1}C_{k,n-k}E_U(S^{(2)}_k) + 2n\cdot\frac{(2n-2)!!}{(2n-3)!!} - 3n.$$

Proof Arguing as in the beginning of the proof of Proposition 1, but using identity (12) instead of (11), we have:

$$
\begin{aligned}
E_U(S_n^{(2)}) &= \sum_{T\in\mathcal{BT}_n} S^{(2)}(T)\cdot P_{U,n}(T)\\
&= \frac{1}{2}\sum_{k=1}^{n-1}\sum_{\substack{X_k\subseteq[n]\\|X_k|=k}}\sum_{T_k\in\mathcal{BT}(X_k)}\sum_{T'_{n-k}\in\mathcal{BT}(X_k^c)} S^{(2)}(T_k\star T'_{n-k})\cdot P_{U,n}(T_k\star T'_{n-k})\\
&= \frac{1}{2}\sum_{k=1}^{n-1}\binom{n}{k}\sum_{T_k\in\mathcal{BT}_k}\sum_{T'_{n-k}\in\mathcal{BT}_{n-k}}(S^{(2)}(T_k)+S^{(2)}(T'_{n-k})\\
&\qquad +2S(T_k)+2S(T'_{n-k})+n)\cdot\frac{2C_{k,n-k}}{\binom{n}{k}}P_{U,k}(T_k)\cdot P_{U,n-k}(T'_{n-k})\\
&= \sum_{k=1}^{n-1}\sum_{T_k}\sum_{T'_{n-k}} C_{k,n-k}\big(S^{(2)}(T_k)+S^{(2)}(T'_{n-k})+2S(T_k)+2S(T'_{n-k})+n\big)\\
&\qquad \cdot P_{U,k}(T_k)\cdot P_{U,n-k}(T'_{n-k})\\
&= \sum_{k=1}^{n-1} C_{k,n-k}\Big(\sum_{T_k}\sum_{T'_{n-k}} S^{(2)}(T_k)P_{U,k}(T_k)P_{U,n-k}(T'_{n-k})\\
&\qquad +\sum_{T_k}\sum_{T'_{n-k}} S^{(2)}(T'_{n-k})P_{U,k}(T_k)P_{U,n-k}(T'_{n-k})\\
&\qquad +2\sum_{T_k}\sum_{T'_{n-k}} S(T_k)P_{U,k}(T_k)P_{U,n-k}(T'_{n-k})\\
&\qquad +2\sum_{T_k}\sum_{T'_{n-k}} S(T'_{n-k})P_{U,k}(T_k)P_{U,n-k}(T'_{n-k})\\
&\qquad +\sum_{T_k}\sum_{T'_{n-k}} nP_{U,k}(T_k)P_{U,n-k}(T'_{n-k})\Big)\\
&= \sum_{k=1}^{n-1} C_{k,n-k}\Big(\sum_{T_k} S^{(2)}(T_k)P_{U,k}(T_k)+\sum_{T'_{n-k}} S^{(2)}(T'_{n-k})P_{U,n-k}(T'_{n-k})\\
&\qquad +2\sum_{T_k} S(T_k)P_{U,k}(T_k)+2\sum_{T'_{n-k}} S(T'_{n-k})P_{U,n-k}(T'_{n-k})+n\Big)\\
&= \sum_{k=1}^{n-1} C_{k,n-k}\Big(E_U(S_k^{(2)})+E_U(S_{n-k}^{(2)})+2E_U(S_k)+2E_U(S_{n-k})+n\Big)\\
&= 2\sum_{k=1}^{n-1} C_{k,n-k}E_U(S_k^{(2)})+4\sum_{k=1}^{n-1} C_{k,n-k}E_U(S_k)+n\sum_{k=1}^{n-1} C_{k,n-k}
\end{aligned}
$$

(by the symmetry of $C_{k,n-k}$)

$$
\begin{aligned}
&= 2\sum_{k=1}^{n-1} C_{k,n-k}E_U(S_k^{(2)})+4\sum_{k=1}^{n-1} C_{k,n-k}k\cdot\frac{(2k-2)!!}{(2k-3)!!}\\
&\qquad -4\sum_{k=1}^{n-1} C_{k,n-k}k+n\sum_{k=1}^{n-1} C_{k,n-k}
\end{aligned}
$$

(by identity (14))

$$
= 2\sum_{k=1}^{n-1} C_{k,n-k}E_U(S_k^{(2)})+2n\Big(\frac{(2n-2)!!}{(2n-3)!!}-1\Big)-2n+n
$$

(by Lemmas 3 and 4)

$$
= 2\sum_{k=1}^{n-1} C_{k,n-k}E_U(S_k^{(2)})+2n\cdot\frac{(2n-2)!!}{(2n-3)!!}-3n
$$

as we claimed. □

SN-10 Proof of Proposition 3

We establish first a general result on expected values of bifurcating recursive shape indices in the sense of [4] under the uniform model.

Definition A *bifurcating recursive shape index* is a mapping I that associates to each bifurcating phylogenetic tree a real number $\mathbb{R}$ satisfying the following two conditions:

(a) It is invariant under tree isomorphisms and relabelings of leaves.

(b) There exists a symmetric mapping $f_I : \mathbb{N}\times\mathbb{N} \to \mathbb{R}$ such that, for every pair of bifurcating phylogenetic trees T, T' on disjoint sets of taxa X, X', respectively,

$$I(T \star T') = I(T) + I(T') + f(|X|, |X'|).$$

Lemma 12 *Let I and J be two bifurcating recursive shape indices, and let I_n and J_n be the random variables that choose a tree $T \in \mathcal{BT}_n$ and compute $I(T)$ and $J(T)$, respectively. Then, for every $n \geqslant 2$, the expected values of I_nJ_n and I_n^2 under the uniform model are*

$$\begin{aligned} E_U(I_nJ_n) = \sum_{k=1}^{n-1} C_{k,n-k}\Big(&2E_U(I_kJ_k) + 2E_U(I_k)E_U(J_{n-k}) \\ &+ 2f_I(k, n-k)E_U(J_k) + 2f_J(k, n-k)E_U(I_k) \\ &+ f_I(k, n-k)f_J(k, n-k)\Big) \end{aligned} \tag{42}$$

$$\begin{aligned} E_U(I_n^2) = \sum_{k=1}^{n-1} C_{k,n-k}\Big(&2E_U(I_k^2) + 2E_U(I_k)E_U(I_{n-k}) \\ &+ 4f_I(k, n-k)E_U(I_k) + f_I(k, n-k)^2\Big) \end{aligned} \tag{43}$$

Proof To prove (42), we develop $E_U(I_nJ_n)$ as we did with $E_(S_n^{(2)})$ in the proof of Proposition 2:

$$\begin{aligned} E_U(I_nJ_n) &= \sum_{T\in\mathcal{BT}_n} I(T)J(T)\cdot P_{U,n}(T) \\ &= \frac{1}{2}\sum_{k=1}^{n-1} \sum_{\substack{X_k\subsetneq[n]\\|X_k|=k}} \sum_{T_k\in\mathcal{BT}(X_k)} \sum_{T'_{n-k}\in\mathcal{BT}(X_k^c)} I(T_k\star T'_{n-k})J(T_k\star T'_{n-k})P_{U,n}(T_k\star T'_{n-k}) \\ &= \frac{1}{2}\sum_{k=1}^{n-1}\binom{n}{k} \sum_{T_k\in\mathcal{BT}_k} \sum_{T'_{n-k}\in\mathcal{BT}_{n-k}} \big(I(T_k) + I(T'_{n-k}) + f_I(k, n-k)\big) \\ &\quad\cdot \big(J(T_k) + J(T'_{n-k}) + f_J(k, n-k)\big)\frac{2C_{k,n-k}}{\binom{n}{k}} P_{U,k}(T_k)P_{U,n-k}(T'_{n-k}) \end{aligned}$$

$$
\begin{aligned}
&= \sum_{k=1}^{n-1} C_{k,n-k} \sum_{T_k \in \mathcal{BT}_k} \sum_{T'_{n-k} \in \mathcal{BT}_{n-k}} \big(I(T_k) + I(T'_{n-k}) + f_I(k, n-k) \big) \\
&\quad \cdot \big(J(T_k) + J(T'_{n-k}) + f_J(k, n-k) \big) P_{U,k}(T_k) P_{U,n-k}(T'_{n-k}) \\
&= \sum_{k=1}^{n-1} C_{k,n-k} \sum_{T_k} \sum_{T'_{n-k}} \Big(I(T_k)J(T_k) + I(T'_{n-k})J(T'_{n-k}) \\
&\quad + I(T_k)J(T'_{n-k}) + I(T'_{n-k})J(T_k) + f_J(k,n-k)I(T_k) \\
&\quad + f_J(k,n-k)I(T'_{n-k}) + f_I(k,n-k)J(T_k) + f_I(k,n-k)J(T'_{n-k}) \\
&\quad + f_I(k,n-k)f_J(k,n-k) \Big) P_{U,k}(T_k) P_{U,n-k}(T'_{n-k}) \\
&= \sum_{k=1}^{n-1} C_{k,n-k} \Bigg(\sum_{T_k} \sum_{T'_{n-k}} \Big(I(T_k)J(T_k) P_{U,k}(T_k) P_{U,n-k}(T'_{n-k}) \\
&\quad + I(T'_{n-k})J(T'_{n-k}) P_{U,k}(T_k) P_{U,n-k}(T'_{n-k}) \\
&\quad + I(T_k)J(T'_{n-k}) P_{U,k}(T_k) P_{U,n-k}(T'_{n-k}) \\
&\quad + I(T'_{n-k})J(T_k) P_{U,k}(T_k) P_{U,n-k}(T'_{n-k}) \\
&\quad + f_J(k,n-k) I(T_k) P_{U,k}(T_k) P_{U,n-k}(T'_{n-k}) \\
&\quad + f_J(k,n-k) I(T'_{n-k}) P_{U,k}(T_k) P_{U,n-k}(T'_{n-k}) \\
&\quad + f_I(k,n-k) J(T_k) P_{U,k}(T_k) P_{U,n-k}(T'_{n-k}) \\
&\quad + f_I(k,n-k) J(T'_{n-k}) P_{U,k}(T_k) P_{U,n-k}(T'_{n-k}) \\
&\quad + f_I(k,n-k) f_J(k,n-k) P_{U,k}(T_k) P_{U,n-k}(T'_{n-k}) \Big) \Bigg) \\
&= \sum_{k=1}^{n-1} C_{k,n-k} \Bigg(\sum_{T_k} I(T_k)J(T_k) P_{U,k}(T_k) \\
&\quad + \sum_{T'_{n-k}} I(T'_{n-k})J(T'_{n-k}) P_{U,n-k}(T'_{n-k}) \\
&\quad + \Big(\sum_{T_k} I(T_k) P_{U,k}(T_k) \Big) \Big(\sum_{T'_{n-k}} J(T'_{n-k}) P_{U,n-k}(T'_{n-k}) \Big) \\
&\quad + \Big(\sum_{T'_{n-k}} I(T'_{n-k}) P_{U,n-k}(T'_{n-k}) \Big) \Big(\sum_{T_k} J(T_k) P_{U,k}(T_k) \Big) \\
&\quad + f_J(k,n-k) \sum_{T_k} I(T_k) P_{U,k}(T_k) + f_J(k,n-k) \sum_{T'_{n-k}} I(T'_{n-k}) P_{U,n-k}(T'_{n-k}) \\
&\quad + f_I(k,n-k) \sum_{T_k} J(T_k) P_{U,k}(T_k) + f_I(k,n-k) \sum_{T'_{n-k}} J(T'_{n-k}) P_{U,n-k}(T'_{n-k}) \\
&\quad + f_I(k,n-k) f_J(k,n-k) \Bigg)
\end{aligned}
$$

$$
\begin{aligned}
&= \sum_{k=1}^{n-1} C_{k,n-k}\Big(E_U(I_kJ_k) + E_U(I_{n-k}J_{n-k}) + E_U(I_k)E_U(J_{n-k}) \\
&\quad + E_U(I_{n-k})E_U(J_k) + f_J(k,n-k)E_U(I_k) + f_J(k,n-k)E_U(I_{n-k}) \\
&\quad + f_I(k,n-k)E_U(J_k) + f_I(k,n-k)E_U(J_{n-k}) \\
&\quad + f_I(k,n-k)f_J(k,n-k)\Big) \\
&= \sum_{k=1}^{n-1} C_{k,n-k}\Big(2E_U(I_kJ_k) + 2E_U(I_k)E_U(J_{n-k}) \\
&\quad + 2f_J(k,n-k)E_U(I_k) + 2f_I(k,n-k)E_U(J_k) + f_I(k,n-k)f_J(k,n-k)\Big)
\end{aligned}
$$

using the symmetry of $C_{k,n-k}$, $f_I(k,n-k)$, and $f_J(k,n-k)$.

As for (43), it is obtained from (42) by taking $I = J$ in it. □

Now we can use equation (43) in the last lemma to derive the recurrence in Proposition 3.

Proposition 3 *For every* $n \geqslant 2$,

$$
E_U(S_n^2) = 2\sum_{k=1}^{n-1} C_{k,n-k}E_U(S_k^2) + \frac{5n^2}{2}\cdot\frac{(2n-2)!!!}{(2n-3)!!} - n(5n-2)
$$

Proof Applying equation (43) taking as I the Sackin index S, for which

$$
\begin{aligned}
&f_S(k,n-k) = n && [7] \\
&E_U(S_k) = k\Big(\frac{(2k-2)!!}{(2k-3)!!} - 1\Big) && [5, \text{Thm. } 22]
\end{aligned}
$$

we obtain

$$
\begin{aligned}
E_U(S_n^2) &= \sum_{k=1}^{n-1} C_{k,n-k}\,\Big(2E_U(S_k^2) + f_S(k,n-k)^2 + 4f_S(k,n-k)E_U(S_k) \\
&\qquad +2E_U(S_k)E_U(S_{n-k})\Big) \\
&= \sum_{k=1}^{n-1} C_{k,n-k}\,\Big(2E_U(S_k^2) + n^2 + 4nk\Big(\frac{(2k-2)!!}{(2k-3)!!} - 1\Big) \\
&\qquad +2k(n-k)\Big(\frac{(2k-2)!!}{(2k-3)!!} - 1\Big)\Big(\frac{(2(n-k)-2)!!}{(2(n-k)-3)!!} - 1\Big)\Big) \\
&= \sum_{k=1}^{n-1} C_{k,n-k}\,\Big(2E_U(S_k^2) + n^2 + 4nk\frac{(2k-2)!!}{(2k-3)!!} - 4nk \\
&\qquad +2k(n-k)\frac{(2k-2)!!}{(2k-3)!!}\cdot\frac{(2(n-k)-2)!!}{(2(n-k)-3)!!} + 2k(n-k) \\
&\qquad -2k(n-k)\frac{(2k-2)!!}{(2k-3)!!} - 2k(n-k)\cdot\frac{(2(n-k)-2)!!}{(2(n-k)-3)!!}\Big) \\
&= \sum_{k=1}^{n-1} C_{k,n-k}\,\Big(2E_U(S_k^2) + 4nk\frac{(2k-2)!!}{(2k-3)!!} - 2k^2 \\
&\qquad +2k(n-k)\frac{(2k-2)!!}{(2k-3)!!}\cdot\frac{(2(n-k)-2)!!}{(2(n-k)-3)!!} - 4k(n-k)\frac{(2k-2)!!}{(2k-3)!!}\Big) \\
&= (*)
\end{aligned}
$$

because, by the symmetry of $C_{k,n-k}$,

$$\sum_{k=1}^{n-1} C_{k,n-k}k(n-k)\frac{(2k-2)!!}{(2k-3)!!} = \sum_{k=1}^{n-1} C_{k,n-k}k(n-k)\cdot\frac{(2(n-k)-2)!!}{(2(n-k)-3)!!}$$

and

$$\begin{aligned}\sum_{k=1}^{n-1} C_{k,n-k}\big(n^2-4nk+2k(n-k)\big) &= \sum_{k=1}^{n-1} C_{k,n-k}\big((n-k)^2-3k^2\big)\\ &= -2\sum_{k=1}^{n-1} C_{k,n-k}k^2.\end{aligned}$$

Simplifying one step further the sum $(*)$, we finally obtain

$$\begin{aligned}E_U(S_n^2) = 2\sum_{k=1}^{n-1} C_{k,n-k}E_U(S_k^2) - 2\sum_{k=1}^{n-1} C_{k,n-k}k^2 + 4\sum_{k=1}^{n-1} C_{k,n-k}k^2\frac{(2k-2)!!}{(2k-3)!!}\\ + 2\sum_{k=1}^{n-1} C_{k,n-k}k(n-k)\frac{(2k-2)!!}{(2k-3)!!}\cdot\frac{(2(n-k)-2)!!}{(2(n-k)-3)!!} \qquad (44)\end{aligned}$$

The values of the first two sums appearing in the independent term in this recurrence can be computed using Lemmas 3 and 4:

$$\begin{aligned}\sum_{k=1}^{n-1} C_{k,n-k}k^2 &= 2\sum_{k=1}^{n-1} C_{k,n-k}\binom{k}{2} + \sum_{k=1}^{n-1} C_{k,n-k}\binom{k}{1}\\ &= \binom{n}{2}\Big(1-\frac{1}{2(n-1)}\cdot\frac{(2n-2)!!}{(2n-3)!!}\Big) + \frac{n}{2} = \frac{n^2}{2} - \frac{n}{4}\cdot\frac{(2n-2)!!}{(2n-3)!!}\end{aligned}$$

$$\begin{aligned}\sum_{k=1}^{n-1} C_{k,n-k}k^2\frac{(2k-2)!!}{(2k-3)!!}\\ &= 2\sum_{k=1}^{n-1} C_{k,n-k}\binom{k}{2}\frac{(2k-2)!!}{(2k-3)!!} + \sum_{k=1}^{n-1} C_{k,n-k}\binom{k}{1}\frac{(2k-2)!!}{(2k-3)!!}\\ &= \binom{n}{2}\Big(\frac{(2n-2)!!}{(2n-3)!!}-2\Big) + \frac{n}{2}\Big(\frac{(2n-2)!!}{(2n-3)!!}-1\Big)\\ &= \frac{n^2}{2}\cdot\frac{(2n-2)!!}{(2n-3)!!} - \frac{n(2n-1)}{2}\end{aligned}$$

As for the third sum, its value is

$$\begin{aligned}\sum_{k=1}^{n-1} C_{k,n-k}k(n-k)\frac{(2k-2)!!(2(n-k)-2)!!}{(2k-3)!!(2(n-k)-3)!!}\\ &= \sum_{k=1}^{n-1}\frac{n!(2k-3)!!(2(n-k)-3)!!k(n-k)(2k-2)!!(2(n-k)-2)!!}{2\cdot(2n-3)!!k!(n-k)!(2k-3)!!(2(n-k)-3)!!}\\ &= \frac{n!}{2\cdot(2n-3)!!}\sum_{k=1}^{n-1}\frac{k(n-k)2^{k-1}(k-1)!2^{n-k-1}(n-k-1)!}{k!(n-k)!}\\ &= \frac{n!}{2\cdot(2n-3)!!}\sum_{k=1}^{n-1}2^{n-2} = \frac{n!(n-1)2^{n-3}}{(2n-3)!!} = \frac{n(n-1)}{4}\cdot\frac{(2n-2)!!}{(2n-3)!!}\end{aligned}$$

So, the independent term of the expression for $E_U(S_n^2)$ given by equation (44) is

$$
\begin{aligned}
4\sum_{k=1}^{n-1} C_{k,n-k}k^2\frac{(2k-2)!!}{(2k-3)!!} - 2\sum_{k=1}^{n-1} C_{k,n-k}k^2 + \\
+2\sum_{k=1}^{n-1} C_{k,n-k}k(n-k)\frac{(2k-2)!!}{(2k-3)!!}\cdot\frac{(2(n-k)-2)!!}{(2(n-k)-3)!!} \\
= 4\Big(\frac{n^2}{2}\cdot\frac{(2n-2)!!}{(2n-3)!!} - \frac{n(2n-1)}{2}\Big) - 2\Big(\frac{n^2}{2} - \frac{n}{4}\cdot\frac{(2n-2)!!}{(2n-3)!!}\Big) \\
+\frac{n(n-1)}{2}\cdot\frac{(2n-2)!!}{(2n-3)!!} \\
= \frac{5n^2}{2}\cdot\frac{(2n-2)!!}{(2n-3)!!} - n(5n-2).
\end{aligned}
$$

This completes the proof of the identity in the statement. □

SN-11 Proof of Theorem 6.(b)

In this section we prove the following result.

Theorem *Let Φ_n be the random variable that takes a tree $T \in \mathcal{BT}_n$ and computes its total cophenetic index $\Phi(T)$. Then, for every $n \geqslant 2$, the variance of Φ_n under the uniform model is*

$$
\begin{aligned}
\sigma_U^2(\Phi_n) = \binom{n}{2}\frac{(2n-1)(7n^2-3n-2)}{30} - \binom{n}{2}\frac{5n^2-n-2}{32}\cdot\frac{(2n-2)!!}{(2n-3)!!} \\
-\frac{1}{4}\binom{n}{2}^2\Big(\frac{(2n-2)!!}{(2n-3)!!}\Big)^2
\end{aligned}
$$

Proof If we apply identity (43) in Lemma 12 taking as I the total cophenetic index Φ, for which

$$
\begin{aligned}
f_\Phi(k,n-k) &= \binom{k}{2} + \binom{n-k}{2} && \text{[5, Lem. 2]} \\
E_U(\Phi_k) &= \frac{1}{2}\binom{k}{2}\Big(\frac{(2k-2)!!}{(2k-3)!!} - 2\Big) && \text{[5, Thm. 23]}
\end{aligned}
$$

we obtain the following recurrence for $E_U(\Phi_n^2)$:

$$
\begin{aligned}
E_U(\Phi_n^2) = \sum_{k=1}^{n-1} C_{k,n-k}\Bigg(2E_U(\Phi_k^2) + \Big(\binom{k}{2} + \binom{n-k}{2}\Big)^2 \\
+ 2\Big(\binom{k}{2} + \binom{n-k}{2}\Big)\binom{k}{2}\Big(\frac{(2k-2)!!}{(2k-3)!!} - 2\Big) \\
+ \frac{1}{2}\binom{k}{2}\binom{n-k}{2}\Big(\frac{(2k-2)!!}{(2k-3)!!} - 2\Big)\Big(\frac{(2(n-k)-2)!!}{(2(n-k)-3)!!} - 2\Big)\Bigg) \qquad (45)
\end{aligned}
$$

Let us simplify this recurrence. To begin with, we have that

$$
\begin{aligned}
&\Big(\binom{k}{2}+\binom{n-k}{2}\Big)^2+2\Big(\binom{k}{2}+\binom{n-k}{2}\Big)\binom{k}{2}\Big(\frac{(2k-2)!!}{(2k-3)!!}-2\Big)\\
&\qquad+\frac{1}{2}\binom{k}{2}\binom{n-k}{2}\Big(\frac{(2k-2)!!}{(2k-3)!!}-2\Big)\Big(\frac{(2(n-k)-2)!!}{(2(n-k)-3)!!}-2\Big)\\
&\quad=\binom{k}{2}^2+\binom{n-k}{2}^2+2\binom{k}{2}\binom{n-k}{2}+2\binom{k}{2}^2\frac{(2k-2)!!}{(2k-3)!!}\\
&\qquad+2\binom{k}{2}\binom{n-k}{2}\frac{(2k-2)!!}{(2k-3)!!}-4\binom{k}{2}^2-4\binom{k}{2}\binom{n-k}{2}\\
&\qquad+\frac{1}{2}\binom{k}{2}\binom{n-k}{2}\frac{(2k-2)!!(2(n-k)-2)!!}{(2k-3)!!(2(n-k)-3)!!}+2\binom{k}{2}\binom{n-k}{2}\\
&\qquad-\binom{k}{2}\binom{n-k}{2}\frac{(2k-2)!!}{(2k-3)!!}-\binom{k}{2}\binom{n-k}{2}\frac{(2(n-k)-2)!!}{(2(n-k)-3)!!}\\
&\quad=\binom{n-k}{2}^2-3\binom{k}{2}^2+2\binom{k}{2}^2\frac{(2k-2)!!}{(2k-3)!!}\\
&\qquad+\binom{k}{2}\binom{n-k}{2}\frac{(2k-2)!!}{(2k-3)!!}-\binom{k}{2}\binom{n-k}{2}\frac{(2(n-k)-2)!!}{(2(n-k)-3)!!}\\
&\qquad+\frac{1}{2}\binom{k}{2}\binom{n-k}{2}\frac{(2k-2)!!(2(n-k)-2)!!}{(2k-3)!!(2(n-k)-3)!!}
\end{aligned}
$$

and then, using that $C_{k,n-k}=C_{n-k,k}$,

$$
\begin{aligned}
&\sum_{k=1}^{n-1}C_{k,n-k}\Bigg(\Big(\binom{k}{2}+\binom{n-k}{2}\Big)^2+2\Big(\binom{k}{2}+\binom{n-k}{2}\Big)\binom{k}{2}\Big(\frac{(2k-2)!!}{(2k-3)!!}-2\Big)\\
&\qquad+\frac{1}{2}\binom{k}{2}\binom{n-k}{2}\Big(\frac{(2k-2)!!}{(2k-3)!!}-2\Big)\Big(\frac{(2(n-k)-2)!!}{(2(n-k)-3)!!}-2\Big)\Bigg)\\
&\quad=\sum_{k=1}^{n-1}C_{k,n-k}\Bigg(\binom{n-k}{2}^2-3\binom{k}{2}^2+2\binom{k}{2}^2\frac{(2k-2)!!}{(2k-3)!!}\\
&\qquad+\binom{k}{2}\binom{n-k}{2}\frac{(2k-2)!!}{(2k-3)!!}-\binom{k}{2}\binom{n-k}{2}\frac{(2(n-k)-2)!!}{(2(n-k)-3)!!}\\
&\qquad+\frac{1}{2}\binom{k}{2}\binom{n-k}{2}\frac{(2k-2)!!(2(n-k)-2)!!}{(2k-3)!!(2(n-k)-3)!!}\Bigg)\\
&\quad=\sum_{k=1}^{n-1}C_{k,n-k}\Bigg(-2\binom{k}{2}^2+2\binom{k}{2}^2\frac{(2k-2)!!}{(2k-3)!!}\\
&\qquad+\frac{1}{2}\binom{k}{2}\binom{n-k}{2}\frac{(2k-2)!!(2(n-k)-2)!!}{(2k-3)!!(2(n-k)-3)!!}\Bigg)
\end{aligned}
$$

so that (45) becomes

$$
\begin{aligned}
E_U(\Phi_n^2)&=2\sum_{k=1}^{n-1}C_{k,n-k}E_U(\Phi_k^2)+2\sum_{k=1}^{n-1}C_{k,n-k}\binom{k}{2}^2\frac{(2k-2)!!}{(2k-3)!!}\\
&\quad-2\sum_{k=1}^{n-1}C_{k,n-k}\binom{k}{2}^2+\frac{1}{2}\sum_{k=1}^{n-1}C_{k,n-k}\binom{k}{2}\binom{n-k}{2}\frac{(2k-2)!!(2(n-k)-2)!!}{(2k-3)!!(2(n-k)-3)!!}.
\end{aligned}
\tag{46}
$$

Now, using Lemmas 3 and 4 we have that

$$\sum_{k=1}^{n-1} C_{k,n-k}\binom{k}{2}^2 = \sum_{k=1}^{n-1} C_{k,n-k}\Big(6\binom{k}{4}+6\binom{k}{3}+\binom{k}{2}\Big)$$
$$= 3\binom{n}{4}\Big(1-\frac{15}{16(n-1)}\cdot\frac{(2n-2)!!}{(2n-3)!!}\Big)+3\binom{n}{3}\Big(1-\frac{3}{4(n-1)}\cdot\frac{(2n-2)!!}{(2n-3)!!}\Big)$$
$$+\frac{1}{2}\binom{n}{2}\Big(1-\frac{1}{2(n-1)}\cdot\frac{(2n-2)!!}{(2n-3)!!}\Big)$$
$$= \frac{1}{2}\binom{n}{2}^2-\frac{n(15n^2-27n+10)}{2^7}\cdot\frac{(2n-2)!!}{(2n-3)!!}$$

$$\sum_{k=1}^{n-1} C_{k,n-k}\binom{k}{2}^2\frac{(2k-2)!!}{(2k-3)!!} = \sum_{k=1}^{n-1} C_{k,n-k}\Big(6\binom{k}{4}+6\binom{k}{3}+\binom{k}{2}\Big)\frac{(2k-2)!!}{(2k-3)!!}$$
$$= 3\binom{n}{4}\Big(\frac{(2n-2)!!}{(2n-3)!!}-\frac{16}{5}\Big)+3\binom{n}{3}\Big(\frac{(2n-2)!!}{(2n-3)!!}-\frac{8}{3}\Big)$$
$$+\frac{1}{2}\binom{n}{2}\Big(\frac{(2n-2)!!}{(2n-3)!!}-2\Big)$$
$$= \frac{1}{2}\binom{n}{2}^2\frac{(2n-2)!!}{(2n-3)!!}-\binom{n}{2}\frac{12n^2-20n+7}{15}$$

As for the remaining sum in the right hand side sum of (46),

$$\sum_{k=1}^{n-1} C_{k,n-k}\binom{k}{2}\binom{n-k}{2}\frac{(2k-2)!!(2(n-k)-2)!!}{(2k-3)!!(2(n-k)-3)!!}$$
$$= \sum_{k=2}^{n-2} C_{k,n-k}\binom{k}{2}\binom{n-k}{2}\frac{(2k-2)!!(2(n-k)-2)!!}{(2k-3)!!(2(n-k)-3)!!}$$
$$= \sum_{k=2}^{n-2}\frac{n!(2k-3)!!(2(n-k)-3)!!k!(n-k)!2^{k-1}(k-1)!2^{n-k-1}(n-k-1)!}{2(2n-3)!!k!(n-k)!2^2(k-2)!(n-k-2)!(2k-3)!!(2(n-k)-3)!!}$$
$$= \frac{n!2^{n-5}}{(2n-3)!!}\sum_{k=2}^{n-2}(k-1)(n-k-1)$$
$$= \frac{n!2^{n-5}}{(2n-3)!!}\Big((n-1)\sum_{k=2}^{n-2}(k-1)-\sum_{k=2}^{n-2}(k-1)k\Big)$$
$$= \frac{n!2^{n-5}}{(2n-3)!!}\Big((n-1)\sum_{k=1}^{n-3}k-2\sum_{k=2}^{n-2}\binom{k}{2}\Big)$$
$$= \frac{n!2^{n-5}}{(2n-3)!!}\Big((n-1)\binom{n-2}{2}-2\binom{n-1}{3}\Big)=\frac{1}{4}\binom{n}{4}\frac{(2n-2)!!}{(2n-3)!!}$$

Therefore, returning back to (46), its independent term turns out to be

$$2\sum_{k=1}^{n-1} C_{k,n-k}\binom{k}{2}^2\frac{(2k-2)!!}{(2k-3)!!}-2\sum_{k=1}^{n-1} C_{k,n-k}\binom{k}{2}^2$$
$$+\frac{1}{2}\sum_{k=1}^{n-1} C_{k,n-k}\binom{k}{2}\binom{n-k}{2}\frac{(2k-2)!!(2(n-k)-2)!!}{(2k-3)!!(2(n-k)-3)!!}$$

$$
\begin{aligned}
&= 2\left(\frac{1}{2}\binom{n}{2}^2\frac{(2n-2)!!}{(2n-3)!!}-\binom{n}{2}\frac{12n^2-20n+7}{15}\right)\\
&\qquad -2\left(\frac{1}{2}\binom{n}{2}^2-\frac{n(15n^2-27n+10)}{2^7}\cdot\frac{(2n-2)!!}{(2n-3)!!}\right)+\frac{1}{8}\binom{n}{4}\frac{(2n-2)!!}{(2n-3)!!}\\
&=\frac{n(49n^3-57n^2-22n+24)}{192}\cdot\frac{(2n-2)!!}{(2n-3)!!}-\frac{n(n-1)(63n^2-95n+28)}{60}
\end{aligned}
$$

So, the sequence $E_U(\Phi_n^2)$ is the solution of the recurrence

$$
\begin{aligned}
X_n &= 2\sum_{k=1}^{n-1} C_{k,n-k}X_k - \frac{63n^4-158n^3+123n^2-28n}{60}\\
&\qquad + \frac{49n^4-57n^3-22n^2+24n}{192}\cdot\frac{(2n-2)!!}{(2n-3)!!}\\
&= 2\sum_{k=1}^{n-1} C_{k,n-k}X_k - \frac{126}{5}\binom{n}{4}-22\binom{n}{3}-3\binom{n}{2}\\
&\qquad + \Big(\frac{49}{8}\binom{n}{4}+\frac{237}{32}\binom{n}{3}+\frac{25}{16}\binom{n}{2}-\frac{1}{32}n\Big)\frac{(2n-2)!!}{(2n-3)!!}
\end{aligned}
$$

with initial condition $X_1 = E_U(\Phi_1^2) = 0$. By Proposition 6, this solution is

$$
\begin{aligned}
E_U(\Phi_n^2) &= 28\binom{n}{5}+\frac{256}{5}\binom{n}{4}+26\binom{n}{3}+3\binom{n}{2}\\
&\qquad -\Big(\frac{63}{8}\binom{n}{4}+\frac{33}{4}\binom{n}{3}+\frac{3}{2}\binom{n}{2}\Big)\cdot\frac{(2n-2)!!}{(2n-3)!!}\\
&= \binom{n}{2}\Big(\frac{7n^3+n^2-8n+1}{15}-\frac{21n^2-17n-2}{32}\cdot\frac{(2n-2)!!}{(2n-3)!!}\Big)
\end{aligned}
$$

Finally,

$$
\begin{aligned}
\sigma_U(\Phi_n)^2 &= E_U(\Phi_n^2)-E_U(\Phi_n)^2\\
&= \binom{n}{2}\Big(\frac{7n^3+n^2-8n+1}{15}-\frac{21n^2-17n-2}{32}\cdot\frac{(2n-2)!!}{(2n-3)!!}\Big)\\
&\qquad -\frac{1}{4}\binom{n}{2}^2\Big(\frac{(2n-2)!!}{(2n-3)!!}-2\Big)^2\\
&= \binom{n}{2}\frac{(2n-1)(7n^2-3n-2)}{30}-\binom{n}{2}\frac{5n^2-n-2}{32}\cdot\frac{(2n-2)!!}{(2n-3)!!}\\
&\qquad -\frac{1}{4}\binom{n}{2}^2\Big(\frac{(2n-2)!!}{(2n-3)!!}\Big)^2.
\end{aligned}
$$

This completes the proof of Theorem 6.(b). □

SN-12 Proof of Theorem 6.(c)

In this section we prove the following result.

Theorem *Let S_n and Φ_n be, respectively, the random variable that take a tree $T \in \mathcal{BT}_n$ and compute its Sackin index $S(T)$ and its total cophenetic index $\Phi(T)$.*

Then, for every $n \geqslant 2$, the covariance of S_n and Φ_n under the uniform model is

$$
\begin{aligned}
Cov_U(\Phi_n, S_n) = \binom{n}{2}\frac{26n^2-5n-4}{15} - \frac{3n+2}{8}\binom{n}{2}\frac{(2n-2)!!}{(2n-3)!!} \\
-\frac{n}{2}\binom{n}{2}\Big(\frac{(2n-2)!!}{(2n-3)!!}\Big)^2
\end{aligned}
$$

Proof If we apply identity (42) in Lemma 12 taking as I and J the total cophenetic index Φ and the Sackin index S, for which we have that

$$
\begin{aligned}
f_\Phi(k,n-k) &= \binom{k}{2} + \binom{n-k}{2}, & E_U(\Phi_k) &= \frac{1}{2}\binom{k}{2}\Big(\frac{(2k-2)!!}{(2k-3)!!}-2\Big) \\
f_S(k,n-k) &= n, & E_U(S_k) &= k\Big(\frac{(2k-2)!!}{(2k-3)!!}-1\Big),
\end{aligned}
$$

we obtain

$$
\begin{aligned}
E_U(\Phi_n S_n) &= \sum_{k=1}^{n-1} C_{k,n-k}\Bigg(2E_U(\Phi_k S_k) + n\binom{k}{2}\Big(\frac{(2k-2)!!}{(2k-3)!!}-2\Big) \\
&\quad + \binom{k}{2}\Big(\frac{(2k-2)!!}{(2k-3)!!}-2\Big)(n-k)\Big(\frac{(2(n-k)-2)!!}{(2(n-k)-3)!!}-1\Big) \\
&\quad + 2\Big(\binom{k}{2}+\binom{n-k}{2}\Big)k\Big(\frac{(2k-2)!!}{(2k-3)!!}-1\Big) + n\Big(\binom{k}{2}+\binom{n-k}{2}\Big)\Bigg) \\
&= \sum_{k=1}^{n-1} C_{k,n-k}\Bigg(2E_U(\Phi_k S_k) + n\binom{k}{2} + n\binom{n-k}{2} - 4k\binom{k}{2} \\
&\quad - 2k\binom{n-k}{2} + (n-k)\binom{k}{2}\frac{(2k-2)!!(2(n-k)-2)!!}{(2k-3)!!(2(n-k)-3)!!} \\
&\quad - 2(n-k)\binom{k}{2}\frac{(2(n-k)-2)!!}{(2(n-k)-3)!!} + 3k\binom{k}{2}\frac{(2k-2)!!}{(2k-3)!!} \\
&\quad + 2k\binom{n-k}{2}\frac{(2k-2)!!}{(2k-3)!!}\Bigg) \\
&= (**)
\end{aligned}
$$

Now, using the symmetry of $C_{k,n-k}$ we have that

$$
\begin{aligned}
&\sum_{k=1}^{n-1} C_{k,n-k}\Big(n\binom{k}{2} + n\binom{n-k}{2} - 4k\binom{k}{2} - 2k\binom{n-k}{2}\Big) \\
&\qquad = \sum_{k=1}^{n-1} C_{k,n-k}\Big(n\binom{k}{2} + n\binom{k}{2} - 4k\binom{k}{2} - 2(n-k)\binom{k}{2}\Big) \\
&\qquad = -2\sum_{k=1}^{n-1} C_{k,n-k}\binom{k}{2}k
\end{aligned}
$$

and

$$
\sum_{k=1}^{n-1} C_{k,n-k}(n-k)\binom{k}{2}\frac{(2(n-k)-2)!!}{(2(n-k)-3)!!} = \sum_{k=1}^{n-1} C_{k,n-k}k\binom{n-k}{2}\frac{(2k-2)!!}{(2k-3)!!}
$$

and therefore we can simplify $(**)$ one step further, and we obtain:

$$\begin{aligned} E_U(\Phi_n S_n) &= (**) \\ &= 2\sum_{k=1}^{n-1} C_{k,n-k} E_U(\Phi_k S_k) + \sum_{k=1}^{n-1} C_{k,n-k}\Bigg(3k\binom{k}{2}\frac{(2k-2)!!}{(2k-3)!!} \\ &\qquad - 2k\binom{k}{2} + (n-k)\binom{k}{2}\frac{(2k-2)!!(2(n-k)-2)!!}{(2k-3)!!(2(n-k)-3)!!}\Bigg) \end{aligned} \tag{47}$$

We can compute now the three sums that form the independent term in this recurrence. To begin with, by Lemmas 3 and 4,

$$\begin{aligned} &\sum_{k=1}^{n-1} C_{k,n-k} k\binom{k}{2} = 3\sum_{k=1}^{n-1} C_{k,n-k}\binom{k}{3} + 2\sum_{k=1}^{n-1} C_{k,n-k}\binom{k}{2} \\ &\quad = \frac{3}{2}\binom{n}{3}\Big(1 - \frac{3}{4(n-1)}\cdot\frac{(2n-2)!!}{(2n-3)!!}\Big) + \binom{n}{2}\Big(1 - \frac{1}{2(n-1)}\cdot\frac{(2n-2)!!}{(2n-3)!!}\Big) \\ &\quad = \frac{n}{2}\binom{n}{2} - \frac{n(3n-2)}{16}\cdot\frac{(2n-2)!!}{(2n-3)!!} \\ &\sum_{k=1}^{n-1} C_{k,n-k} k\binom{k}{2}\frac{(2k-2)!!}{(2k-3)!!} \\ &\quad = 3\sum_{k=1}^{n-1} C_{k,n-k}\binom{k}{3}\frac{(2k-2)!!}{(2k-3)!!} + 2\sum_{k=1}^{n-1} C_{k,n-k}\binom{k}{2}\frac{(2k-2)!!}{(2k-3)!!} \\ &\quad = \frac{3}{2}\binom{n}{3}\Big(\frac{(2n-2)!!}{(2n-3)!!} - \frac{8}{3}\Big) + \binom{n}{2}\Big(\frac{(2n-2)!!}{(2n-3)!!} - 2\Big) \\ &\quad = \frac{n}{2}\binom{n}{2}\frac{(2n-2)!!}{(2n-3)!!} - \frac{2(2n-1)}{3}\binom{n}{2} \end{aligned}$$

As for the remaining sum,

$$\begin{aligned} &\sum_{k=1}^{n-1} C_{k,n-k}(n-k)\binom{k}{2}\frac{(2k-2)!!(2(n-k)-2)!!}{(2k-3)!!(2(n-k)-3)!!} \\ &\quad = \sum_{k=1}^{n-1}\frac{n!(2k-3)!!(2n-2k-3)!!(n-k)k(k-1)(2k-2)!!(2n-2k-2)!!}{4\cdot(2n-3)!!k!(n-k)!(2k-3)!!(2(n-k)-3)!!} \\ &\quad = \frac{n!}{4\cdot(2n-3)!!}\sum_{k=1}^{n-1}\frac{2^{k-1}(k-1)!2^{n-k-1}(n-k-1)!}{(k-2)!(n-k-1)!} \\ &\quad = \frac{n!2^{n-4}}{(2n-3)!!}\sum_{k=1}^{n-1}(k-1) = \frac{n-2}{8}\binom{n}{2}\frac{(2n-2)!!}{(2n-3)!!} \end{aligned}$$

So, the independent term of equation (47) is

$$
\begin{aligned}
&3\sum_{k=1}^{n-1} C_{k,n-k}k\binom{k}{2}\frac{(2k-2)!!}{(2k-3)!!} - 2\sum_{k=1}^{n-1} C_{k,n-k}\binom{k}{2}k \\
&\qquad + \sum_{k=1}^{n-1} C_{k,n-k}(n-k)\binom{k}{2}\frac{(2k-2)!!(2(n-k)-2)!!}{(2k-3)!!(2(n-k)-3)!!} \\
&\quad = 3\Big(\frac{n}{2}\binom{n}{2}\frac{(2n-2)!!}{(2n-3)!!} - \frac{2(2n-1)}{3}\binom{n}{2}\Big) \\
&\qquad - 2\Big(\frac{n}{2}\binom{n}{2} - \frac{n(3n-2)}{16}\cdot\frac{(2n-2)!!}{(2n-3)!!}\Big) + \frac{n-2}{8}\binom{n}{2}\frac{(2n-2)!!}{(2n-3)!!} \\
&\quad = \frac{n(13n^2-9n-2)}{16}\cdot\frac{(2n-2)!!}{(2n-3)!!} - \binom{n}{2}(5n-2).
\end{aligned}
$$

In summary, we have proved so far that the sequence $E_U(\Phi_n S_n)$ is the solution of the recurrence

$$
\begin{aligned}
X_n &= 2\sum_{k=1}^{n-1} C_{k,n-k}X_k - (5n-2)\binom{n}{2} + \frac{n(13n^2-9n-2)}{16}\cdot\frac{(2n-2)!!}{(2n-3)!!} \\
&= 2\sum_{k=1}^{n-1} C_{k,n-k}X_k - 15\binom{n}{3} - 8\binom{n}{2} \\
&\qquad + \Big(\frac{39}{8}\binom{n}{3} + \frac{15}{4}\binom{n}{2} + \frac{1}{8}\cdot n\Big)\frac{(2n-2)!!}{(2n-3)!!}
\end{aligned}
$$

with initial condition $X_1 = E_U(\Phi_1 S_1) = 0$. By Proposition 6, this solution is

$$
\begin{aligned}
E_U(\Phi_n S_n) &= \frac{104}{5}\binom{n}{4} + 28\binom{n}{3} + 8\binom{n}{2} - \Big(\frac{45}{8}\binom{n}{3} + 4\binom{n}{2}\Big)\frac{(2n-2)!!}{(2n-3)!!} \\
&= \binom{n}{2}\Big(\frac{26n^2+10n-4}{15} - \frac{15n+2}{8}\cdot\frac{(2n-2)!!}{(2n-3)!!}\Big)
\end{aligned}
$$

Finally,

$$
\begin{aligned}
Cov_U(\Phi_n, S_n) &= E_U(\Phi_n S_n) - E_U(\Phi_n)E_U(S_n) \\
&= \binom{n}{2}\Big(\frac{26n^2+10n-4}{15} - \frac{15n+2}{8}\cdot\frac{(2n-2)!!}{(2n-3)!!}\Big) \\
&\qquad - \frac{1}{2}\binom{n}{2}\Big(\frac{(2n-2)!!}{(2n-3)!!} - 2\Big)n\Big(\frac{(2n-2)!!}{(2n-3)!!} - 1\Big) \\
&= \binom{n}{2}\left(\frac{26n^2-5n-4}{15} - \frac{3n+2}{8}\cdot\frac{(2n-2)!!}{(2n-3)!!} - \frac{n}{2}\Big(\frac{(2n-2)!!}{(2n-3)!!}\Big)^2\right)
\end{aligned}
$$

This completes the proof of Theorem 6.(c). □

SN-13 Proof of a result stated in the Conclusions

In this section we prove the following result.

Proposition 5 *Let $n = 2^m + k$ with $m = \lfloor \log_2(n) \rfloor \geqslant 5$ and $k < 2^m$.*

(a) *If* $k \geqslant 2^m - 29$*, the minimum value of* V *on* $\mathcal{BT}_n^*$ *is reached exactly at the trees of type* F_n*.*

(b) *If* $k = 2^m - 30$*. the minimum value of* V *on* $\mathcal{BT}_n^*$ *is reached exactly at the trees of type* F_n *when* $m \leqslant 7$ *and at the trees of type* $T_{n;6}$ *if* $m \geqslant 8$*.*

Proof Let $n = 2^{m+1} - x$ with $m = \lfloor \log_2(n) \rfloor \geqslant 5$ and $x \leqslant 30$, so that, with our usual notations, $k = 2^m - x$. For every $j \geqslant 1$ and $5 \leqslant l_1 < \cdots < l_j \leqslant m$,

$$k + \sum_{i=1}^{j} (2^{l_i} - 1) \geqslant 2^m - x + 2^5 - 1 > 2^m$$

and therefore, by Lemma 1, every tree $T_{n;l_1,\ldots,l_j}$ with $j \geqslant 1$ has depth $m+2$ and

$$p_1 = 3 \cdot 2^m - k - \sum_{i=1}^{j} (2^{l_i} - 1) = 2^{m+1} + x - \sum_{i=1}^{j} (2^{l_i} - 1).$$

However, recall from that lemma that not every such tree exists: it must also happen that

$$k + \frac{1}{2} \sum_{i=1}^{j} (2^{l_i} - 2) > 2^m, \text{ i.e., } x < \sum_{i=1}^{j} (2^{l_i - 1} - 1).$$

We shall use this restriction later in this proof.

Now, by Lemma 2, and using the notations

$$A(\underline{l}) := \sum_{i=1}^{j} (2^{l_i} - l_i^2 - 1), \quad B(\underline{l}) := \sum_{i=1}^{j} (2^{l_i} - l_i - 1)$$

introduced in at the beginning of Section SN-7, we have that

$$\begin{aligned} W(T_{n;l_1,\ldots,l_j}) &= \frac{1}{n}\Big(n(p_1 + \sum_{i=1}^{j} l_i^2) - (p_1 + \sum_{i=1}^{j} l_i)\Big) \\ &= \frac{1}{2^{m+1} - x}\Big((2^{m+1} - x)\big(2^{m+1} + x - A(\underline{l})\big) - \big(2^{m+1} + x - B(\underline{l})\big)^2\Big) \\ &= \frac{1}{2^{m+1} - x}\big(2^{m+1}(2B(\underline{l}) - A(\underline{l})) - B(\underline{l})^2 - 2x(2^{m+1} + x) + x(2B(\underline{l}) + A(\underline{l}))\big). \end{aligned}$$

Thus,

$$W(T_{n;l_1,\ldots,l_j}) \leqslant W(B_n) = \frac{2k(2^m - k)}{2^m + k} = \frac{2x(2^m - x)}{2^{m+1} - x}$$

if, and only if,

$$2^{m+1}(2B(\underline{l}) - A(\underline{l})) - B(\underline{l})^2 - 2x(2^{m+1} + x) + x(2B(\underline{l}) + A(\underline{l})) \leqslant 2x(2^m - x),$$

which is equivalent to

$$(2^{m+1} - B(\underline{l}))(B(\underline{l}) - 3x) + (2^{m+1} - x)(B(\underline{l}) - A(\underline{l})) \leqslant 0. \tag{48}$$

Since $B(\underline{l}) > A(\underline{l})$ (because $j \geqslant 1$ by assumption) and $B(\underline{l}) \leqslant \sum_{i=5}^{m} 2^i < 2^{m+1}$, and we are assuming that $x \leqslant 30$, inequality (48) implies that

$$(2^{m+1} - B(\underline{l}))(B(\underline{l}) - 90) + (2^{m+1} - 30)(B(\underline{l}) - A(\underline{l})) \leqslant 0. \qquad (49)$$

In this inequality's left-hand side expression, we know that $B(\underline{l}) < 2^{m+1}$ and, since $m \geqslant 5$ and $j \geqslant 1$, $(2^{m+1} - 30)(B(\underline{l}) - A(\underline{l})) > 0$. Moreover, since $2^5 - 5 - 1 = 26$, $2^6 - 6 - 1 = 57$, and $2^7 - 7 - 1 = 120$, it turns out that if some l_i is larger than 6, then $B(\underline{l}) - 90 \geqslant 0$. Therefore, inequality (49) can only hold when $j = 1$ and $l_1 = 5, 6$ and when $j = 2$ and $l_1 = 5, l_2 = 6$.

In summary, if $V(T_{n;l_1,\ldots,l_j}) \leqslant V(B_n)$, then $\{l_1, \ldots, l_j\}$ is either $\{5\}$, $\{6\}$, or $\{5, 6\}$: in all other cases, $V(B_n) < V(T_{n;l_1,\ldots,l_j})$. Let us check now these three remaining cases:

- If $\{l_1, \ldots, l_j\} = \{5\}$, then the necessary condition $x < \sum_{i=1}^{j}(2^{l_i-1} - 1)$ for the existence of $T_{n;5}$ is satisfied only when $x < 15$. But since $B(5) = 2^5-5-1 = 26$, $A(5) = 2^5-5^2-1 = 6$, and $m \geqslant 5$, the left-hand side expression in (48) satisfies that

$$\begin{aligned}(2^{m+1} - 26)(26 - 3x) &+ 20(2^{m+1} - x)\\ &\geqslant (2^6 - 26)(26 - 3x) + 20(2^6 - x) = 2268 - 134x > 0\end{aligned}$$

  when $x < 15$. Therefore, for the range of values of n considered in the statement, when $T_{n;5}$ exists, $V(B_n) < V(T_{n;5})$.
- If $\{l_1, \ldots, l_j\} = \{6\}$, the necessary condition $x < \sum_{i=1}^{j}(2^{l_i-1} - 1)$ for the existence of $T_{n;6}$ is satisfied for every $x \leqslant 30$. In this case, $B(6) = 2^6 - 6 - 1 = 57$ and $A(6) = 2^6 - 6^2 - 1 = 27$, and inequality (48) becomes

$$(2^{m+1} - 57)(57 - 3x) + 30(2^{m+1} - x) \leqslant 0.$$

  Now, a simple computation shows that if $5 \leqslant m \leqslant 7$ and $x \leqslant 30$, this inequality is not satisfied. As far as when $m \geqslant 8$ goes, if $x \leqslant 29$, then

$$(2^{m+1} - 57)(57 - 3x) + 30(2^{m+1} - x) \geqslant (2^9 - 57)(57 - 87) + 30(2^9 - 29) > 0$$

  but when $x = 30$,

$$(2^{m+1} - 57)(57 - 90) + 30(2^{m+1} - 30) = 981 - 3 \cdot 2^{m+1} < 0.$$

  This implies that if $m \geqslant 8$ and $x = 30$, $V(T_{n;6}) < V(B_n)$, while if $m \geqslant 8$ and $x \leqslant 29$ or if $5 \leqslant m \leqslant 7$ and $x \leqslant 30$, $V(B_n) < V(T_{n;6})$.
- If $\{l_1, \ldots, l_j\} = \{5, 6\}$, the necessary condition $x < \sum_{i=1}^{j}(2^{l_i-1} - 1)$ for the existence of $T_{n;5,6}$ is satisfied for every $x \leqslant 30$. But in this case $B(5, 6) = 83$ and $A(5, 6) = 33$, and then, when $m \geqslant 5$, the left-hand side expression in (49) satisfies that

$$\begin{aligned}(2^{m+1} - 83)(83 - 3x) &+ 50(2^{m+1} - x)\\ &\geqslant (2^6 - 83)(83 - 3x) + 50(2^6 - x) = 7x + 1623 > 0\end{aligned}$$

So, in this case inequality (49) does never hold, and therefore $V(B_n) < V(T_{n;5,6})$.

In summary, for every $m \geqslant 5$ and for every $n = 2^{m+1} - x$ with $x \leqslant 30$, $V(B_n) < V(T_{n;l_1,\dots,l_j})$ for every $j \geqslant 1$ and $5 \leqslant l_1 < \cdots < l_l \leqslant m$ except when $m \geqslant 8$ and $x = 30$, in which case $V(T_{n;6}) < V(B_n) < V(T_{n;l_1,\dots,l_j})$ for every other type of trees $T_{n;l_1,\dots,l_j}$ with $j \geqslant 1$. □

References

1. G. Cardona, A. Mir, F. Rosselló. Exact formulas for the variance of several balance indices under the Yule model. Journal of Mathematical Biology 67 (2013), 1833–1846.
2. G. Gasper, M. Rahman. *Basic Hypergeometric Series* (2ond edition). Encyclopedia of Mathematics and its Applications vol. 96. Cambridge University Press (2004).
3. M. Kirkpatrick, M. Slatkin. Searching for evolutionary patterns in the shape of a tree. Evolution 47 (1993), 1171–1181.
4. F. Matsen, Optimization Over a Class of Tree Shape Statistics. IEEE/ACM Trans. Comput. Biol. Bioinformatics, 4 (2007), 506–512.
5. A. Mir, F. Rosselló, L. Rotger. A new balance index for phylogenetic trees. Mathematical Biosciences 241 (2013),125–136
6. M. Petkovsek and H. Wilf and D. Zeilberger, $A = B$, AK Peters Ltd. (1996). Available online at https://www.math.upenn.edu/~wilf/AeqB.html (Last visited, 01/10/2019.
7. J. S. Rogers, Central moments and probability distributions of three measures of phylogenetic tree imbalance. Systematic Biology 45 (1996), 99–110.
